# Systems level mapping of metabolic complexity in *Mycobacterium tuberculosis* to identify high-value drug targets

**DOI:** 10.1186/s12967-014-0263-5

**Published:** 2014-10-11

**Authors:** Rohit Vashisht, Ashwini G Bhat, Shreeram Kushwaha, Anshu Bhardwaj, OSDD Consortium, Samir K Brahmachari

**Affiliations:** CSIR-Open Source Drug Discovery Unit, New Delhi, India; Academy of Scientific and Innovative Research, New Delhi, India; CSIR - Institute of Genomics and Integrative Biology, New Delhi, India

**Keywords:** Systems biology spindle map, Complexity, Bacterial persistence, Mathematical modeling, Metabolic persister genes

## Abstract

**Background:**

The effectiveness of current therapeutic regimens for *Mycobacterium tuberculosis* (*Mtb*) is diminished by the need for prolonged therapy and the rise of drug resistant/tolerant strains. This global health threat, despite decades of basic research and a wealth of legacy knowledge, is due to a lack of systems level understanding that can innovate the process of fast acting and high efficacy drug discovery.

**Methods:**

The enhanced functional annotations of the *Mtb* genome, which were previously obtained through a crowd sourcing approach was used to reconstruct the metabolic network of *Mtb* in a bottom up manner. We represent this information by developing a novel Systems Biology Spindle Map of Metabolism (SBSM) and comprehend its static and dynamic structure using various computational approaches based on simulation and design.

**Results:**

The reconstructed metabolism of *Mtb* encompasses 961 metabolites, involved in 1152 reactions catalyzed by 890 protein coding genes, organized into 50 pathways. By accounting for static and dynamic analysis of SBSM in *Mtb* we identified various critical proteins required for the growth and survival of bacteria. Further, we assessed the potential of these proteins as putative drug targets that are fast acting and less toxic. Further, we formulate a novel concept of metabolic persister genes (MPGs) and compared our predictions with published in vitro and in vivo experimental evidence. Through such analyses, we report for the first time that de novo biosynthesis of NAD may give rise to bacterial persistence in *Mtb* under conditions of metabolic stress induced by conventional anti-tuberculosis therapy. We propose such MPG’s as potential combination of drug targets for existing antibiotics that can improve their efficacy and efficiency for drug tolerant bacteria.

**Conclusion:**

The systems level framework formulated by us to identify potential non-toxic drug targets and strategies to circumvent the issue of bacterial persistence can substantially aid in the process of TB drug discovery and translational research.

**Electronic supplementary material:**

The online version of this article (doi:10.1186/s12967-014-0263-5) contains supplementary material, which is available to authorized users.

## Background

The first decade of the post-human genome era, with an increasing convergence of experimental and computational approaches, has witnessed a remarkable transformations in our basic understanding of human health and disease [1], however its full impact on rapid diagnosis and cure for long standing neglected diseases such as Tuberculosis is yet to be realized [2]. There is still an unmet need for comprehensive systems-level models that can predict the behavior of living systems. Given our current understanding and available tools, it seems possible to build such systems level models for a relatively moderate complexity of a prokaryote genome and in this article we focus on establishing a comprehensive data intensive systems level understanding of *Mycobacterium tuberculosis* (*Mtb*) genome. We focus on the identification of potential non-toxic drug targets and propose a framework to address the problem of bacterial persistence in *Mtb*. Such methods are expected to potentiate the ongoing efforts of Tuberculosis (TB) drug discovery.

*Mtb* is an etiological agent of TB, which is sweeping the developing world and has become a potential threat to global health [3]. The increasing prevalence of drug resistance TB, as defined in terms of multi-drug resistant (MDR) and extensively drug resistant (XDR) strains of *Mtb*, is emerging as a predominant cause of concern to public health [4]. The multifaceted issues pertaining to drug development, vaccine, transmission, epidemiology and diagnosis of TB have been extensively discussed earlier [5]. While the problem of drug resistance in *Mtb* has been extensively studied [6-9], the aspects of drug tolerance through the emergence of bacterial persistence are seldom addressed. The phenomenon that allows non-mutant pathogens of an isogenic population to survive the impact of an antibiotic is known as bacterial persistence [10-12]. For clarity, it is important to distinguish between persistence and resistance. The latter also reduces the effectiveness of antibiotics, but does so by selecting mutants that evade antimicrobial activity through strategies such as drug efflux [13], gene amplification [14], reduced expression of targets [15], and structural modulation of drug-binding enzymes [16]. The impact of heterogeneity in the metabolism of a given pathogen towards the formation of persister phenotypes that can demonstrate drug tolerance, however remains elusive. In the light of World Health Organization (WHO) recent warning, ‘*The world is poised to enter a post*-*antibiotic era*’, [17] the current situation of *Mtb* drug resistance and drug tolerance therefore unequivocally suggests an urgent need for the development of new therapeutic interventions and strategies to tackle the problem of TB.

The exponential rise of big data in biological science in recent years has crystalized the idea of data-driven drug discovery [18]. The basic component of a data-intensive framework for drug discovery can be classified into *data capture*, *data curation*, *data visualization* and *hypothesis driven data analysis*. We have implemented certain aspects of this new paradigm of data-driven drug discovery earlier as a framework in Open Source Drug Discovery (OSDD) [19,20] that utilizes various crowdsourcing approaches to capture data in terms of manual genome annotation and innovative strategies towards its curation, visualization and hypothesis driven analysis [21,22].

In the present work, we formulate a data-intensive systems level framework for the analysis of *Mtb* genome as data curation, data visualization and hypothesis driven data analysis to identify potential non-toxic drug targets and comprehend the metabolic basis of bacterial persistence in the context of drug discovery. We begin our analysis by manually curating and updating the metabolic knowledgebase of *Mtb* based on comprehensive manual re-annotation of its genome that was earlier undertaken by us [21,22]. Further we developed a novel visualization method termed as Systems Biology Spindle Map (SBSM) to represent the metabolism of *Mtb*. SBSM reduces the visual complexity of the problem significantly and facilitates its empirical analysis. Further, by modeling the structure and dynamics of SBSM in *Mtb* we elucidate various critical genes that are likely to be essential for its growth and survival, and assess them as putative non-toxic drug targets in a hypothesis driven manner. Furthermore, we hypothesize a novel concept of Metabolic Persister Genes (MPGs) that may give rise to a persistence phenotype of *Mtb* resulting into drug tolerance. On the basis of our findings we build a spectrum of such MPGs in *Mtb* under the selection pressure of front line antibiotcs such as Isoniazid, Ethambutol, Rifampicin and TCA1 administer to treat TB and propose alternate drug targets. We provide substantial amount of experimental evidences both *in vitro* and *in vivo* by referring to a wealth of literature information to assess the potential of predicted drug targets. Most of our findings are consistent with the available experimental evidence *in vitro* and *in vivo*. We believe that the novel systems-level strategies developed in this article and the results obtained can advance our basic understanding of *Mtb* metabolic physiology and provide a framework towards developing new therapeutic interventions for targeting drug resistance and drug tolerance due to bacterial persistence in *Mtb*.

## Methods

This study uses the functional re-annotation of the *Mtb* genome, which we previously reported [22,23]. The iNJ661 reconstruction was our starting point [24]. Its inconsistencies were removed, and additional gene-reaction associations were incorporated from various databases such as KEGG, Biocyc, MetaCyc, SEED as well as reference textbooks from PubMed (Additional file 1: Table S1A-C for detailed references). This resulted in the *iOSDD890* reconstruction. Every new reaction was charged and mass-balanced based on its stoichiometric parameters, and Flux-Based Analysis (FBA) was performed in order to assess its contribution to the objective biomass function [25]. An overall biomass objective function was used as defined in iNJ661 [24]. All the data was captured and organized into Excel® spreadsheets, which were then converted into a metabolic model using MATLAB®. The FBA was performed on the stoichiometric matrix S of *iOSDD890* as described below:

$$ \underset{v}{\operatorname{} \max \operatorname{}\kern0.1em }\kern0.1em {c}^Tv $$

$$ s.t\kern1em S.v=0 $$

$$ subject\kern0.5em to\kern0.1em :\kern0.1em {v}_{min}\le {v}_i\le {v}_{max} $$

Where *S* is (*m x n*) stoichiometric matrix representing *m* metabolites and *n* reactions, *v* is (*n x 1*) the flux vector, *c* represents the objective function weight in terms of flux vector *v. v*_*min*_ and *v*_*max*_ are the constraints on the system. The overall biomass was defined as *c*^*T*^*v*_._

### Systems biology spindle Map

Systems biology spindle maps were generated using 3-degree Bézier curves defined by:

$$ B(t) = {\displaystyle \sum_{i=0}}\left(\begin{array}{c}\hfill n\hfill \\ {}\hfill i\hfill \end{array}\right){\left(1-t\right)}^{n-i}{t}^i{P}_i\kern0.5em t\in \left[0,\ 1\right] $$

where: Function B(t) traces its path from control points *P*_*0*_, *P*_*1*_, *P*_*2*_, *P*_*3*_*for n* = *3*. The topological connectivity of all the metabolites, genes and reactions was computed using the genome scale metabolic matrix in *Mtb*.

### Directional re-routing of metabolic fluxes

The re-routing of metabolic fluxes was computed for every gene knock out as a binary matrix for all the knockouts, defined as:

$$ {\left[DRM\right]}_{ij}=\left\{\begin{array}{c}\hfill 1\kern0.5em \hfill \\ {}\hfill 0\hfill \end{array}\right. $$

where: *DRM*_*ij*_ = *1* if the reaction was observed to carry flux in knock-out condition but not in optimal condition and *DRM*_*ij*_ = *0* otherwise.

### Reaction-reaction graph and module identification

The reaction-reaction graph was computed from the stoichiometric matrix that takes the reversibility of reactions into account. The nodes in the graph are reactions and they are connected if they shared a common metabolite. Hence two nodes are connected if they share a metabolite produced by one and consumed by the other. This produced a reaction-reaction (*n x n*) matrix [RRA]. For [*RRA*]_*ij*_ the weights of the *i*^*th*^ row and *j*^*th*^ column are defined as:

$$ {\left[RRA\right]}_{ij}=\left\{\begin{array}{c}\hfill 1\kern0.5em \hfill \\ {}\hfill 0\hfill \end{array}\right. $$

where: RRA_ij_ = 1 if the metabolite is produced by the first reaction and consumed by the second and RRA_ij_ = 0 otherwise.

### First neighborhood topological overlap of [RRA]

The modules in the metabolic network were identified by first computing the topological overlap matrix of [*RRA*]_*ij*_. For a given graph, the topological overlap matrix is bounded between value 0 and 1. The value 0 means no topological overlap in the sense that any two given nodes are not connected and they do not share any direct common neighbor. The value 1 means high overlaps if there is a direct link between two given nodes and if one set of direct neighbors is the subset of the other. For [*RRA*]_*ij*_, two reactions were therefore regarded similar based on their topological overlap as follows:

$$ {T}_O\left(i,j\right)=\left\{\begin{array}{lll}\frac{\left|{\alpha}_n(i)\cap {\alpha}_n(j)\right|+{\left[RRA\right]}_{ij}}{min\left\{\left|{\alpha}_n(i)\right|,\left|{\alpha}_n(j)\right|\right\}+1-{\left[RRA\right]}_{ij}}\hfill & \hfill & if\kern0.5em i\ne j\hfill \\ {}1\hfill & \hfill & if\kern0.5em i=j\hfill \end{array}\right. $$

where: *T*_*O*_(*i*, *j*) is the topological overlap matrix, *α*_*n*_ (*i*) represents the direct neighbor of *i* for all *i*, *α*_*n*_ (*j*) represents the direct neighbor of *j* for all *j*, the quantity |*α*_*n*_(*i*) ∩ *α*_*n*_(*j*)| measures the total number of common neighbours that node *i* and *j* share. For *n* = *1*, |*α*_*n*_(*i*)|, |*α*_*n*_(*j*)| represents the total number of neighbours of node *i* and *j* for *n* = *1* respectively and [*RRA*]_*ij*_ ithe reaction-reaction adjacency matrix.

### Module identification

The modules were identified based on the un-weighted average distance method applied to the computed topological overlap matrix *T*_*O*_(*i*, *j*) previously defined. Briefly, any two reactions x_r_ ∈ *R and* ×_s_ ∈ *S* represented by nodes in the *T*_*O*_(*i*, *j*) matrix with the highest overlap value were first joined together to a branching point on the tree and indexed as a new cluster, which was then joined with the subsequent branches of a tree with other clusters with similar topological overlap as follows:

$$ d\left(R,S\right)=\frac{1}{n_r{n}_s}{\displaystyle {\sum}_{i=1}^{n_r}}{\displaystyle {\sum}_{j=1}^{n_s}} dist\left({x}_{ri},{x}_{sj}\right) $$

Where: *dist*(*x*_*ri*_, *x*_*sj*_) is the distance between objects *x*_*ri*_ ∈ *R* and *x*_*sj*_ ∈ *S*, *R and S* are the two sets of objects (clusters), *n*_*r*_*n*_*s*_ are the total number of objectes in the cluster *R and S* respectively.

## Results

### Structuring biochemical, genetic and genomic knowledgebase of *Mtb*

Decades of TB research has resulted in a wealth of information, which can be attributed to unstructured form of information, available as various journal articles in PubMed [21]. A simple search in PubMed with the key word ‘*mycobacterium tuberculosis*’ results in numerous papers – the unstructured information, the rate of which rises exponentially every year since the last re-annotaiton of its genome was reported in 2002 [26]. The potential of this unstructured information available for *Mtb* remains untapped, which if utilized could substantially improve the functional annotation of its genome and quality of subsequent genome scale reconstruction of metabolism. The initial sequencing of *Mtb* genome a decade ago [27], and its subsequent annotation till 2002 [26] have been utilized to construct two comprehensive genome scale reconstructions of its metabolism [24,28]. Although useful to a large extent these reconstructions primarily rely on the partial annotation (52%) of the *Mtb* genome available based on its most recent re-annotation statistics [26], thus leaving a gap in our understanding of metabolic physiology in *Mtb*. We filled this gap by crowdsourcing the genome annotation of *Mtb* by referring to ~27,000 published manuscripts that resulted in 87% of functional annotations as reported by us earlier [22].

**Figure 1:**
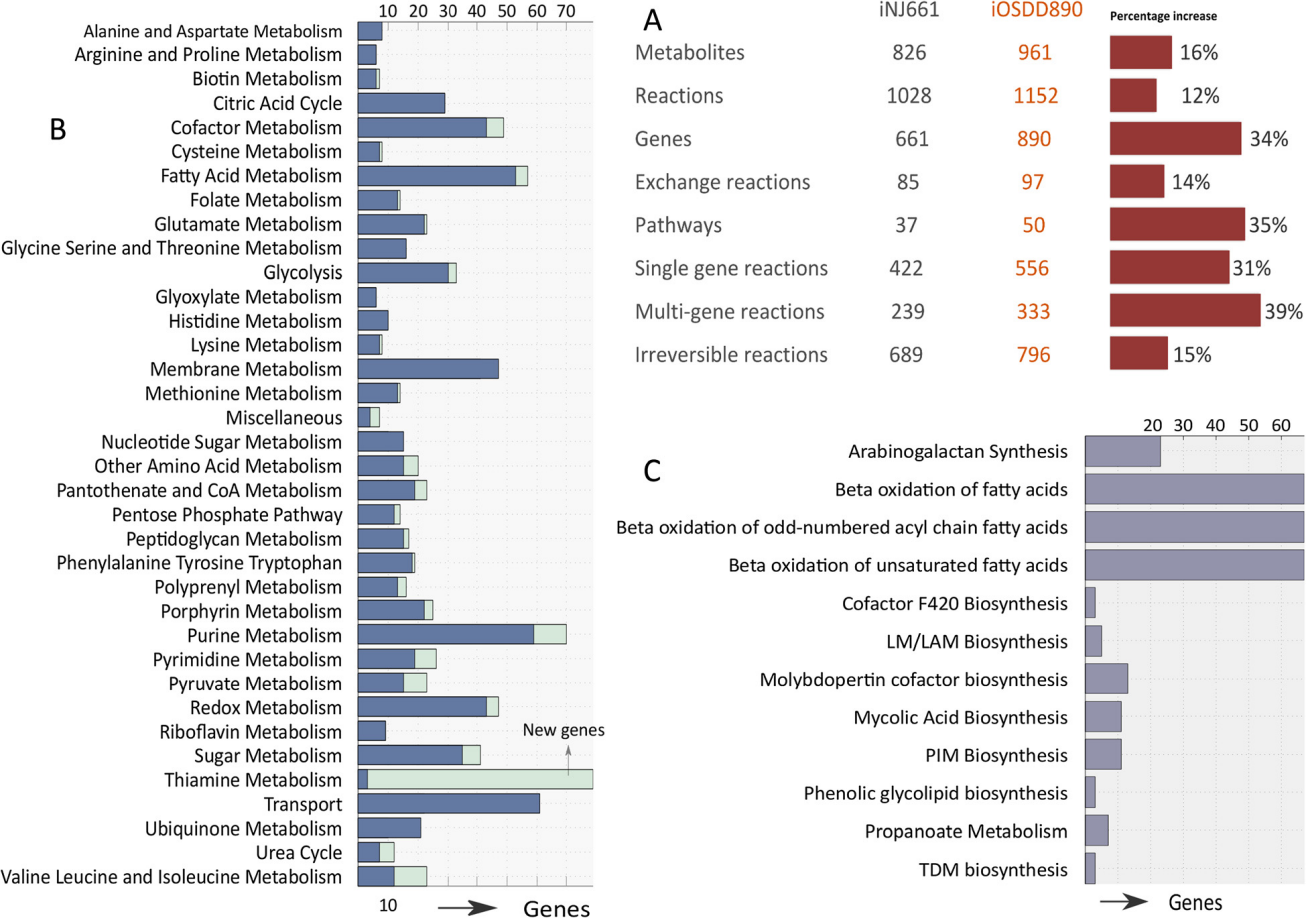
**Biochemical, genetic and genomic knowledgebase of** ***Mtb*** **- iOSDD890. A)** Profile of metabolism in Mtb based on iOSDD890 reconstruction and its comparision with iNJ661; **B)** Number of new genes added to iNJ661; **C)** Number of new pathways and respective genes.

The enhanced functional annotations of *Mtb* genome were utilized to reconstruct its metabolism at genome scale in a bottom up manner. In order to formalize the best capabilities and to fill the knowledge gap in the metabolism of *Mtb* we followed standard and well established protocol for its reconstruction (Methods) [25] and the resulting GENRE of *Mtb* was labeled as *iOSDD890* as illustrated in (Figure 1). In contrast to prior comprehensive genome-scale reconstruction of *Mtb* (iNJ661) [24], the iOSDD890 contains 16% more metabolites, 12% more reactions and 34% more genes reflecting a significantly improved knowledgebase of the metabolism in *Mtb* (Figure 1A). A comparison of iOSDD890 with other genome scale reconstructions of *Mtb* GSMN-TB [28], iNJ661 [24] and iNJ661m [29] revealed a significant improvement in the metabolite, gene and reaction coverage of iOSDD890 (see Additional file 2: Figure S1).

Our understanding of the pathways involved in thiamine, valine, leucine and isoleucine biosynthesis was substantially expanded in terms of their gene coverage (Figure 1B). New pathways such as β-oxidation of fatty acids, β-oxidation of odd numbered acyl chain fatty acids, β-oxidation of unsaturated fatty acids were added along with a substantial number of genes (Figure 1C). β-oxidation of fatty acids is important in terms of lipid utilization. While much of the earlier work has been focused on lipid biosynthesis, lipid degradation through β-oxidation has been less explored. Since lipids are an important source of energy in hypoxic conditions, integrating these pathways into the model helps us understand the survival of *Mtb* within the hypoxic niches of granulomas. The other important pathway added to the model is the biosynthesis of the cofactor F420. The cofactor F420 is deazaflavin with no homology in humans but it is widespread across prokaryotes such as *Mycobacteria*. It has been suggested that the cofactor F420 plays an important role in shielding *Mtb* from NO_2_ stress, as induced by macrophage in aerobic conditions [30]. The detailed biochemical information, comprising the new genes, reactions, pathways and the literature cited included in *iOSDD890*, together with the supportive literature is provided in (see Additional file 1: Table S1 (A-C)) and the model (iOSDD890) is provided in (Additional file 3: Model-01).

### Systems Biology Spindle Map

To comprehend the complex metabolic architecture one must understand its organization in terms of metabolites and genes that encode proteins with respect to the reactions involved in various pathways. Conventionally, metabolic pathways are represented in KEGG and other common formats based on flow chart diagrams [31]. While useful to a great extent, such representations seldom account for inherent structural connectivity among various metabolites, the genomic organization of genes (encoding for enzymes), reactions and pathways. For instance, the metabolic representation that can display the relation between various metabolites involved in various reactions with respect to the chromosome location of genes (encoding for respective enzymes) is absent. The conventional flow chart diagrams therefore limited our understanding of metabolic structure, in the context of its function. This lack of accountability in inherent metabolic topology by conventional representation also makes it formidable to construct efficient computational algorithms that can render complete metabolic information in a human perceivable manner.

**Figure 2:**
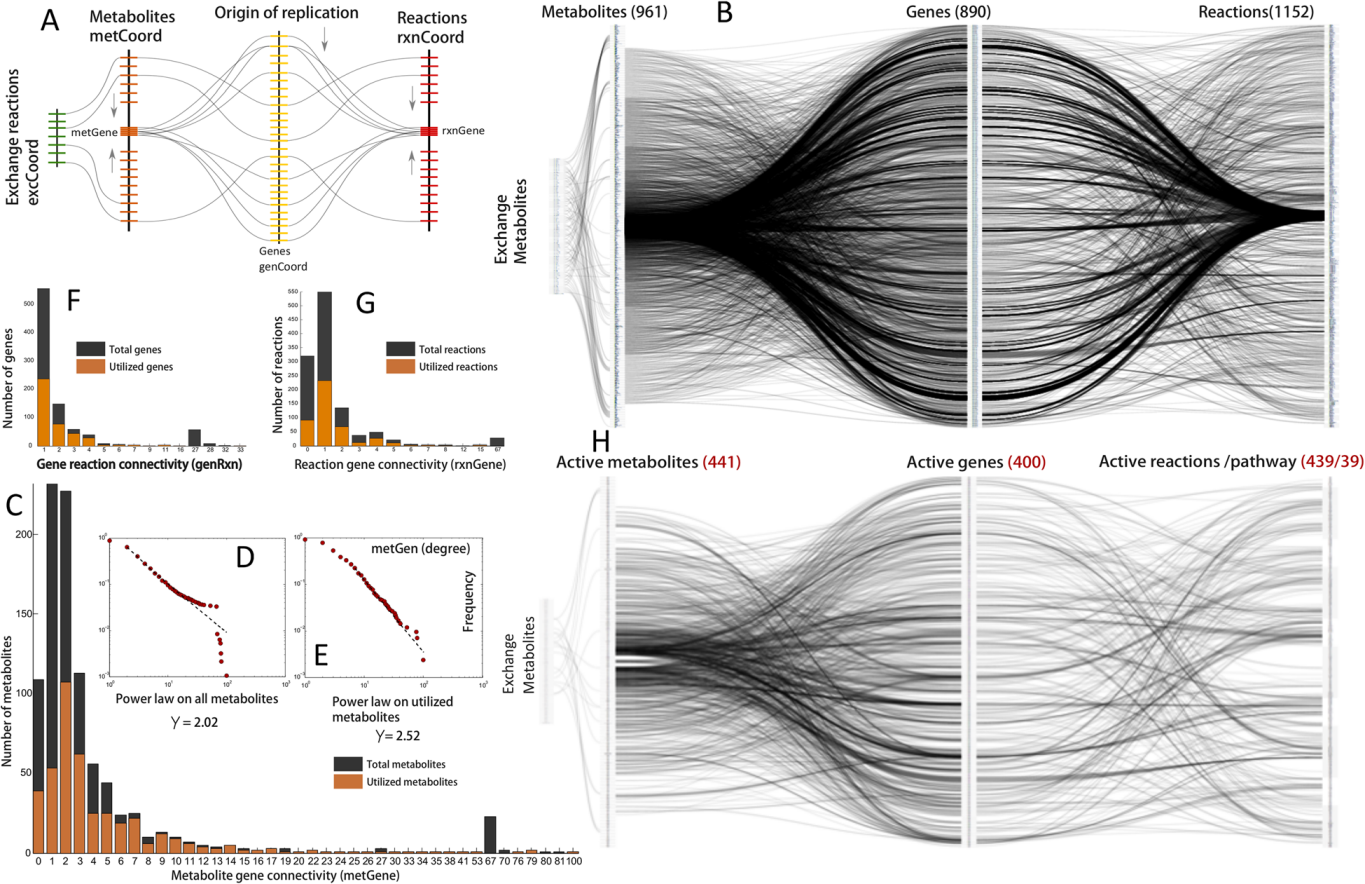
**Metabolic visualization through** ***Systems Biology Spindle Map*** **(** ***SBSM).*** **A)** Conceptual formulation of SBSM representing the connection between exchange reactions, metabolites, genes and reactions; **B)** SBSM of *Mtb* representing its complete metabolic topology based upon the relationships between its biochemical, genomic and genetic information; **C)** Metabolite gene connectivity (*metGene*) distribution. Overall connectivity versus the connectivity of utilized metabolites in optimal metabolic physiology; **D)** Power law distribution of overall connectivity of metabolites to genes in SBSM; **E)** Power law distribution of active metabolite connectivity to genes in optimal SBSM **F)** Gene reaction (*geneRxn*) connectivity distribution. Overall connectivity versus the connectivity of utilized genes; **G)** Reaction gene (*rxnGene*) connectivity distribution. Overall connectivity versus the connectivity of active reactions **H)** optimal metabolic physiology obtained by optimizing for defined biomass function using Middlebrook media.

To circumvent these challenges we developed a new method of metabolic visualization termed as Systems Biology Spindle Map (SBSM). As illustrated in (Figure 2A), the components of SBSM include metabolites, genes and reactions that are arrayed with their respective pathways parallel to each other. The metabolites are classified into exchange and intracellular metabolites. The exchange metabolites are linked to intracellular metabolites by exchange reactions, representing all possible ways in which a given metabolite can be imported or exported, into or from the system.

All the metabolic genes are placed according to their genomic order (chromosome position) with the first gene (top) being closer to the origin of replication as illustrated in (Figure 2A). The metabolites are then connected to respective genes based on the reactions in which they either act as reactant or product. The metabolites are sorted according to *metGene*, which represents the metabolite out-degree with respect to genes as illustrated in (Figure 2A). The metabolite with the highest *metGene* is placed at the centre, and each subsequent metabolite is placed alternatively on either side of the central metabolite, in descending order of *metGene* as illustrated in (Figure 2A). The reactions however, were first classified into their respective pathways and then arranged according to reaction-gene (*rxnGene*) connectivity (representing reaction out degree with respect to genes) similar to the placement of metabolites as illustrated in (Figure 2A). The resultant complete metabolic information in *Mtb* is summarized in (Figure 2B).

In addition, the degree distribution of the tripartite graph linking metabolites, genes and reactions was analyzed. Most of the metabolites were found to be highly connected to either one or two genes as illustrated in (Figure 2C) suggesting less redundancy in their utilization as either reactant or product. In contrast to currency metabolites, metabolites such as acetyl-CoA were found to be highly connected (100 genes) as illustrated in (Figure 2C). Metabolites such as octacosanoyl-CoA, hexacosanoyl-CoA, tetracosanoyl-CoA, docosanoyl-CoA etc. were found to be connected to a specific set of 67 genes, as illustrated in (Figure 2C), predominantly coding for the FAD operon involved in the β-oxidation of fatty acids. About 100 metabolites were found to be unconnected to any gene. The list of all the metabolites and their respective gene connectivity is provided in (Additional file 1: Table S2). The gene-reaction connectivity analysis revealed skewness towards single gene reactions, as illustrated in (Figure 2F), suggesting specificity in their function. A total of 57 genes were found to connect to a specific set of 27 reactions, as illustrated in (Figure 2F), involved in the β-oxidation of fatty acids. The complete list of genes and the reactions to which they are connected is provided in (see Additional file 1: Table S3). A total of 550 reactions were observed to be catalyzed by a single gene, and 320 were unconnected to any gene (Figure 2G). These orphan reactions along with their respective metabolites suggest a knowledge gap that remains to be filled by future reaction annotations.

### Context dependent metabolism in *Mtb*

While the complete SBSM of metabolism in *Mtb* (Figure 2B) depicts an overall connectivity between various metabolites, genes and reactions, it also indicates that there might exist a functional redundancy in terms of its connectivity. For a given fitness function, such functional redundancy can be computed as a subset of the overall connectivity of SBSM in *Mtb* in a context dependent manner. To this end, we used an optimization approach (flux balance analysis) to quantify the optimal set of metabolic physiology in *Mtb* for a given fitness function (biomass) defined for specific media condition (Middlebrook) (see Methods) [32]. The *in silico* growth rate that was predicted on the basis of our simulations was found to be well in accordance with the growth rates reported in the literature, thereby validating the model [33,34]. The quantified model of metabolism in *Mtb* was further used for all the analysis.

The reduced overall connectivity of SBSM in *Mtb* computed as an optimal set of metabolic information required for growth on Middlebrook media is illustrated in (Figure 2H), suggesting that *Mtb* uses only a subset of its metabolism to perform optimally. Interestingly, our model showed that only 441 (45%) metabolites (Figure 2C), 439 (38%) reactions (Figure 2G) and 400 (44%) (Figure 2F) genes were active in terms of their flux carrying capacity. On an average, the metabolites with high *metGen* were found to be active as illustrated in (Figure 2C). Most of the metabolites with a *metGen* degree of 2 were found to be predominantly active in contrast to other metabolites whose *metGen* ranges from 0 to 7 (Figure 2C). The metabolites belonging to pathways such as β-oxidation of fatty acids (connected to 67 genes of FAD operon) were mostly observed to be inactive in the optimized metabolic physiology of *Mtb*, further suggesting their importance for a condition dependent metabolism.

Interestingly, we observed a more exact power-law distribution (γ = 2.52) on *metGen* for active metabolites in contrast to power law distribution of overall *metGen* (γ = 2.02) of SBSM (Figure 2D-E), suggesting a modular pattern in the utilization of metabolites in the metabolism of *Mtb* akin to what has been observed for other bacterial species [35]. The improved value of γ suggests that *Mtb* uses its most connected metabolites to operate in an optimal manner. A similar type of pattern was observed in the utilization of genes and reactions, where genes and reaction with connectivity in the range of 0 to 4 were found to be mostly active (Figure 2F-G). The 400 active genes were then compared with the active transcriptome of *Mtb*, as recently reported [36]. A similarity of 91% (364 genes) was found between the active transcriptome (rpkm > 5) and predicted active genes in our model. Predominantly, genes belonging to operons such as *sdhA*-*D*, *mmaA1*-*A4*, *sugA*-*C*, *atpA*-*F*, *murA*-*I*, *pyrA*-*H*, *cydA*-*D*, *aroA*,*B*,*D*,*E*,*G*,*F*,*K*, *ppsA*-*E* and *mur enzymes* were found to be active. The complete list of model genes, active genes and the rpkm value of active gene transcript is provided in the (Additional file 1: Table S4).

### Critical genes required for the growth of *Mtb*

Elucidating the most efficient metabolic architecture has given us a roadmap to identify new drug targets. It helps us identify the essential genes that are required to maintain the metabolic integrity of bacteria. A gene was regarded as essential if its knockout resulted into no biomass production. The genes were classified into three categories of lethality: (a) *lethal genes*: genes whose knockout resulted in no growth; (b) *enzymatically low efficient genes* (ELE): genes that result in more flux utilization than the alternate pathway to achieve the same predicted growth rate. Such genes therefore need to be overexpressed in order to achieve the desired growth rate; (c) *metabolically low efficient genes* (MLE): genes that reduce the growth if they are used in a pathway.

Lethal genes were computed based on single gene knockout of the genes involved in optimal metabolic physiology as illustrated in (Figure 2F). Both the ELE and MLE class of genes were identified based on the most efficient metabolic physiology as computed using the pFBA approach [37]. The pFBA method relies on selecting the fastest growing mutant with a minimal set of metabolic usage for a given objective function, which results in the elucidation of the most efficient metabolic physiology. The predicted essential genes were further assessed based on the experimental evidence for their essentiality from both *in vitro* and *in vivo* literature. Towards this we constructed a literature based evidence pool of gene essentiality by referring to a vast amount (~112) of different manuscripts reporting the essentiality of *Mtb* genes *in vitro*, *in vivo*, *in macrophage* and *in mice* models, in contrast to previous investigations [24,28], wherein the comparison between computational predictions to that of their experimental validations has been primarily made using single mutagenesis based studies.

**Figure 3:**
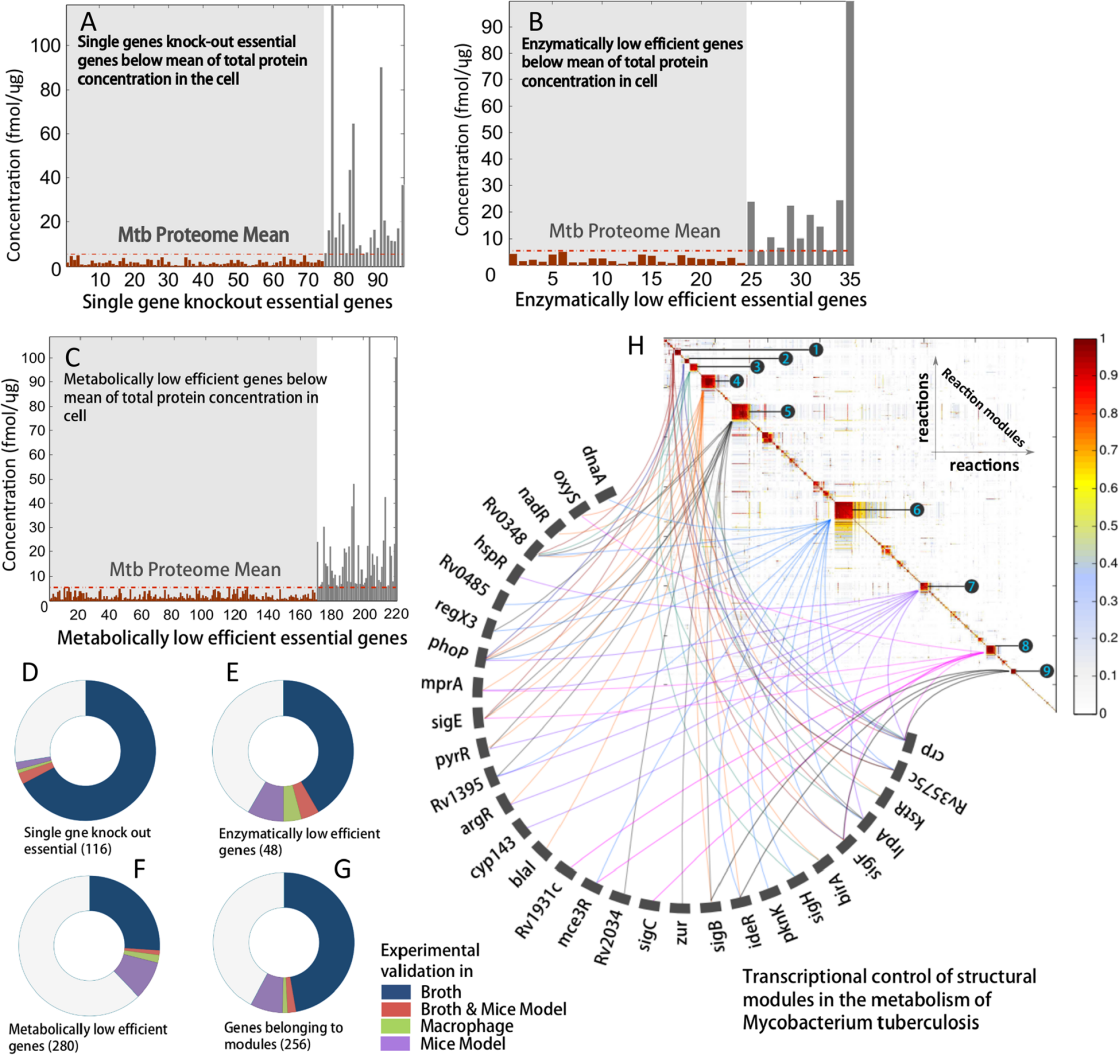
**Essential genes in the metabolism of** ***Mtb*** **required for its growth and survival. A)** Protein concentration of single gene knock-out lethal genes, **(B)** enzymatically low efficient genes and **(C)** metabolically low efficient genes with respect to mean abundance of complete *Mtb* proteome. Literature based qualitative assessment of **(D)** single gene knock-out essential genes, **(E)** enzymatically low efficient genes, **(F)** metabolically low efficient genes and **(G)** genes belonging to all the structural modules respectively **(H)** transcriptional control of genes mapped to various reaction topological modules on reaction-reaction graph of *Mtb* metabolism.

Knocking-out genes *in*-*silico* one at a time resulted into 116 lethal genes (Figure 3D, Additional file 1: Table S5). Of these, 86 (~74%) genes were also found to be essential based on *in vitro*, *in vivo*, *in macrophage* and *in mice* model evidence from the constructed literature pool. For enzymatically low efficient genes, a total of 48 of the 890 genes were found to be ELE essential (Figure 3E, Additional file 1: Table S6). Of these 48, 28 (58%) were reported to be essential in the *in vitro*, *in vivo*, *in macrophage* and *in mice* model based on the literature evidence pool. The genes that reduced the growth rate if they participate in metabolic pathways were then identified as metabolically low efficient genes (MLE). Of the 890 metabolic genes, 281 genes were found to be MLE essential (Figure 3F, Additional file 1: Table S7). Of these, 107 (38%) were also reported to be essential *in vitro*, *in vivo*, *in macrophage* and *in mice* model based on the literature evidence pool. For all the essential genes, a 58% overlap was observed between *in*-*silico* predictions and what has been reported in the literature evidence. However, the overlap was 74% between our *in*-*silico* predictions and the existing experimental results based on the *in vitro*, *in vivo*, *in macrophage* and *in mice* model, suggesting a significant improvement over previous research where the *in silico* predictions have been predominantly compared with only *in vitro* analysis.

The minimum inhibitory concentration (MIC) of an antibiotic is an important measure of its efficacy. All other things being equal, the lower the concentration of a target protein/enzyme for a non-competitive inhibitor or a tight binder (as most of the known antibacterial compounds are non-competitive inhibitors), the lower the antibiotic's MIC and hence more will be its efficacy. In this context, we assessed the intracellular levels of all the proteins/enzymes coded by the predicted essential genes based on the complete proteome of *Mtb* as reported recently [38], and illustrated in (Figure 3A,B,C). The mean concentration of the complete set of proteins of *Mtb* was computed, and used to assess the concentration of the proteins derived from essential genes. Most of these protein/enzymes had a lower concentration than the mean protein concentration. Of the total 116 single knockout lethal genes, 97 genes were expressed, resulting in enzymes of measurable concentration. 75 of these expressed genes resulted in protein/enzyme concentrations lower than the total mean concentration of the *Mtb* proteome. Similarly, the protein concentration and respective details of single gene knockout essential genes, ELE and MLE essential genes as illustrated in (Figure 3B-C) is provided in (see Additional file 1: Table S8).

### Metabolic network structure and function

The topological location of metabolites, reactions and genes can be used to assess the efficiency of a metabolic structure. It has been suggested that metabolism operates in a modular manner [35]. Each module can be identified based on its function, such as genes involved in cofactor-membrane-methionine-mycolic acid and ubuquinone metabolism; fatty acids-glycolysis-citric acid cycle and sugar metabolism. Given the importance to *Mtb* of such specialized pathways as mycolic acid and lipid production, it seems important to analyze its modular topology, and identify its vulnerabilities. In this context we hypothesize that the transcription factors regulating genes that belong to different modules could be essential targets, as their inhibition would be more likely to result in lethal metabolic disruption.

We identified the modules in the metabolic network of *Mtb*. Towards this a reaction-reaction graph (directed) was first constructed. The graph was then partitioned into various modules by computing the topological overlap matrix that was quantified based on hierarchal clustering [35] (Methods). A total of 9 modules were identified as illustrated in (Figure 3H). The reactions of each module were mapped to their respective genes, resulting in sets of specific genes that were unique to each module (see Additional file 1: Table-S9A-I). The genes of each module were further mapped to their respective transcription factors, as illustrated in (Figure 3H). Among all the transcription factors *phoP*, *Rv0348* and *sigE* were observed to be highly connected to genes belonging to different modules. *phoP*, which regulates a two-component system, is essential for maintaining virulence [39]. *mosR* (*Rv0348*) is a unique transcription factor that plays a key role in the maintenance of *Mtb*’s hypoxic state by up-regulating the *mce1* operon, which is responsible for mammalian cell entry and for regulating various genes involved in hypoxia and starvation [40].

*sigE* is one of the sigma factors in *Mtb*, which is thought to be responsible for the heat shock response. *sigE* mutants are more sensitive to heat shock and various oxidative stress conditions [41]. Our earlier interactome analysis suggests *sigE* as a potential drug target candidate [22]. This analysis confirms this finding and suggests that *phoP* and *mosR* are other valuable targets whose inhibition could severely disrupt *Mtb* metabolism. The list of all the transcription factors that were mapped to respective genes in various modules along with their pathways is provided in (Additional file 1: Table-S9A-I).

### Metabolic basis of emergence in persister phenotype

The phenomenon in which an isogenic population of bacterial cells survives the impact of an antibiotic is defined as bacterial persistence [10-12]. While bacterial persistence has been predominantly investigated in *Escherichia coli*, its possible implications in *Mtb* are seldom addressed except in a recent investigation on *Mycobacterium smegmatis* [42]. The precise mechanism leading to persister formation remains elusive, however investigations suggest a predominant role of phenotypic heterogeneity [10,43,44], SOS response induced by DNA damage [45], Bet hedging [46] and stochasticity in cellular regulation [47,48] as probable mechanism linked to bacterial persistence.

It is unclear if heterogeneity in the metabolism, that can be brought about by the complex connectivity among various metabolites, genes and reactions can facilitate formation of the persister. For a given metabolic structure, such as those represented by SBSM of *Mtb*, one can decipher the metabolic heterogeneity in terms of alternate metabolic sub-phenotypes that bacteria can adopt to survive in a context dependent manner under the influence of a given selection pressure. Such selected metabolic sub-phenotypes might become stable over a prolonged period of stress, which eventually may facilitate the emergence of persister phenotypes. Moreover, the intricate connectivity of metabolic interactions makes it likely that the selection of metabolic sub-phenotypes will be the preferred adaptive response, as it is more efficient than altering the genomic architecture through mutations. In this context, we therefore hypothesize that the directional re-routing of metabolic fluxes as a putative adaptive mechanism responsible for section of metabolic sub-phenotypes under antibiotic stress that may give rise to bacterial persistence in *Mtb*.

**Figure 4:**
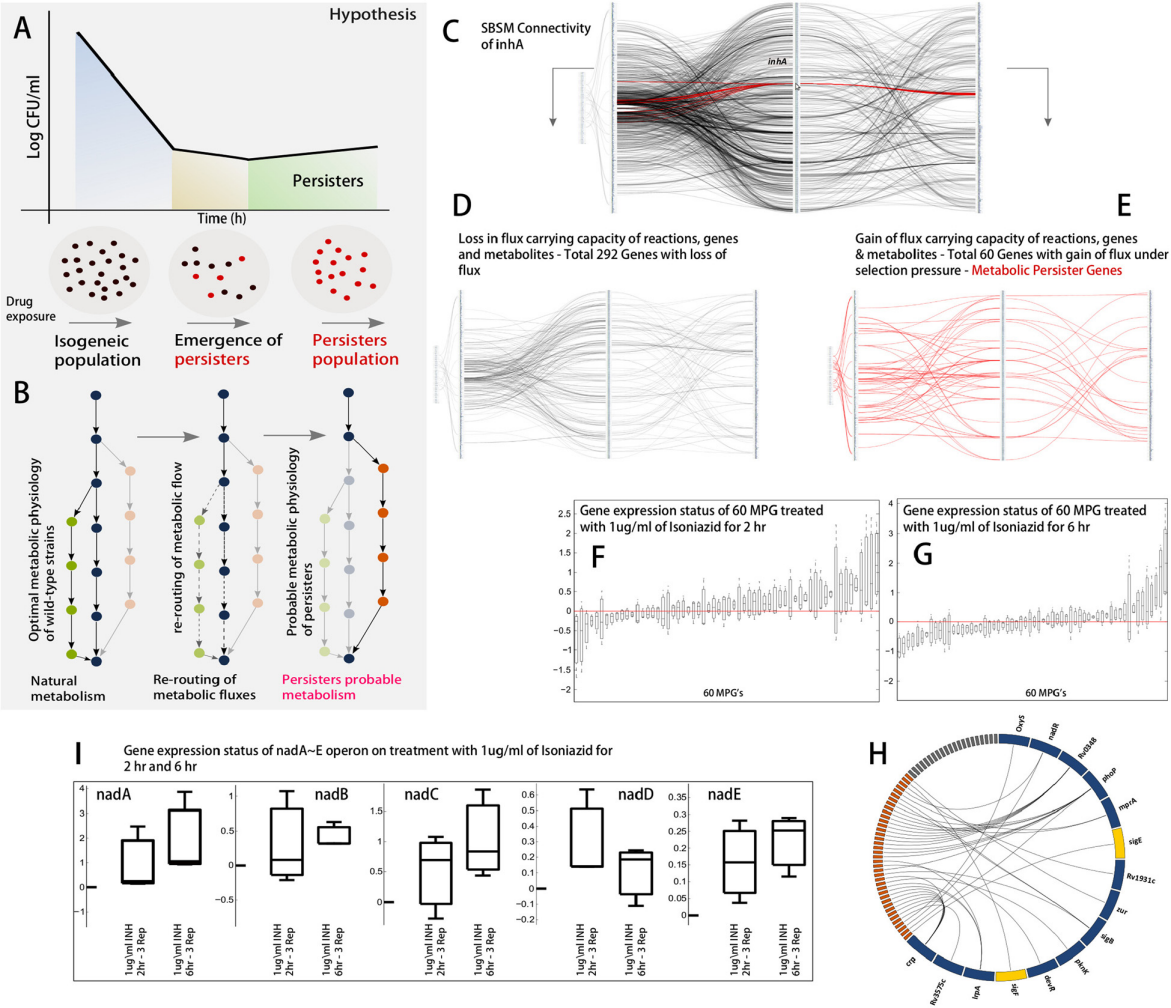
**Metabolic Persister Genes (MPGs) in the metabolism of** ***Mtb*** **A)** Bi-phasic killing and emergence of persisters upon drug exposure; **B)** Directional re-routing of metabolic fluxes resulting in the adaptation of the bacterium and the emergence of persiters; **C)** SBSM illustrating the metabolite and reaction connectivity to inhA, the target of Isoniazid; **D)** Loss of metabolic information in terms of metabolites, genes and reaction following inhA knock-out; **E)** Gain of function following inhA knock-out showing persister metabolites (PM), persister genes (MPGs) and persister reactions (PR); **F**-**G)** Gene expression status of 60 MPG’s on treatment with Isoniazid at 1 μg/ml for 2 hr and 6 hr in vivo **H)** Transcriptional control of 60 MPGs **I)** Expression status of *nadA* ~ *E* operon on treatment with Isoniazid at 1 μg/ml for 2 hr and 6 hr in vivo.

Consider a wild-type bacterium in an optimal metabolic steady state, as shown in (Figure 4B). As stress is introduced through antibiotics, the optimal metabolism can undergo changes in three ways such as a) *loss of function* (shutdown reactions that were active); b) *function regulation* (increase or decrease in the flux carrying capacity of active reactions); c) *gain of function* (activation of reactions that were dormant). The gain of function through the activation of reactions is responsible for the directional re-routing of metabolic fluxes, as illustrated in (Figure 4B). This results into alternative metabolic phenotypes. Over a prolonged period of antibiotic stress such phenotypes can eventually be selected, leading to the adaptation of the bacterium, which might demonstrate antibiotic tolerance.

We test this hypothesis by subjecting the metabolism of *Mtb* to sudden stress of known antibiotics such as Isoniazid (INH), Ethambutol (ETH), Rifampin (RIF) and the recently reported small molecule TCA1. Isoniazid is pro-drug which is activated by catalase-peroxidase hemoprotein, *katG*. It inhibits *inhA*, a nicotinamide adenine dinucleotide (NADH)-specific enoyl-acyl carrier protein (ACP) reductase involved in the fatty acid synthesis [49]. Ethambutol inhibits arabinosyltransferase which catalyzes the arabinogalactan and lipoarabinomannan polymer synthesis [49]. Arabinogalactan is a building block of the mucolyl-arabinogalactan-peptidoglycan layer that anchors it to the lipid-mycolic acid outer layer. Rifampin inhibits the β-subunit of the DNA-dependent RNA polymerase activity involved in transcription [49]. TCA1 is reported to inhibit DprE1, a component of decaprenyl-phosphoryl-β-D-ribofuranoseepimerase involved in cell wall synthesis [50]. The metabolic targets of INH (*embB*, *katG*, *inhA* and *kasA*) [51], the targets of ETH (*embA*, *manB* and *rmlD*) [51], the target of RIF (*embB*) [51] and the target of TCA1 (*DprE1*) [50] were identified from the literature.

In our analysis, each of these gene was knocked out individually and the flux carrying capacity of reactions were re-computed in order to identify the directional rerouting of metabolic fluxes (Methods). (Figure 4C) shows the metabolite and reaction connectivity of *inhA* in SBSM. The deletion of *inhA* resulted into a loss of flux carrying capacity of 328 reactions, which were subsequently mapped to 292 genes, (Figure 4D). However, a total of 57 reactions were found be involved in the directional re-routing of metabolic fluxes and were further mapped to their respective genes. A total of 60 enzyme-coding genes (hereafter referred as metabolic persister genes (MPGs) were identified that were responsible for catalyzing the reactions involved in the directional re-routing of metabolic fluxes (Figure 4E). Similarly all the genes (targets of antibiotics considered in this study) were knocked out one at a time and the resulting MPG’s were deciphered based on directional re-routing of metabolic fluxes (Additional file 1: Table–S10 and Additional file 4: Text-01).

### *in vitro and in vivo* assessment of MPGs based on experimental evidence

The MPGs were further analysed based on available experimental evidence from the literature both *in vitro* and *in vivo*. For isoniazid, the knock out of *inhA* as target resulted into 60 MPG’s. The global gene expression of these 60 MPG’s was further assessed based on a study reporting *Mtb* treatment with Isoniazid in a dormancy model attained through nutrient depletion and progressive hypoxia *in vitro* along with *in vivo* model of dormancy [52]. A total of 42 MPG’s of the total 60 were observed to be significantly up-regulated when treated with Isoniazid at 1 μg/ml for 2 hours (Figure 4F). The expression of these genes however was noted to reduce as a function of INH exposure (6 hr treatment, (Figure 4G). The high concordance between predicted MPG’s and their global up-regulation *in vivo* upon early exposure to Isoniazid suggest that the bacterium might use them as a resource to adapt its metabolism when challenged with the drug, resulting in an alternative metabolic phenotype. In addition, to probe the regulation of these MPGs, we mapped them to their respective transcription factors as illustrated in (Figure 4H). The transcription factors such as Rv0348 (*mosR*) and *crp* were observed to regulate most of the MPGs. Earlier research suggests that *mosR* controls the establishment of long term survival in the *Mtb* by inducing the *mce1* operon [40]. Bacterial cyclic-AMP receptor proteins (CRP) are a specific class of transcription factors that are suggested to induce upon activation of cAMP in *Mtb* [53]. In response, CRP binds to the target promoter regulating the expression of *repfA* and *whiB1*, subsequently leading to the emergence of persistence in *Mtb*. We also observed *sigF* and *sigE* regulating the two-MPGs. A two-fold up-regulation of *sigE* and *sigF* transcription factor has been measured in an isogenic population of *Mtb* when challenged with Isoniazid [54]. Among other transcription factors, we observed *oxyS* (Figure 4H) regulating one of the MPGs involved in maintaining intracellular H_2_O_2_ levels.

### Directional re-routing of metabolic fluxes as mechanism of adaptation

**Figure 5:**
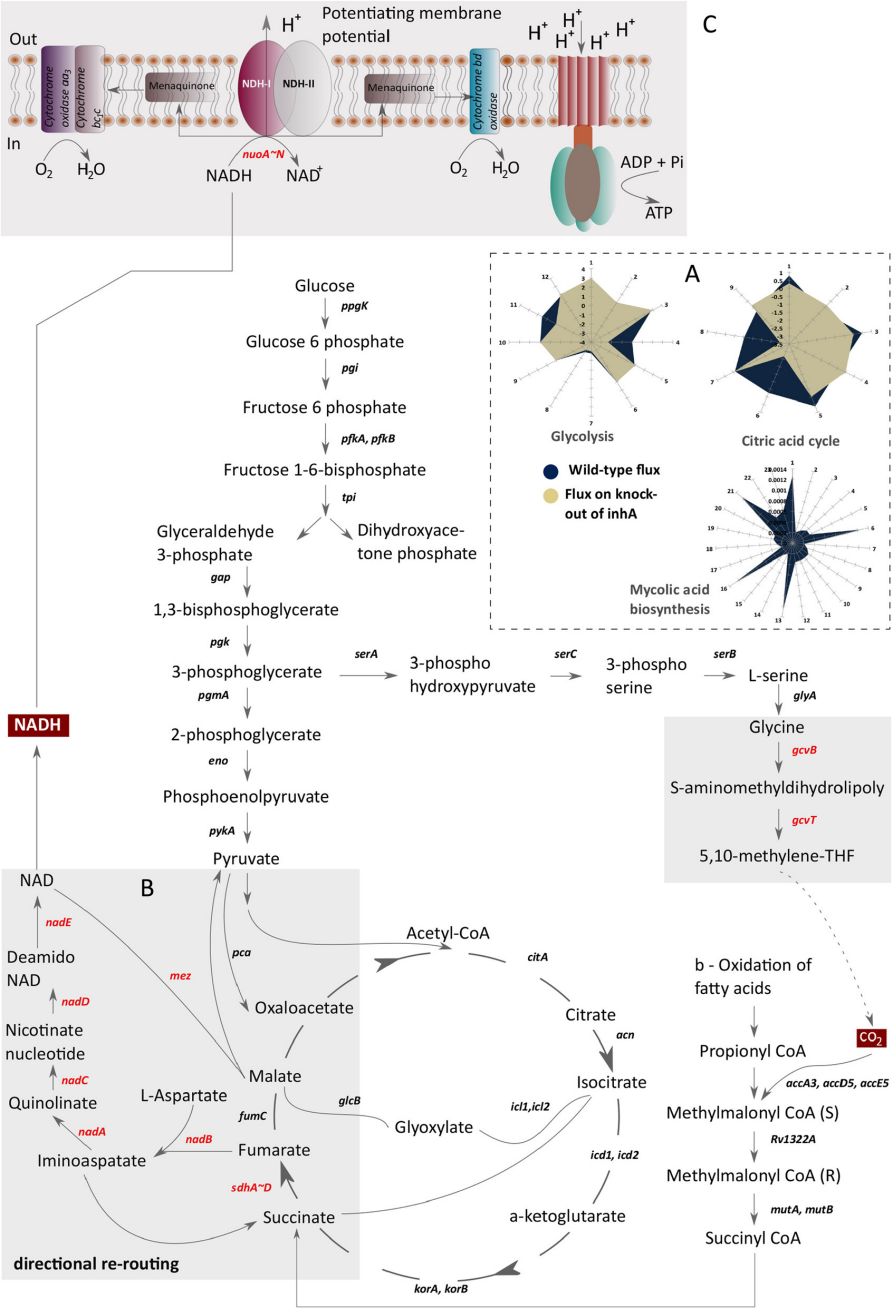
**Metabolic mechanism of adaptation. A)** Flux arrest in glycolysis, citric acid and mycolic acid pathway; **B)** Activation of NAD pathway *de novo* as computed based on directional re-routing of metabolic fluxes; **C)** Alternate mechanism of ATP generation based on proton motive force; *Mechanism*: *The activation of nadA* ~ *E operon lead to de novo biosynthesis of NADH*, *pool which is then reduced to NAD following the activation of the nuoA* ~ *Eoperon coding for NDH*-*I. This maintains the electron flow with proton translocation*, *which increases the potential difference across cell membrane*, *and can potentiate ATP production*, *thereby providing the necessary energy when challenged with antibiotic stress*.

Upon removal of targeted genes a significant reduction in the flux carrying capacity of glycolysis and citric acid cycle along with the pentose phosphate pathway (not shown) was observed as an overall response of metabolism as illustrated in (Figure 5A). The flow of metabolic fluxes through mycolic acid biosynthesis (primary target of Isoniazid) and the pentose phosphate pathway appeared to be completely shutdown as shown in (Figure 5A). The reduction in the fluxes of glycolysis and citric acid cycle is distant from mycolic acid biosynthesis, which is the direct target of isoniazid. This clearly shows that the drug exposure triggers systems level changes. A drastic reduction in both growth and central metabolic activity has been suggested as a predominant mechanism of *Mtb* adaptation under antibiotic stress [55].

We observe that the directional re-routing of metabolic flux via reactions catalysed by the *sdhA*-*D*, *nadA*-*E* and *nuoA*-*N* operons is the predominant response to all four antibiotics considered in this study. The activation of the *sdhA*-*D* operon increases the production of fumarate from succinate in the citric acid cycle as illustrated in (Figure 5B). The fumarate then combines with l-Aspartate and initiates the *de novo* biosynthesis of NAD^+^ cofactors, as observed through the activation of the *nadA*-*E* operon. The *nadA* ~ *E* operon was also observed to be completely unregulated upon treatment with Isoniazid at 1 μg/ml for 2 hr and 6 hr of Isoniazid exposure *in vivo* (Figure 4I) [52]. The NAD^+^ cofactors produced are then reduced to NADH pool in a reaction catalysed by *mez*, converting malate into pyruvate. The activation of *nuoA*-*N* further suggests the oxidation of NADH to NAD^+^ by NDH-I dehydrogenase. Studies have characterized two class of NDH in *Mtb*. NDH-I is a type one oxidoreductase in which the redox activity is coupled with the proton translocation, whereas NDH-II, is a type two oxidoreductase that performs electron transport without proton pumping across membrane. Further, cytochrome *bd* oxidase aa_3_ – type cytochrome *c* oxidase (CcO) has been shown to actively participate in the electron transport chain of *Mtb*. Based on the directional re-routing of metabolic fluxes we propose the following probable mechanism of *Mtb* adaption.

As most of citric acid cycle and pentose phosphate pathway components undergo flux arrest, the activation of the *nadA*-*E* operon increase the intracellular production of NAD^+^*de novo* (Figure 5B). The NAD^+^ is further reduced to NADH as observed by the activation of *mez*. The activation of the *nuoA*-*N* operon further suggests the oxidation of NADH through NDH-I dehydrogenase. The electron produced in this process is further transferred to either bd oxidase or CoC branch, and eventually to a terminal electron acceptor. The proton produced by NADH oxidation is however translocated across the cell membrane. This eventually might increases the potential difference across the cell membrane, subsequently leading to ATP production that is required for cell survival when it is under antibiotic stress (Figure 5C). We therefore suggest that *de novo* biosynthesis of the NAD^+^ cofactor is crucial for maintaining the viability of the cellular redox potential, which can eventually be harnessed for the production of ATP through the respiratory metabolism of *Mtb*. We support our finding with available experimental evidence from literature *in vitro* and *in vivo* [56,57] demonstrating the importance of *de novo* biosynthesis in the growth, adaptation, survival and persistence of *Mtb* in conditions similar to that of human infection.

### Spectrum of metabolic persister genes in *Mtb*

**Figure 6:**
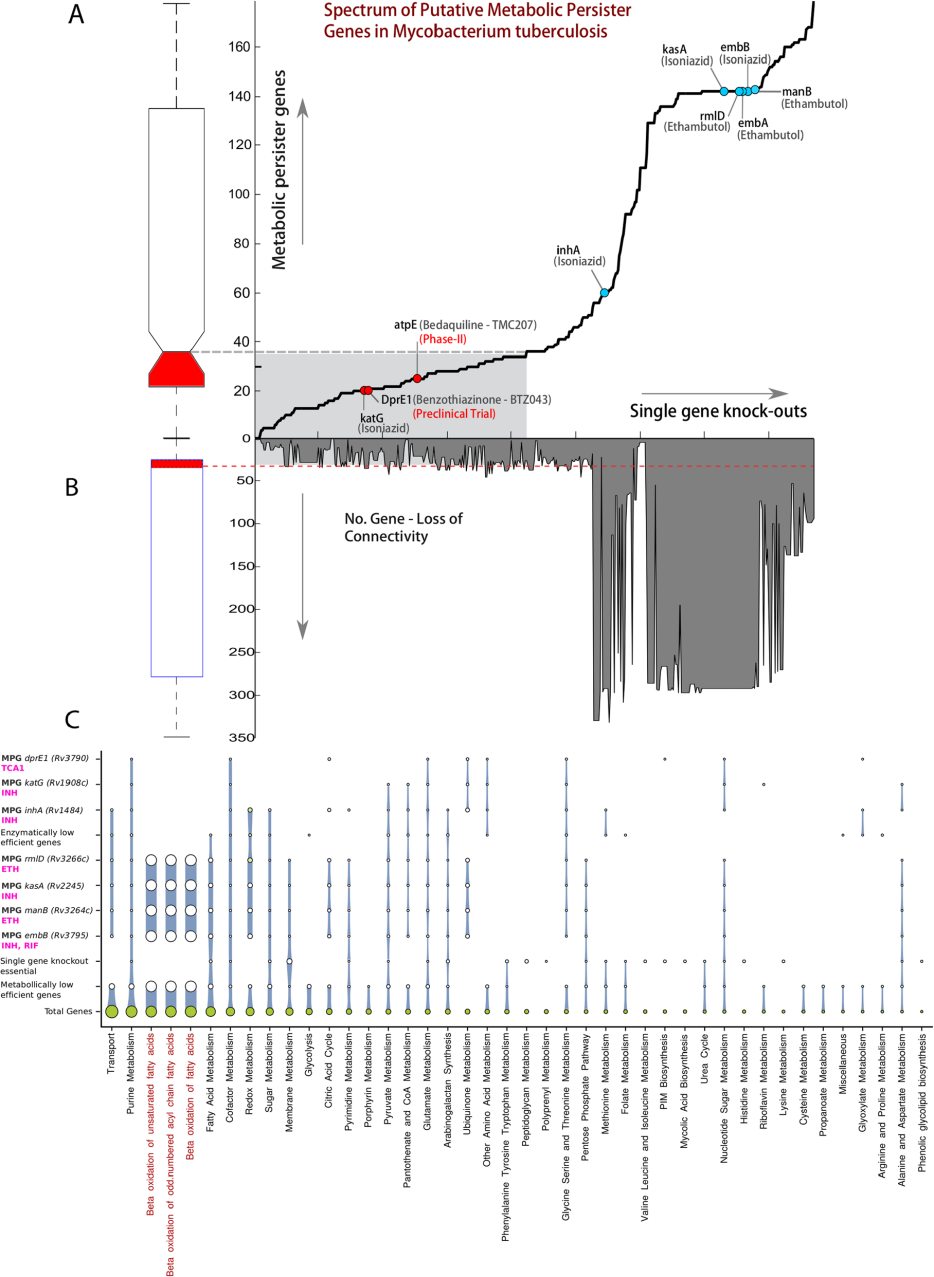
**Spectrum of metabolic persister genes in** ***Mtb*** **and pathway distribution of potential non-toxic drug targets. A)** Spectrum of metabolic persister genes in *Mtb*. X-axis represents the total number of gene knockouts. Total number of MPG for respective knockout is shown above x-Axis and total number of affected genes is shown below x-axis **(B)** For both A and B the respective box-plots are shown with the region below median colored red representing genes of interest. **C)** Pathway level coverage of essential genes: All the essential genes and the metabolic persisters genes are plotted relative to total number of genes in a given pathway.

Further, a spectrum of MPG’s in *Mtb* was formulated by considering all the metabolic genes as drug targets (Figure 6A). For every single gene knock out in the model, a total of 434 genes resulted into various MPG’s based on directional re-routing of metabolic fluxes (Additional file 1: Table S11). For any given knockout the range of MPG’s were observed to vary from 1 to 179, with median value of 36 (Figure 6A). A total of 211/434 genes were observed in the range below median value (Figure 6A, Additional file 1: Table S12). Predominantly these 211 genes belonged to *nuoA* ~ *L* operon, *sugA* ~ *C* operon, *frdA* ~ *D* operon *atpA* ~ *H* operon and *proV* ~ *X* operon. Off these operons, the *sugA* ~ *C* has been suggested to involved in carbohydrate transport to maintain *Mtb* virulence in the context of host-pathogen interaction [58], *atpA* ~ *H* codes of ATP-Synthase, which has been proposed as drug target earlier [59]. Except *katG* and *dprE1*, the MPG’s for all the targets (*inhA*, *kasA*, *embB*, *rmlD*, *embA and manB*) of other antibiotics considered in this study were observed to be higher than median of all MPGs’ (Figure 6A). The high value of MPG’s for these known antibiotics suggest a diversity in the options for bacteria to circumvent the impact of these antibiotics and hence likely to become drug tolerant. Contrary to this, the knock-out of genes with MPG’s below the median value can therefore be hypothesized as potential drug targets in combination with existing antibiotics as they are likely to get a limited support from the alternate metabolism and hence lower chances of developing drug tolerance. We support this hypothesis with the fact that the *katG*, *dprE1* and *atpE1* all having MPGs below the median value and are also approved drug targets for *Mtb*, which have cleared preclinical and phase-II trials successfully (www.tballiance.org).

Interestingly these drug targets also have lesser number of genes that were affected due to their knockout (Figure 6B). In the process of drug discovery it is essential to minimize the chances of drug toxicity, which can be achieved by minimizing the concentration of ligand and by maximizing its impact on the over all metabolic physiology. With this objective, we further mapped the intracellular abundance of proteins encoded by these 211 metabolic persister genes. A total of 112 off the 211 proteins were observed to have intracellular protein abundance lower than the average protein abundance of *Mtb* proteome [38], suggesting them further as potential drug targets for which ligands with minimum intracellular concentration could be designed.

The spectrum of metabolic persister genes in *Mtb* therefore for the first time suggest a new strategy that can be utilized to comprehend the impact of a given gene knockout by accounting for all possible mechanism through which such an impact could be buffered by the complexity of metabolism. We therefore propose these 211 genes as potential drug targets (of which one *katG* is known and *dprE1* and *atpE* have clear preclinical and phase-II trials), which can accelerate the drug discovery for drug tolerant *Mtb* and potentiate translation of various therapeutic interventions for the same.

### Potential non-toxic drug targets

Of the total 50 metabolic pathways, we observed essential genes in 42 pathways (Figure 6C). We have identified 33 of these 42 pathways that contain genes that are metabolically low efficient (MLE), 24 (out of 42) that are lethal, and 18 (out of 42) that are enzymatically low efficient (ELE) (Figure 6C). Interestingly most of the MPGs and MLE genes were observed in β-oxidation of fatty acids. This clearly suggests that *Mtb* utilized its β-oxidation pathway under stress condition despite harnessing its potential in wild type optimal metabolic physiology.

All the predicted essential genes were then compared with the human genome and human microbiome at the sequence level. The goal was to identify drug targets with the least likelihood of side effects. Of the total 116 lethal genes obtained from single gene knockout, 104 genes were found to be absent from the human genome, of which 48 were also found to have no homology in the human microbiome (Additional file 1: Table S5). For the 48 ELE essential genes, 32 were absent from the human genome, of which 26 had no homology to the human microbiome (Additional file 1: Table S6). For the 281 MLE essential genes, 207 were found to be absent from the human genome, of which 138 were also absent from the human microbiome (Additional file 1: Table S7). Few of the predicted targets with no sequence homology with human genome and microbiome are discussed below. The potential drug targets mostly belong to operons in *Mtb*.

*The PPS system*: the PPS system is made up of five genes. *ppsA* ~ *E* encodes for a type-I polyketide synthase. This operon codes for the protein responsible for the production of phthiocerols and phenolphthiocerols. Esterified by multimethyl-branched chain fatty acids, these complex lipids are composed of long chain β-diols, and are vital for maintaining the integrity of the cell wall [60]. In *M. bovis* BCG, *ppsA*-*E* has been shown to be responsible for the synthesis of phthiocerols and phenolphthiocerols through a mechanism involving the elongation of the C20-C22 fatty acyl chain containing a phenol moiety with three malonyl-CoA and two methylmalonyl CoA [61].

*The PIM system*: the PIM system is made up of three genes, *pimA* an α-mannosyltransferase, *pimB*, and *pimE*, a mannosyltransferase. Along with phosphatidylinositol (PI), the phosphatidylinositol mannosides (PIMs) are predominantly phospholipids synthesized metabolically in mycobacteria [62]. Anchoring two lipoglycans and lipomannan, the lipoarabinomannan is a crucial modulator of immune response during the course of a TB infection [63]. PIM synthesis begins with a mannose residue transfer from GDP-Man to the myo-inositol ring of PI resulting into the formation of phosphatidylinositol monomannosides subsequently resulting into the highly branched lipoglycans, lipomannan, and lipoarabinomannan with intermediate steps involving phosphate based sugar donors.

*The KDP system*: it codes for *kdpA*, *kdpB* and *kdpC*, and is a vital component of the host-pathogen interaction and for maintaining a K^+^gradiant across the *Mtb* cell membrane during the course of infection. As a mechanism of action, studies of the histidine kinase *kdpD* of E. coli suggests autophosphorylation and dephosphorylation of the response regulator *kdpE*, which in turn regulates the expression of the *kdpFABC* operon, acting a primary stress response to osmotic pressure by regulating the levels of K^+^. The change in osmotic pressure is sensed by the K^+^-transporting P-type ATPase enzyme, which subsequently results in the import and export of osmotic fluids such as K^+^ [64].

*The SDH system*: it codes for *sdhA*-*D*, succinate dehydrogenase, which plays a vital role in the adaptation of M. smegmatis to restricted energy availability. The exact role of Sdh enzymes along with their presence at different locations in the cell membrane of mycobacterium species is unclear [65]. Menaquinone has been suggested as an electron acceptor for Sdh, which would indicate a thermodynamically stable conversion of fumarate to succinate [65].

## Discussion

Almost one-third of worlds’ population is reported to harbor the latent form of TB. Therapeutic interventions to treat normal TB range from six months to a year, and up to 24 months in the case of MDR and XDR TB. The prolonged and sometimes improper use of multiple antibiotics has caused *Mtb* to become resistant to most of them. The emergence of persisters, adds another dimension to the problem. The severity of this situation and its devastating impact on public health have created an emergency situation in many countries that demands new approaches and interventions to develop new therapeutic interventions that can potentiate TB-translation research and reduce the global burden of disease.

In order to address the issues pertaining to the identification of potential non-toxic drug targets in *Mtb*, we have established and implemented an integrated data-intensive systems level framework for the analysis of its metabolism. The metabolism of *Mtb* was reconstructed manually in a bottom up manner by utilizing wealth of legacy data present as unstructured form of information in literature. Our reconstruction has significantly improved the metabolic knowledge of *Mtb* in contrast to its predecessors. The complex metabolic information was then represented by developing a novel Systems Biology Spindle Map (SBSM) that elucidate the inherent structure of metabolism in the context of genome organization and function. Further we identify various essential genes based on single-gene knock out experiments, the genes that code for enzymatically low efficient proteins and metabolically low efficient genes. We also identify various essential genes based on the modular nature of metabolic topology in *Mtb* and further map them to their respective transcription factors to identify potential drug targets form a polypharmacology approach. We assess the potential of predicted genes based on substantial amount of experimental evidence available both *in vitro* and *in vivo* experimental studies by referring to wealth of literature information in contrast to previous studies.

Further we formalized a novel concept of metabolic persister genes (MPGs) and identified a spectrum of such genes that can probably give rise to persister phenotypes of *Mtb* and hence may be responsible for drug tolerance. We have modeled antibiotic stress as selection pressure to investigate the directional re-routing of metabolic fluxes as a prime mechanism that allows *Mtb* to adapt to changing environmental conditions, and develop persister phenotypes. Pathways such as the β-oxidation of unsaturated fatty acids, the β-oxidation of acyle chain fatty acids and the β-oxidation of fatty acids were found to contain a large number of persister genes as shown in the response to Isoniazid and Ethambutol challenges (Figure 6C). We have specifically assessed *Mtb*’*s* metabolic response to three commonly used antibiotics along with a recently reported small molecule TCA1. We have observed the directional re-routing of metabolic fluxes through *de*-*novo* biosynthesis of NADH, which is then reduced by type-I NDH. This along with electron transfer and proton translocation might increase the potential difference across the membrane eventually leading to ATP production, critical for the survival under antibiotic stress. While earlier analysis has suggested *de novo* synthesis of NADH as a mechanism of survival under hypoxic conditions, the precise mechanism has been unclear, but is now explained to some extent by our results on the directional re-routing of *Mtb*'*s* metabolism. It has also been suggested that NDH-II but not NDH-I is important for survival under hypoxic conditions. Since NDH-I is involved in proton translocation, which can increase the potential difference across cell membrane, we reason that the directional re-routing of metabolic fluxes through *de novo* synthesis of NADH is a viable mechanism to explain persister formation. We support our hypothesis based on earlier [56] and a very recent investigation [57] suggesting the importance of *de novo* synthesis of NAD *in vivo* towards maintaining the viability and persistence of *Mtb* in conditions relevant to clinical infections.

While the activation of NDH-I can lead to increase in electron gradient across cell membrane and eventually leading to ATP production, it can also facilitate the production of reactive oxygen species (ROS) due to leakage in electron flow. In the light of recent investigations [66], we believe that our proposed mechanism can also increase ROS production and make existing antibiotics more effective when used in combination. However, the implication of such mechanism in ROS production remains uncertain and might be investigated experimentally in future work. Earlier investigations also suggest a metabolite mediated increase in uptake of aminoglycosides as a mechanism of persister eradication as a result of increased proton motive force [67]. While this mechanism is specific for aminoglycosides, it certainly remains to be tested for *Mtb* given the high rate of mutations in its drug efflux pumps [68].

We believe that the comprehensive data-intensive systems level analysis performed in this study can significantly potentiate the on going efforts of Tb drug discovery. In the context of build a systems-level understanding further, the model proposed by us in this analysis can be integrated with human macrophage [69]. iOSDD890 when combined with existing model of macrophage [69] is likely to improve the predictive power of host-pathogen model that can improve our basic understanding in an evolutionary context and rationalize the design of efficient and effective drug targets.

## Conclusion

Tuberculosis remains a leading cause of mortality across the globe. There is an urgent need for the development of new strategies and interventions to discover new drug targets or repurpose the existing ones. Towards this, the data intensive systems level analysis of metabolic complexity presented in this study can substantially aid in streamlining the ongoing efforts of TB drug discovery by identifying potential drug targets that are lethal for bacterial growth and can aid in the emergence of bacterial persistence. We believe that framework present in this study is generic and can be extended to study other neglected disease caused by various pathogenic prokaryotes.

## Additional files

Additional file 1: Table S1.
**(** **A** **-** **C** **)** – A) List of reactions, reaction description, formula, genes and subsystem of iOSDD890; B) List of metabolites, metabolite names and charged formula that of iOSDD890; C) List of genes and literature evidence of iOSDD890. **Table S2.** - List of metabolite-gene connectivity of SBSM in *Mtb*. **Table S3.** - Gene reaction connectivity of SBSM in *Mtb*. **Table S4.** - Complete list of model genes, active gene and the rpkm value of transcript of active gene. **Table S5.** - List of Single-Gene knock out essential genes and their literature based validation along with sequence level comparison with human genome and microbiome, the information on availability of PDB structure is also provided. **Table S6.** - List of ELE essential genes and their literature based validation along with sequence level comparison with human genome and microbiome, the information on availability of PDB structure is also provided. **Table S7.** - List of MLE essential genes and their literature based validation along with sequence level comparison with human genome and microbiome, the information on availability of PDB structure is also provided. **Table S8.** - Intracellular concentration of proteins encoded by single-gene knockout, ELE and MLE essential genes. **Table S9.** **(** **A** **-** **I** **)** - List of genes belonging to each module along with name, pathway mapping, literature evidence, sequence level comparison with human genome and microbiome along with the availability of PDB structure. **Table S10.** - List of all the MPG's and metabolite level mapping for each knock out target of all the antibiotics considered in this study. **Table S11.** - List of MPG’s for knockout of all the genes present in the model. **Table S12.** - List of 211 Potential genes. Additional file 2: Figure S1. Comparison of iOSDD890 with GSMN-TB, iNJ661 and iNJ661m at the level of metabolites, genes and reactions. Additional file 3:
**Model-01 iOSDD890 model in MATLAB compatible format.**
Additional file 4: Text-01. Detailed SBSM analysis for all the four antibiotics considered in this study.
