# Supplementary figures and images for: Systems level mapping of metabolic complexity in Mycobacterium tuberculosis to identify high-value drug targets
Source: J Transl Med. 2014 Oct 11;12:263. doi: 10.1186/s12967-014-0263-5 (PMC4201925; doi:10.1186/s12967-014-0263-5)

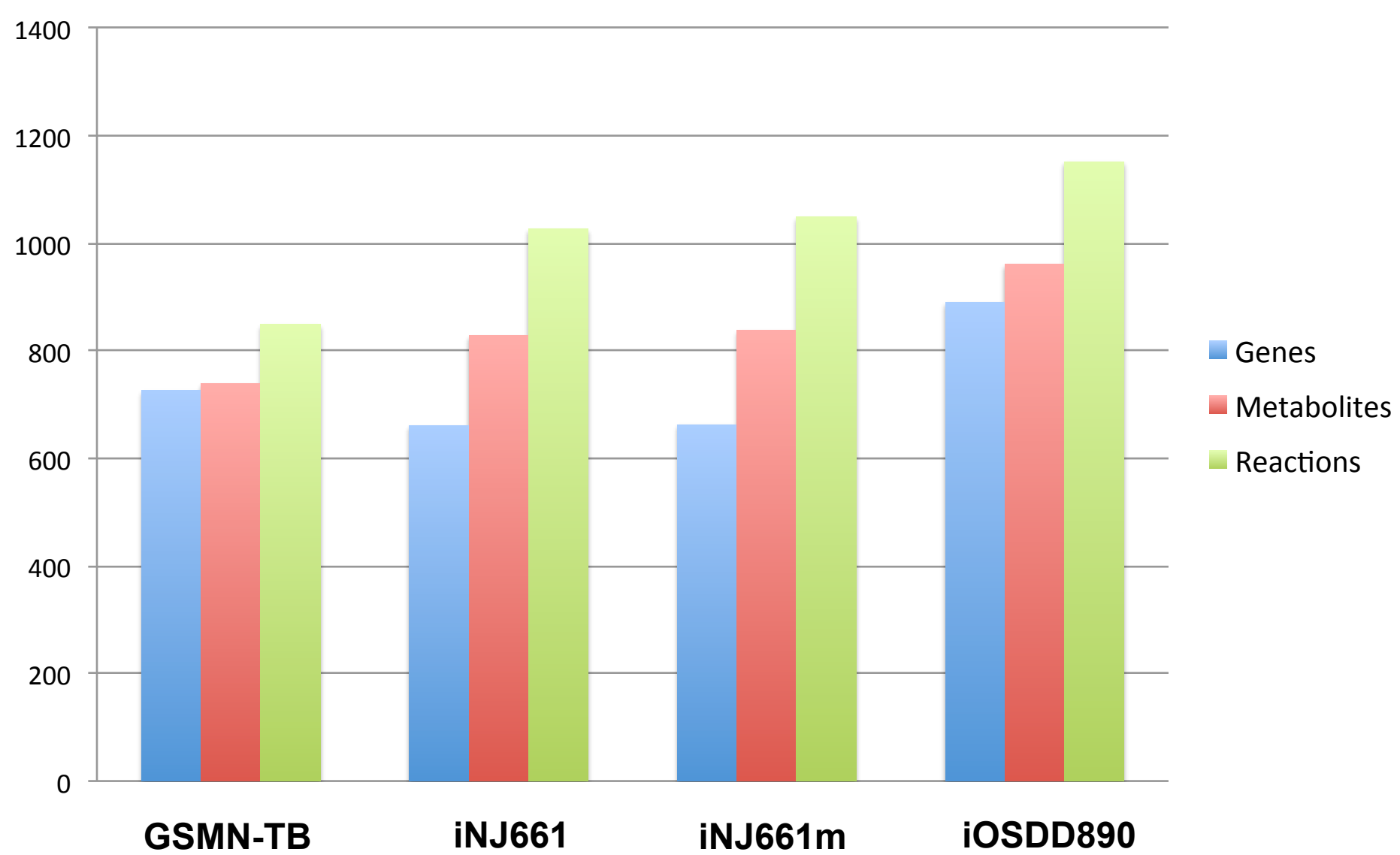

Supplement: Additional file 2: Figure S1. — Comparison of iOSDD890 with GSMN-TB, iNJ661 and iNJ661m at the level of metabolites, genes and reactions. [file 12967_2014_263_MOESM2_ESM.pdf]
