# Supplementary material for: Systems level mapping of metabolic complexity in Mycobacterium tuberculosis to identify high-value drug targets
Source: J Transl Med. 2014 Oct 11;12:263. doi: 10.1186/s12967-014-0263-5 (PMC4201925; doi:10.1186/s12967-014-0263-5)
Supplement: Additional file 4: Text-01. — Detailed SBSM analysis for all the four antibiotics considered in this study. [file 12967_2014_263_MOESM4_ESM.pdf]

Supplementary Information

Mapping the metabolic complexity of Mycobacterium tuberculosis to understand persister formation and identify high-value drug targets

Rohit Vashisht, Ashwini G Bhat, Shreeram Kushwaha, Anshu Bhardwaj, OSDD Consortium and Samir K Brahmachari*

Contents

1 Fig. S1 - Spindle Map Rv3795 3

2 Table - S15 Metabolic Persister Reactions, Genes and Pathway on Rv3795 Deletion 4

3 Fig. S2 - Spindle Map Rv1908c 16

4 Table - S16 Metabolic Persister Reactions, Genes and Pathway on Rv1908c Deletion 17

5 Table - S17 Metabolic Persister Reactions, Genes and Pathway on Rv1484 Deletion 19

6 Fig. S3 - Spindle Map Rv2245 23

7 Table - S18 Metabolic Persister Reactions, Genes and Pathway on Rv2245 Deletion 24

8 Fig. S4 - Spindle Map Rv3794 36

9 Table - S19 Metabolic Persister Reactions, Genes and Pathway on Rv3794 Deletion 37

10 Fig. S5 - Spindle Map Rv3264c 49

11 Table - S20 Metabolic Persister Reactions, Genes and Pathway on Rv3264c Deletion 50

12 Fig. S6 - Spindle Map Rv3266c 61

13 Table - S21 Metabolic Persister Reactions, Genes and Pathway on Rv3266c Deletion 62

14 Table - S22 Metabolic Persister Reactions, Genes and Pathway on Rv3790 Deletion 74

1 Fig. S1 - Spindle Map Rv3795

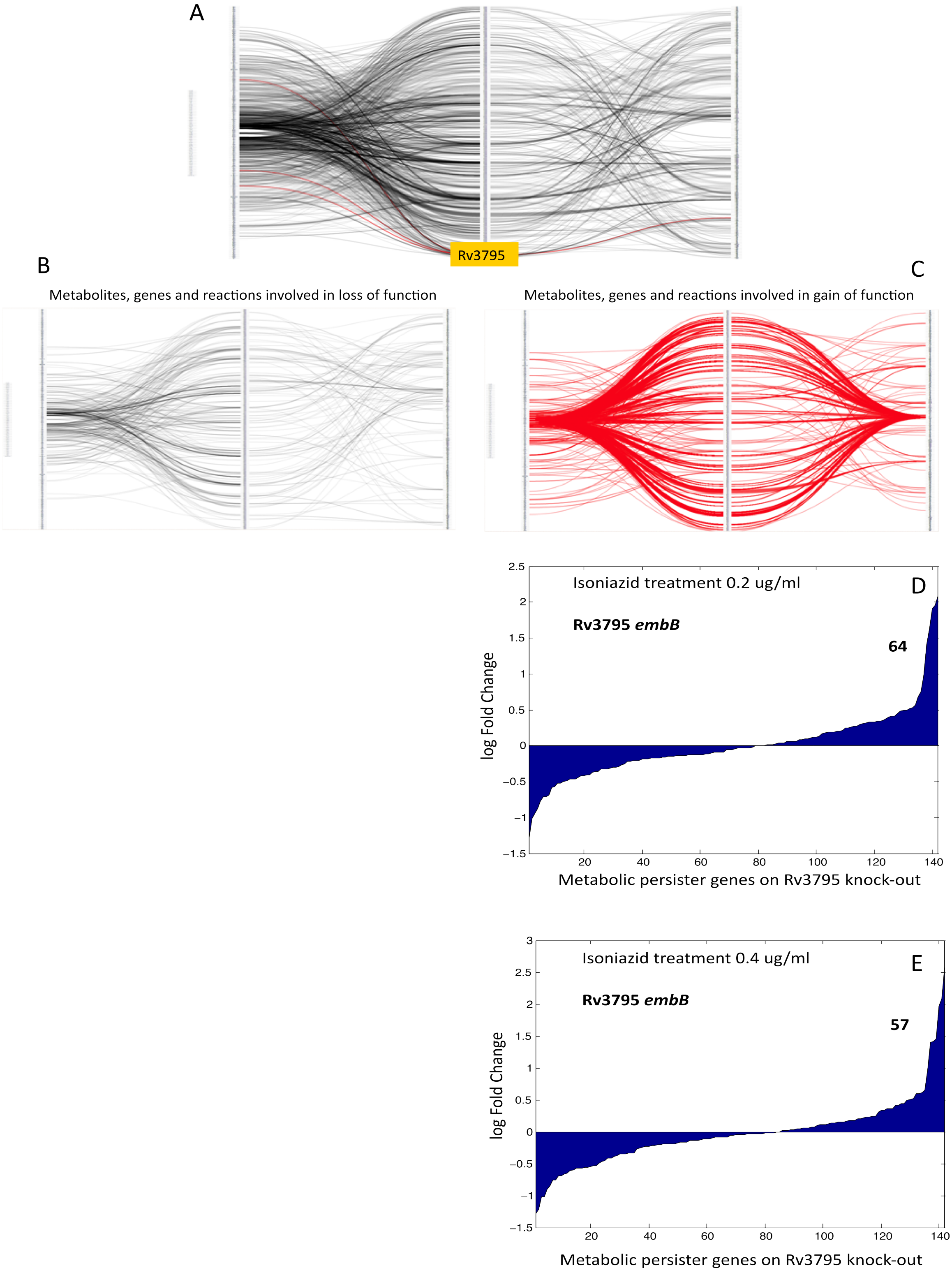

2 Table - S15 Metabolic Persister Reactions, Genes and Pathway on Rv3795 Deletion

Table 1: Metabolic persister reactions, gene and pathways on Rv3795 deletion

Reaction	Description	Formula	Genes	Pathways
ACKr	acetate kinase	ac[c] + atp[c] -> actp[c] + adp[c]	Rv0409	Pyruvate Metabolism
ADK1	adenylate kinase	amp[c] + atp[c] <=> 2 adp[c]	Rv0733	Purine Metabolism
ADK2	adenylate kinase Inorganic triphosphate	amp[c] + pppi[c] <=> adp[c] + ppi[c]	Rv0733	Purine Metabolism
ADK4	adentylate kinase ITP	amp[c] + itp[c] <=> adp[c] + idp[c]	Rv0733	Nucleotide Sugar Metabolism
ARGt5r	L arginine transport via diffusion	arg-L[e] <=> arg-L[c]	Rv2320c	Transport
ASPO5	L aspartate oxidase	asp-L[c] + fum[c] -> h[c] + iasp[c] + succ[c]	Rv1595	Alanine and Aspartate Metabolism
AST	Arginine succinyltransferase	arg-L[c] + succoa[c] -> coa[c] + h[c] + sucarg[c]		Arginine and Proline Metabolism
CAT	catalase	2 h2o2[c] -> 2 h2o[c] + o2[c]	Rv1908c	Redox Metabolism
CO2t	CO2 transporter via diffusion	co2[e] <=> co2[c]		Transport
CODH2r	carbon monoxide dehydrogenase acetyl CoA synthase 2	co[c] + 2 fdox[c] + h2o[c] <=> co2[c] + 2 fdred[c] + 2 h[c]	Rv0373c Rv0374c Rv0375c	Redox Metabolism
CODH3	carbon monoxide dehydrogenase acetyl CoA synthase 2	adfdOX[c] + 2 co[c] + 2 h2o[c] -> adfdRD[c] + 2 co2[c] + 2 h[c]	Rv0373c Rv0374c Rv0375c	Redox Metabolism
DCTPD2	dCTP deaminase	ctp[c] + h2o[c] + h[c] -> nh4[c] + utp[c]	Rv0321	Pyrimidine Metabolism
DHORD2	dihydoorotic acid dehydrogenase quinone8	dhor-S[c] + q8[c] -> orot[c] + q8h2[c]	Rv2139	Pyrimidine Metabolism
DHORD3	dihydroorotic acid dehydrogenase menaquinone 6	dhor-S[c] + mqn6[c] <=> mql6[c] + orot[c]	Rv2139	Pyrimidine Metabolism
ECHH-0	Ech hydrogenase no electron transport	2 fdox[c] + h2[c] <=> 2 fdred[c] + 2 h[c]		Miscellaneous

Continued on next page

Table 1 – continued from previous page

Reaction	Description	Formula	Genes	Pathways
ETHAt	ethanolamine transport via diffusion extracellular	etha[e] <=> etha[c]		Transport
EX-arg-L(e)	L Arginine exchange	arg-L[e] <=>		Exchange
EX-etha(e)	Ethanolamine exchange	etha[e] <=>		Exchange
EX-gly(e)	Glycine exchange	gly[e] <=>		Exchange
EX-k(e)	K exchange	k[e] <=>		Exchange
EX-mal-L(e)	L Malate exchange	mal-L[e] <=>		Exchange
EX-na1(e)	Sodium exchange	na1[e] <=>		Exchange
EX-ser-L(e)	L Serine exchange	ser-L[e] <=>		Exchange
FACOAL140	fatty acid CoA ligase tetradecanoate	atp[c] + coa[c] + ttdca[c] <=> amp[c] + ppi[c] + tdcoa[c]	Rv1529 Rv3826	Fatty Acid Metabolism
FACOAL161	fatty acid CoA ligase hexadecenoate	atp[c] + coa[c] + hdcea[c] <=> amp[c] + hdcoa[c] + ppi[c]	Rv1529 Rv3826	Fatty Acid Metabolism
FAO1	Fatty acid oxidation tetradecanoate	atp[c] + 7 coa[c] + 6 fad[c] + 6 h2o[c] + 6 nad[c] + ttdca[c] -> 7 accoa[c] + amp[c] + 6 fadh2[c] + 6 h[c] + 6 nadh[c] + ppi[c]	Rv0468 Rv0859 Rv0860 Rv1144 Rv1323	Fatty Acid Metabolism
FORMCOAL	formyl coa CoA ligase	atp[c] + coa[c] + for[c] <=> amp[c] + forcoa[c] + ppi[c]		Redox Metabolism
FRD	fumarate reductase	fadh2[c] + fum[c] -> fad[c] + succ[c]	Rv1552 Rv1553 Rv1554 Rv1555	Citric Acid Cycle
FRD2	fumarate reductase	fum[c] + mql8[c] -> mqn8[c] + succ[c]	Rv0247c Rv0248c Rv3316 Rv3317 Rv3318 Rv3319	Citric Acid Cycle
FRD5	fumarate reductase	fum[c] + mql6[c] <=> mqn6[c] + succ[c]	Rv1552 Rv1553 Rv1554 Rv1555	Citric Acid Cycle
G3PAT160	glycerol 3 phosphate acyltransferase C160	glyc3p[c] + palmACP[c] -> 1hdecg3p[c] + ACP[c]	Rv1551	Fatty Acid Metabolism
G6PDH2	glucose 6 phosphate dehydrogenase	g6p[c] + nadp[c] -> 6pgl[c] + 3 h[c] + nadph[c]	Rv1121 Rv1447c	Pentose Phosphate Pathway
GLYt2r	glycine reversible transport via proton symport	gly[e] + h[e] <=> gly[c] + h[c]	Rv1704c	Transport

Continued on next page

Table 1 – continued from previous page

Reaction	Description	Formula	Genes	Pathways
GPDDA2	Glycerophosphodiester phosphodiesterase Glycerophospho-ethanolamine	g3pe[c] + h2o[c] -> etha[c] + glyc3p[c] + h[c]	Rv0317c Rv2277c Rv3842c	Fatty Acid Metabolism
GTHOr	glutathione oxidoreductase	gthox[c] + h[c] + nadph[c] <=> 2 gthrd[c] + nadp[c]	Rv2855	Glutamate Metabolism
GTPDPK	GTP diphosphokinase	atp[c] + gtp[c] -> amp[c] + gdptp[c] + h[c]	Rv2583c	Purine Metabolism
HSDy	homoserine dehydrogenase NADPH	hom-L[c] + nadp[c] <=> aspsa[c] + h[c] + nadph[c]	Rv1294	Glycine Serine and Threonine Metabolism
Kt3r	potassium proton antiporter	h[e] + k[c] <=> h[c] + k[e]	Rv2691 Rv2692	Transport
LPLIPAL2E160	Lysophospholipase L2 2 acylglycerophospho-ethanolamine n C160	2agpe160[c] + h2o[c] -> g3pe[c] + h[c] + hdca[c]	Rv0183	Fatty Acid Metabolism
L-LACD2	L Lactate dehydrogenase ubiquinone	lac-L[c] + q8[c] -> pyr[c] + q8h2[c]	Rv1872c	Pyruvate Metabolism
MALt2r	L malate reversible transport via proton symport	h[e] + mal-L[e] <=> h[c] + mal-L[c]	Rv2443	Transport
ME1	malic enzyme NAD	mal-L[c] + nad[c] -> co2[c] + nadh[c] + pyr[c]	Rv2332	Pyruvate Metabolism
MYCTR	mycothiol reductase nadp dependent	2 msh[c] + nadp[c] <=> h[c] + mssg[c] + nadph[c]	Rv2855	Redox Metabolism
MYCTR2	mycothiol reductase glutathione exchange	gthox[c] + 2 msh[c] <=> 2 gthrd[c] + mssg[c]	Rv2855	Redox Metabolism
NADH2r	NADH dehydrogenase	h[c] + nadh[c] + q[c] <=> nad[c] + qh2[c]	Rv0082 Rv3145 Rv3146 Rv3147 Rv3148 Rv3149 Rv3150 Rv3151 Rv3152 Rv3153 Rv3154 Rv3155 Rv3156 Rv3157 Rv3158	Redox Metabolism
NADS2	NAD synthase glutamine hydrolysing	atp[c] + dnad[c] + gln-L[c] + h2o[c] -> amp[c] + glu-L[c] + h[c] + nad[c] + ppi[c]	Rv2438c	Glutamate Metabolism

Continued on next page

Table 1 – continued from previous page

Reaction	Description	Formula	Genes	Pathways
NAKtr	potassium sodium antiporter	k[c] + na1[e] <=> k[e] + na1[c]	Rv3236c	Transport
NAt3-1	sodium proton antiporter HNA is 11	h[e] + na1[c] <=> h[c] + na1[e]	Rv2287	Transport
NDPK9	nucleoside diphosphate kinase ATPIDP	atp[c] + idp[c] <=> adp[c] + itp[c]	Rv2445c	Pyrimidine Metabolism
NNAT	nicotinate nucleotide adenylyltransferase	atp[c] + h[c] + nicrnt[c] -> dnad[c] + ppi[c]	Rv2421c	Pantothenate and CoA Metabolism
NNDPR	nicotinate nucleotide diphosphorylase carboxylating	2 h[c] + prpp[c] + quln[c] -> co2[c] + nicrnt[c] + ppi[c]	Rv1596	Pantothenate and CoA Metabolism
ORNt	ornithine transport via diffusion extracellular to periplasm	orn[e] <=> orn[c]	Rv2320c	Transport
OXGDC	2 oxoglutarate decarboxylase	akg[c] + h[c] -> co2[c] + sucsal[c]	Rv1248c	Citric Acid Cycle
PGL	6 phosphogluconolactonase	6pgl[c] + h2o[c] -> 6pgc[c] + h[c]	Rv1445c	Pentose Phosphate Pathway
PHTO	phthiocol oxygenase	2 o2[c] + phthclh2[c] -> 2 h[c] + 2 o2s[c] + phthcl[c]		Ubiquinone Metabolism
PLIPA1E160	Phospholipase A1 phosphatidylethanolamine n C160	h2o[c] + pe160[c] -> 2agpe160[c] + h[c] + hdca[c]		Fatty Acid Metabolism
PPDK	Pyruvate phosphate dikinase	atp[c] + pi[c] + pyr[c] -> amp[c] + h[c] + pep[c] + ppi[c]	Rv1127c	Pyruvate Metabolism
PPK2	polyphosphate kinase	atp[c] + ppi[c] -> adp[c] + pppi[c]	Rv2984	Purine Metabolism
PTAr	phosphotransacetylase	accoa[c] + pi[c] <=> actp[c] + coa[c]	Rv0408 Rv3914	Pyruvate Metabolism
QRr	Quinone reductase	h[c] + nadph[c] + q[c] <=> nadp[c] + qh2[c]	Rv3777	Redox Metabolism
QULNS	quinolinate synthase	dhap[c] + iasp[c] -> 2 h2o[c] + pi[c] + quln[c]	Rv1594	Pantothenate and CoA Metabolism
SERt2r	L serine reversible transport via proton symport	h[e] + ser-L[e] <=> h[c] + ser-L[c]	Rv1704c	Transport
SGDS	Succinylglutamate desuccinylase	h2o[c] + sucglu[c] -> glu-L[c] + succ[c]		Arginine and Proline Metabolism

Continued on next page

Table 1 – continued from previous page

Reaction	Description	Formula	Genes	Pathways
SGSAD	Succinylglutamic semialdehyde dehydrogenase	h2o[c] + nad[c] + sucgsa[c] -> 2 h[c] + nadh[c] + sucglu[c]		Arginine and Proline Metabolism
SOTA	Succinylornithine transaminase	akg[c] + sucorn[c] -> glu-L[c] + sucgsa[c]		Arginine and Proline Metabolism
SPODM	superoxide dismutase	2 h[c] + 2 o2s[c] -> h2o2[c] + o2[c]	Rv0432 Rv1908c Rv3846	Cofactor Metabolism
SSALy	succinate semialdehyde dehydrogenase NADP	h2o[c] + nadp[c] + sucsal[c] -> 2 h[c] + nadph[c] + succ[c]	Rv0234c Rv1731	Glutamate Metabolism
XYLK	xylulokinase	atp[c] + xylu-D[c] -> adp[c] + h[c] + xu5p-D[c]	Rv0729	Sugar Metabolism
PPGPP	PPGPP-3-pyrophosphohydrolase	gdpdp[c] + h2o[c] -> gdp[c] + ppi[c]		Purine Metabolism

Continued on next page

Table 1 – continued from previous page

Reaction	Description	Formula	Genes	Pathways
FAD-8	acyl-CoA dehydrogenase fadE1-36 AND enoyl-CoA hydratase/isomerase echA1-21 AND 3-hydroxyacyl-CoA dehydrogenase fadB2-5 AND acetyl-CoA C-acetyltransferase fadA2-13	tdcoa[c] + fad[c] + nad[c] + coa[c] -> dodcoa[c] + fadh2[c] + nadh[c] + accoa[c]	Rv0131c Rv0154c Rv0215c Rv0231 Rv0244c Rv0271c Rv0400c Rv0752c Rv0873 Rv0972c Rv0975c Rv1346 Rv1467c Rv1679 Rv1933c Rv1934c Rv2724c Rv2789c Rv3061c Rv3139 Rv3140 Rv3274c Rv3504 Rv3505 Rv3543c Rv3544c Rv3560c Rv3562 Rv3563 Rv3564 Rv3573c Rv3761c Rv3797 Rv0672 Rv2500c Rv0222 Rv0456c Rv0632c Rv0673 Rv0675 Rv0971c Rv1070c Rv1071c Rv1141c Rv1142c Rv1472 Rv1935c Rv2486 Rv2679 Rv2831 Rv3039c Rv3516 Rv3550 Rv3774 Rv0905 Rv3374 Rv3373 Rv0468 Rv1715 Rv1912c Rv3141 Rv0243 Rv1074c Rv3546 Rv3556c Rv1323 Rv0859	Beta oxidation of fatty acids

Continued on next page

Table 1 – continued from previous page

Reaction	Description	Formula	Genes	Pathways
FAD-9	acyl-CoA dehydrogenase fadE1-36 AND enoyl-CoA hydratase/isomerase echA1-21 AND 3-hydroxyacyl-CoA dehydrogenase fadB2-5 AND acetyl-CoA C-acetyltransferase fadA2-14	dodcoa[c] + fad[c] + nad[c] + coa[c] -> decoa[c] + fadh2[c] + nadh[c] + accoa[c]	Rv0131c Rv0154c Rv0215c Rv0231 Rv0244c Rv0271c Rv0400c Rv0752c Rv0873 Rv0972c Rv0975c Rv1346 Rv1467c Rv1679 Rv1933c Rv1934c Rv2724c Rv2789c Rv3061c Rv3139 Rv3140 Rv3274c Rv3504 Rv3505 Rv3543c Rv3544c Rv3560c Rv3562 Rv3563 Rv3564 Rv3573c Rv3761c Rv3797 Rv0672 Rv2500c Rv0222 Rv0456c Rv0632c Rv0673 Rv0675 Rv0971c Rv1070c Rv1071c Rv1141c Rv1142c Rv1472 Rv1935c Rv2486 Rv2679 Rv2831 Rv3039c Rv3516 Rv3550 Rv3774 Rv0905 Rv3374 Rv3373 Rv0468 Rv1715 Rv1912c Rv3141 Rv0243 Rv1074c Rv3546 Rv3556c Rv1323 Rv0859	Beta oxidation of fatty acids

Continued on next page

Table 1 – continued from previous page

Reaction	Description	Formula	Genes	Pathways
FAD-11	acyl-CoA dehydrogenase fadE1-36 AND enoyl-CoA hydratase/isomerase echA1-21 AND 3-hydroxyacyl-CoA dehydrogenase fadB2-5 AND acetyl-CoA C-acetyltransferase fadA2-16	occoa[c] + fad[c] + nad[c] + coa[c] -> hexcoa[c] + fadh2[c] + nadh[c] + accoa[c]	Rv0131c Rv0154c Rv0215c Rv0231 Rv0244c Rv0271c Rv0400c Rv0752c Rv0873 Rv0972c Rv0975c Rv1346 Rv1467c Rv1679 Rv1933c Rv1934c Rv2724c Rv2789c Rv3061c Rv3139 Rv3140 Rv3274c Rv3504 Rv3505 Rv3543c Rv3544c Rv3560c Rv3562 Rv3563 Rv3564 Rv3573c Rv3761c Rv3797 Rv0672 Rv2500c Rv0222 Rv0456c Rv0632c Rv0673 Rv0675 Rv0971c Rv1070c Rv1071c Rv1141c Rv1142c Rv1472 Rv1935c Rv2486 Rv2679 Rv2831 Rv3039c Rv3516 Rv3550 Rv3774 Rv0905 Rv3374 Rv3373 Rv0468 Rv1715 Rv1912c Rv3141 Rv0243 Rv1074c Rv3546 Rv3556c Rv1323 Rv0859	Beta oxidation of fatty acids

Continued on next page

Table 1 – continued from previous page

Reaction	Description	Formula	Genes	Pathways
FAD-15	acyl-CoA dehydrogenase fadE1-36 AND enoyl-CoA hydratase/isomerase echA1-21 AND 3-hydroxyacyl-CoA dehydrogenase fadB2-5 AND acetyl-CoA C-acetyltransferase fadA2-20	hdcoa[c] + fad[c] + nad[c] + coa[c] -> c5tetdcoa[c] + fadh2[c] + nadh[c] + accoa[c]	Rv0131c Rv0154c Rv0215c Rv0231 Rv0244c Rv0271c Rv0400c Rv0752c Rv0873 Rv0972c Rv0975c Rv1346 Rv1467c Rv1679 Rv1933c Rv1934c Rv2724c Rv2789c Rv3061c Rv3139 Rv3140 Rv3274c Rv3504 Rv3505 Rv3543c Rv3544c Rv3560c Rv3562 Rv3563 Rv3564 Rv3573c Rv3761c Rv3797 Rv0672 Rv2500c Rv0222 Rv0456c Rv0632c Rv0673 Rv0675 Rv0971c Rv1070c Rv1071c Rv1141c Rv1142c Rv1472 Rv1935c Rv2486 Rv2679 Rv2831 Rv3039c Rv3516 Rv3550 Rv3774 Rv0905 Rv3374 Rv3373 Rv0468 Rv1715 Rv1912c Rv3141 Rv0243 Rv1074c Rv3546 Rv3556c Rv1323 Rv0859	Beta oxidation of unsaturated fatty acids

Continued on next page

Table 1 – continued from previous page

Reaction	Description	Formula	Genes	Pathways
FAD-16	acyl-CoA dehydrogenase fadE1-36 AND enoyl-CoA hydratase/isomerase echA1-21 AND 3-hydroxyacyl-CoA dehydrogenase fadB2-5 AND acetyl-CoA C-acetyltransferase fadA2-21	c5tetdcoa[c] + fad[c] + nad[c] + coa[c] -> c3dodcoa[c] + fadh2[c] + nadh[c] + accoa[c]	Rv0131c Rv0154c Rv0215c Rv0231 Rv0244c Rv0271c Rv0400c Rv0752c Rv0873 Rv0972c Rv0975c Rv1346 Rv1467c Rv1679 Rv1933c Rv1934c Rv2724c Rv2789c Rv3061c Rv3139 Rv3140 Rv3274c Rv3504 Rv3505 Rv3543c Rv3544c Rv3560c Rv3562 Rv3563 Rv3564 Rv3573c Rv3761c Rv3797 Rv0672 Rv2500c Rv0222 Rv0456c Rv0632c Rv0673 Rv0675 Rv0971c Rv1070c Rv1071c Rv1141c Rv1142c Rv1472 Rv1935c Rv2486 Rv2679 Rv2831 Rv3039c Rv3516 Rv3550 Rv3774 Rv0905 Rv3374 Rv3373 Rv0468 Rv1715 Rv1912c Rv3141 Rv0243 Rv1074c Rv3546 Rv3556c Rv1323 Rv0859	Beta oxidation of unsaturated fatty acids

Continued on next page

Table 1 – continued from previous page

Reaction	Description	Formula	Genes	Pathways
FAD-17	acyl-CoA dehydrogenase fadE1-36 AND enoyl-CoA hydratase/isomerase echA1-21 AND 3-hydroxyacyl-CoA dehydrogenase fadB2-5 AND acetyl-CoA C-acetyltransferase fadA2-22	c3dodcoa[c] -> t2dodcoa[c]	Rv0131c Rv0154c Rv0215c Rv0231 Rv0244c Rv0271c Rv0400c Rv0752c Rv0873 Rv0972c Rv0975c Rv1346 Rv1467c Rv1679 Rv1933c Rv1934c Rv2724c Rv2789c Rv3061c Rv3139 Rv3140 Rv3274c Rv3504 Rv3505 Rv3543c Rv3544c Rv3560c Rv3562 Rv3563 Rv3564 Rv3573c Rv3761c Rv3797 Rv0672 Rv2500c Rv0222 Rv0456c Rv0632c Rv0673 Rv0675 Rv0971c Rv1070c Rv1071c Rv1141c Rv1142c Rv1472 Rv1935c Rv2486 Rv2679 Rv2831 Rv3039c Rv3516 Rv3550 Rv3774 Rv0905 Rv3374 Rv3373 Rv0468 Rv1715 Rv1912c Rv3141 Rv0243 Rv1074c Rv3546 Rv3556c Rv1323 Rv0859	Beta oxidation of unsaturated fatty acids

Continued on next page

Table 1 – continued from previous page

Reaction	Description	Formula	Genes	Pathways
FAD-19	acyl-CoA dehydrogenase fadE1-36 AND enoyl-CoA hydratase/isomerase echA1-21 AND 3-hydroxyacyl-CoA dehydrogenase fadB2-5 AND acetyl-CoA C-acetyltransferase fadA2-24	d2hdodcoa[c] + nad[c] -> dodcoa[c] + nadh[c]	Rv0131c Rv0154c Rv0215c Rv0231 Rv0244c Rv0271c Rv0400c Rv0752c Rv0873 Rv0972c Rv0975c Rv1346 Rv1467c Rv1679 Rv1933c Rv1934c Rv2724c Rv2789c Rv3061c Rv3139 Rv3140 Rv3274c Rv3504 Rv3505 Rv3543c Rv3544c Rv3560c Rv3562 Rv3563 Rv3564 Rv3573c Rv3761c Rv3797 Rv0672 Rv2500c Rv0222 Rv0456c Rv0632c Rv0673 Rv0675 Rv0971c Rv1070c Rv1071c Rv1141c Rv1142c Rv1472 Rv1935c Rv2486 Rv2679 Rv2831 Rv3039c Rv3516 Rv3550 Rv3774 Rv0905 Rv3374 Rv3373 Rv0468 Rv1715 Rv1912c Rv3141 Rv0243 Rv1074c Rv3546 Rv3556c Rv1323 Rv0859	Beta oxidation of unsaturated fatty acids
ALDR	Aldolase reductase	arab-D[c] + nadph[c] + h[c] -> arabl-D[c] + nadp[c]		Arabinogalactan Synthesis
AL4D	D-arabinitol 4-dehydrogenase	arabl-D[c] + nad[c] -> xylu-D[c] + nadh[c] + h[c]	Rv1928c	Arabinogalactan Synthesis

3 Fig. S2 - Spindle Map Rv1908c

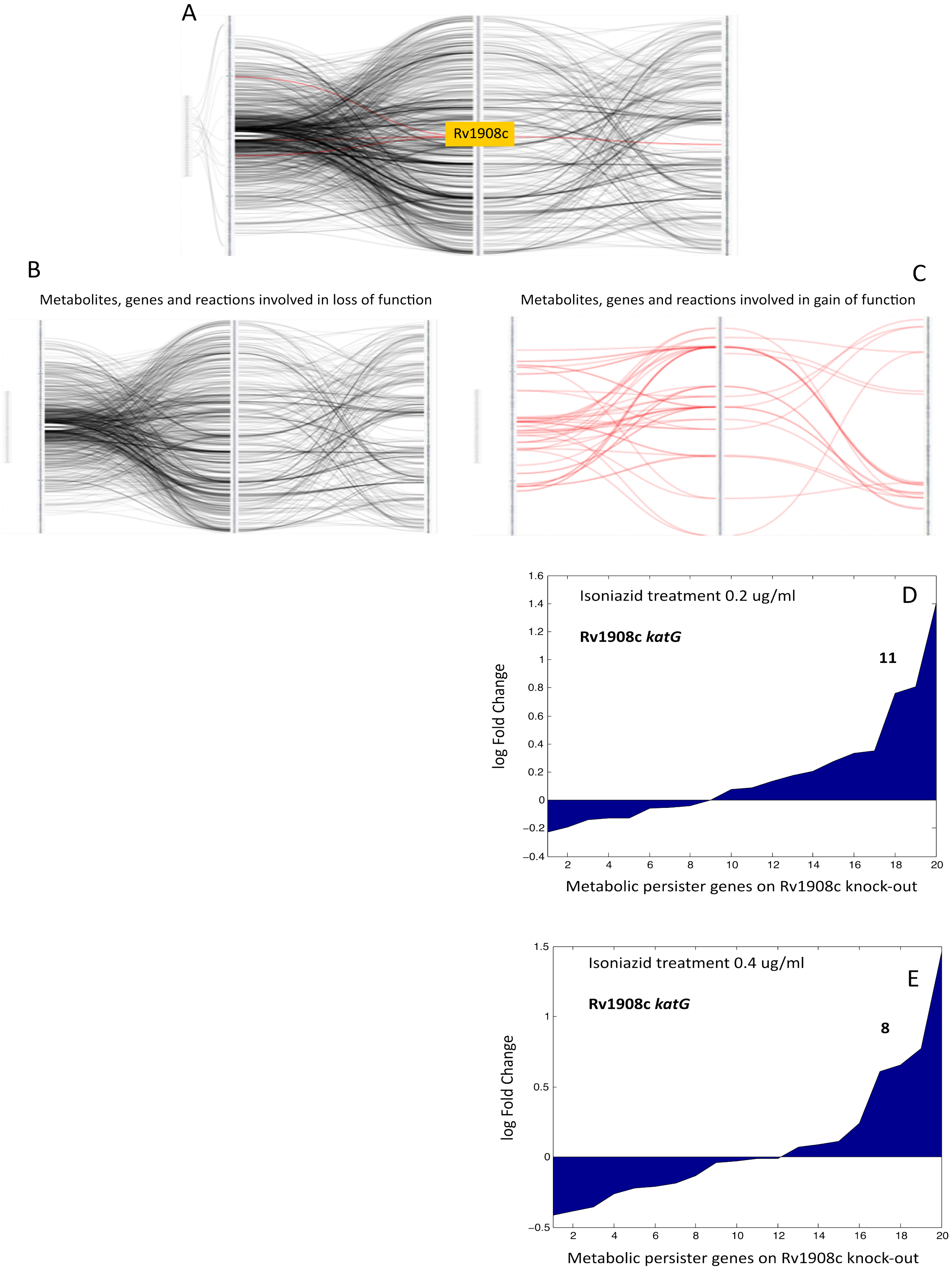

4 Table - S16 Metabolic Persister Reactions, Genes and Pathway on Rv1908c Deletion

Table 2: Metabolic persister reactions, gene and pathways on Rv1908c deletion

Reaction	Description	Formula	Genes	Pathways
ADK1	adenylate kinase	amp[c] + atp[c] <=> 2 adp[c]	Rv0733	Purine Metabolism
AMMQT8-2	S adenosylmethione2 demethylmenaquinone methyltransferase	2dmmq8[c] + amet[c] -> ahcys[c] + h[c] + mqn8[c]		Ubiquinone Metabolism
ASPO5	L aspartate oxidase	asp-L[c] + fum[c] -> h[c] + iasp[c] + succ[c]	Rv1595	Alanine and Aspartate Metabolism
DHNAOT	1 4 dihydroxy 2 naphthoate octaprenyltransferase	dhna[c] + octdp[c] -> 2dmmq8[c] + co2[c] + h[c] + ppi[c]	Rv0534c	Ubiquinone Metabolism
EX-cit(e)	Citrate exchange	cit[e] <=>		Exchange
GTPCII	GTP cyclohydrolase II	gtp[c] + 3 h2o[c] -> 25dhpp[c] + for[c] + 2 h[c] + ppi[c]	Rv1415 Rv1940	Riboflavin Metabolism
HSK	homoserine kinase	atp[c] + hom-L[c] -> adp[c] + h[c] + phom[c]	Rv1296	Glycine Serine and Threonine Metabolism
ICHORSi	Isochorismate Synthase	chor[c] -> ichor[c]	Rv3215	Ubiquinone Metabolism
NADS2	NAD synthase glutamine hydrolysing	atp[c] + dnad[c] + gln-L[c] + h2o[c] -> amp[c] + glu-L[c] + h[c] + nad[c] + ppi[c]	Rv2438c	Glutamate Metabolism
NNAT	nicotinate nucleotide adenylyltransferase	atp[c] + h[c] + nicrnt[c] -> dnad[c] + ppi[c]	Rv2421c	Pantothenate and CoA Metabolism
NNDPR	nicotinate nucleotide diphosphorylase carboxylating	2 h[c] + prpp[c] + quln[c] -> co2[c] + nicrnt[c] + ppi[c]	Rv1596	Pantothenate and CoA Metabolism
NPHS	naphthoate synthase	sbzcoa[c] -> coa[c] + dhna[c]	Rv0548c	Ubiquinone Metabolism
NTD11	5 nucleotidase IMP	h2o[c] + imp[c] -> ins[c] + pi[c]		Nucleotide Sugar Metabolism
PROt2r	L proline reversible transport via proton symport	h[e] + pro-L[e] <=> h[c] + pro-L[c]		Transport

Continued on next page

Table 2 – continued from previous page

Reaction	Description	Formula	Genes	Pathways
PTAr	phosphotransacetylase	accoa[c] + pi[c] <=> actp[c] + coa[c]	Rv0408 Rv3914	Pyruvate Metabolism
QULNS	quinolinate synthase	dhap[c] + iasp[c] -> 2 h2o[c] + pi[c] + quln[c]	Rv1594	Pantothenate and CoA Metabolism
SHCHCS	2 succinyl 6 hydroxy 2 4 cyclohexadiene 1 carboxylate synthase	ichor[c] + sucsal[c] -> 2shchc[c] + pyr[c]	Rv0555	Cofactor Metabolism
SSALy	succinate semialdehyde dehydrogenase NADP	h2o[c] + nadp[c] + sucsal[c] -> 2 h[c] + nadph[c] + succ[c]	Rv0234c Rv1731	Glutamate Metabolism
SUCBZL	o succinylbenzoate CoA ligase	atp[c] + coa[c] + sucbz[c] -> amp[c] + ppi[c] + sbzcoa[c]	Rv0542c	Ubiquinone Metabolism
SUCBZS	O succinylbenzoate CoA synthase	2shchc[c] -> h2o[c] + sucbz[c]	Rv0553	Ubiquinone Metabolism
THRS	threonine synthase	h2o[c] + phom[c] -> pi[c] + thr-L[c]	Rv1295	Glycine Serine and Threonine Metabolism
VALt2r	L valine reversible transport via proton symport	h[e] + val-L[e] <=> h[c] + val-L[c]		Transport

5 Table - S17 Metabolic Persister Reactions, Genes and Pathway on Rv1484 Deletion

Table 3: Metabolic persister reactions, gene and pathways on Rv1484 deletion

Reaction	Description	Formula	Genes	Pathways
ADK1	adenylate kinase	amp[c] + atp[c] <=> 2 adp[c]	Rv0733	Purine Metabolism
ADK2	adenylate kinase Inorganic triphosphate	amp[c] + pppi[c] <=> adp[c] + ppi[c]	Rv0733	Purine Metabolism
ADK4	adentylate kinase ITP	amp[c] + itp[c] <=> adp[c] + idp[c]	Rv0733	Nucleotide Sugar Metabolism
ALAD-L	L alanine dehydrogenase	ala-L[c] + h2o[c] + nad[c] -> h[c] + nadh[c] + nh4[c] + pyr[c]	Rv2780	Other Amino Acid Metabolism
ASPO5	L aspartate oxidase	asp-L[c] + fum[c] -> h[c] + iasp[c] + succ[c]	Rv1595	Alanine and Aspartate Metabolism
CAT	catalase	2 h2o2[c] -> 2 h2o[c] + o2[c]	Rv1908c	Redox Metabolism
CO2t	CO2 transporter via diffusion	co2[e] <=> co2[c]		Transport
CYTD	cytidine deaminase	cytd[c] + h2o[c] + h[c] -> nh4[c] + uri[c]	Rv3315c	Pyrimidine Metabolism
CYTDt2r	cytidine transport in via proton symport reversible	cytd[e] + h[e] <=> cytd[c] + h[c]		Transport
DADA	Deoxyadenosine deaminase	dad-2[c] + h2o[c] + h[c] -> din[c] + nh4[c]		Nucleotide Sugar Metabolism
DHORD3	dihydroorotic acid dehydrogenase menaquinone 6	dhor-S[c] + mqn6[c] <=> mql6[c] + orot[c]	Rv2139	Pyrimidine Metabolism
EX-co2(e)	CO2 exchange	co2[e] <=>		Exchange
EX-cytd(e)	Cytidine exchange	cytd[e] <=>		Exchange
EX-h2s(e)	Hydrogen sulfide exchange	h2s[e] <=>		Exchange
EX-lac-L(e)	L Lactate exchange	lac-L[e] <=>		Exchange
EX-na1(e)	Sodium exchange	na1[e] <=>		Exchange
FORMCOAL	formyl coa CoA ligase	atp[c] + coa[c] + for[c] <=> amp[c] + forcoa[c] + ppi[c]		Redox Metabolism
FRD2	fumarate reductase	fum[c] + mql8[c] -> mqn8[c] + succ[c]	Rv0247c Rv0248c Rv3316 Rv3317 Rv3318 Rv3319	Citric Acid Cycle

Continued on next page

Table 3 – continued from previous page

Reaction	Description	Formula	Genes	Pathways
FRD5	fumarate reductase	fum[c] + mql6[c] <=> mqn6[c] + succ[c]	Rv1552 Rv1553 Rv1554 Rv1555	Citric Acid Cycle
GCCa	glycine cleavage complex	gly[c] + h[c] + lpro[c] -> alpro[c] + co2[c]	Rv1832	Glycine Serine and Threonine Metabolism
GCCb	glycine cleavage complex	alpro[c] + thf[c] -> dhlpro[c] + mlthf[c] + nh4[c]	Rv2211c	Glycine Serine and Threonine Metabolism
GCCc	glycine cleavage complex	dhlpro[c] + nad[c] -> h[c] + lpro[c] + nadh[c]	Rv0794c Rv3303c	Methionine Metabolism
GLCP	glycogen phosphorylase	glycogen[c] + pi[c] -> g1p[c]	Rv1328	Sugar Metabolism
GLCS1	glycogen synthase ADPGlc	adpglc[c] -> adp[c] + glycogen[c] + h[c]	Rv1326c	Sugar Metabolism
GLGC	glucose 1 phosphate adenylyltransferase	atp[c] + g1p[c] + h[c] -> adpglc[c] + ppi[c]	Rv1213	Sugar Metabolism
GLXO1	glyoxylate oxidase	glx[c] + h2o[c] + nad[c] -> 2 h[c] + nadh[c] + oxa[c]		Glyoxylate Metabolism
GTHOr	glutathione oxidoreductase	gthox[c] + h[c] + nadph[c] <=> 2 gthrd[c] + nadp[c]	Rv2855	Glutamate Metabolism
HSDy	homoserine dehydrogenase NADPH	hom-L[c] + nadp[c] <=> aspsa[c] + h[c] + nadph[c]	Rv1294	Glycine Serine and Threonine Metabolism
Kt3r	potassium proton antiporter	h[e] + k[c] <=> h[c] + k[e]	Rv2691 Rv2692	Transport
L-LACD2	L Lactate dehydrogenase ubiquinone	lac-L[c] + q8[c] -> pyr[c] + q8h2[c]	Rv1872c	Pyruvate Metabolism
L-LACD3	L Lactate dehydrogenase menaquinone	lac-L[c] + mqn8[c] -> mql8[c] + pyr[c]	Rv1872c	Pyruvate Metabolism
L-LACt3	L lactate transport out via proton antiport	h[e] + lac-L[c] -> h[c] + lac-L[e]	Rv0191	Transport
ME1	malic enzyme NAD	mal-L[c] + nad[c] -> co2[c] + nadh[c] + pyr[c]	Rv2332	Pyruvate Metabolism
MYCTR	mycothiol reductase nadp dependent	2 msh[c] + nadp[c] <=> h[c] + mssg[c] + nadph[c]	Rv2855	Redox Metabolism
MYCTR2	mycothiol reductase glutathione exchange	gthox[c] + 2 msh[c] <=> 2 gthrd[c] + mssg[c]	Rv2855	Redox Metabolism

Continued on next page

Table 3 – continued from previous page

Reaction	Description	Formula	Genes	Pathways
NADH2r	NADH dehydrogenase	h[c] + nadh[c] + q[c] <=> nad[c] + qh2[c]	Rv0082 Rv3145 Rv3146 Rv3147 Rv3148 Rv3149 Rv3150 Rv3151 Rv3152 Rv3153 Rv3154 Rv3155 Rv3156 Rv3157 Rv3158	Redox Metabolism
NADS2	NAD synthase glutamine hydrolysing	atp[c] + dnad[c] + gln-L[c] + h2o[c] -> amp[c] + glu-L[c] + h[c] + nad[c] + ppi[c]	Rv2438c	Glutamate Metabolism
NAKtr	potassium sodium antiporter	k[c] + na1[e] <=> k[e] + na1[c]	Rv3236c	Transport
NAt3-1	sodium proton antiporter HNA is 11	h[e] + na1[c] <=> h[c] + na1[e]	Rv2287	Transport
NDPK9	nucleoside diphosphate kinase ATPIDP	atp[c] + idp[c] <=> adp[c] + itp[c]	Rv2445c	Pyrimidine Metabolism
NNAT	nicotinate nucleotide adenylyltransferase	atp[c] + h[c] + nicrnt[c] -> dnad[c] + ppi[c]	Rv2421c	Pantothenate and CoA Metabolism
NNDPR	nicotinate nucleotide diphosphorylase carboxylating	2 h[c] + prpp[c] + quln[c] -> co2[c] + nicrnt[c] + ppi[c]	Rv1596	Pantothenate and CoA Metabolism
NTD11	5 nucleotidase IMP	h2o[c] + imp[c] -> ins[c] + pi[c]		Nucleotide Sugar Metabolism
NTD2	5 nucleotidase UMP	h2o[c] + ump[c] -> pi[c] + uri[c]		Pyrimidine Metabolism
NTD7	5 nucleotidase AMP	amp[c] + h2o[c] -> adn[c] + pi[c]		Nucleotide Sugar Metabolism
OXACOAL	oxalate coa ligase	atp[c] + coa[c] + oxa[c] <=> amp[c] + oxalcoa[c] + ppi[c]		Glyoxylate Metabolism
OXCDC	Oxalyl CoA decarboxylase	h[c] + oxalcoa[c] -> co2[c] + forcoa[c]	Rv0118c	Glyoxylate Metabolism
PHTO	phthiocol oxygenase	2 o2[c] + phthclh2[c] -> 2 h[c] + 2 o2s[c] + phthcl[c]		Ubiquinone Metabolism
PPK2	polyphosphate kinase	atp[c] + ppi[c] -> adp[c] + pppi[c]	Rv2984	Purine Metabolism
PUNP2	purine nucleoside phosphorylase Deoxyadenosine	dad-2[c] + pi[c] <=> 2dr1p[c] + ade[c]	Rv3307	Purine Metabolism

Continued on next page

Table 3 – continued from previous page

Reaction	Description	Formula	Genes	Pathways
PUNP6	purine nucleoside phosphorylase Deoxyinosine	din[c] + pi[c] <=> 2dr1p[c] + hxan[c]	Rv3307	Purine Metabolism
QRr	Quinone reductase	h[c] + nadph[c] + q[c] <=> nadp[c] + qh2[c]	Rv3777	Redox Metabolism
QULNS	quinolinate synthase	dhap[c] + iasp[c] -> 2 h2o[c] + pi[c] + quln[c]	Rv1594	Pantothenate and CoA Metabolism
SPODM	superoxide dismutase	2 h[c] + 2 o2s[c] -> h2o2[c] + o2[c]	Rv0432 Rv1908c Rv3846	Cofactor Metabolism
XYLK	xylulokinase	atp[c] + xylu-D[c] -> adp[c] + h[c] + xu5p-D[c]	Rv0729	Sugar Metabolism
ALDR	Aldolase reductase	arab-D[c] + nadph[c] + h[c] -> arabl-D[c] + nadp[c]		Arabinogalactan Synthesis
AL4D	D-arabinitol 4-dehydrogenase	arabl-D[c] + nad[c] -> xylu-D[c] + nadh[c] + h[c]	Rv1928c	Arabinogalactan Synthesis

6 Fig. S3 - Spindle Map Rv2245

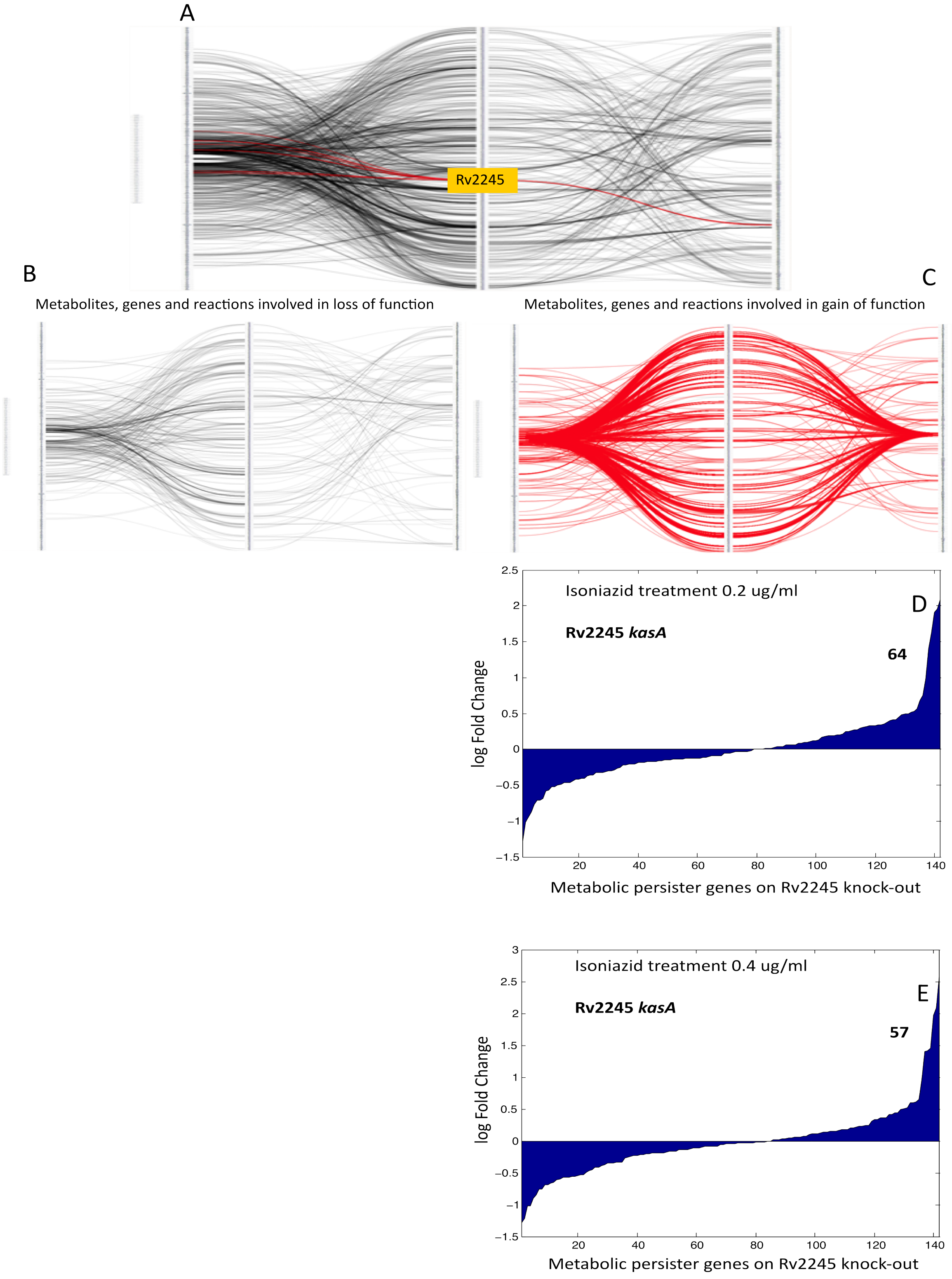

7 Table - S18 Metabolic Persister Reactions, Genes and Pathway on Rv2245 Deletion

Table 4: Metabolic persister reactions, gene and pathways on Rv2245 deletion

Reaction	Description	Formula	Genes	Pathways
ACKr	acetate kinase	ac[c] + atp[c] -> actp[c] + adp[c]	Rv0409	Pyruvate Metabolism
ADK1	adenylate kinase	amp[c] + atp[c] <=> 2 adp[c]	Rv0733	Purine Metabolism
ADK2	adenylate kinase Inorganic triphosphate	amp[c] + pppi[c] <=> adp[c] + ppi[c]	Rv0733	Purine Metabolism
ADK4	adentylate kinase ITP	amp[c] + itp[c] <=> adp[c] + idp[c]	Rv0733	Nucleotide Sugar Metabolism
ARGt5r	L arginine transport via diffusion	arg-L[e] <=> arg-L[c]	Rv2320c	Transport
ASPO5	L aspartate oxidase	asp-L[c] + fum[c] -> h[c] + iasp[c] + succ[c]	Rv1595	Alanine and Aspartate Metabolism
AST	Arginine succinyltransferase	arg-L[c] + succoa[c] -> coa[c] + h[c] + sucarg[c]		Arginine and Proline Metabolism
CAT	catalase	2 h2o2[c] -> 2 h2o[c] + o2[c]	Rv1908c	Redox Metabolism
CO2t	CO2 transporter via diffusion	co2[e] <=> co2[c]		Transport
CODH2r	carbon monoxide dehydrogenase acetyl CoA synthase 2	co[c] + 2 fdox[c] + h2o[c] <=> co2[c] + 2 fdred[c] + 2 h[c]	Rv0373c Rv0374c Rv0375c	Redox Metabolism
CODH3	carbon monoxide dehydrogenase acetyl CoA synthase 2	adfdOX[c] + 2 co[c] + 2 h2o[c] -> adfdRD[c] + 2 co2[c] + 2 h[c]	Rv0373c Rv0374c Rv0375c	Redox Metabolism
DCTPD2	dCTP deaminase	ctp[c] + h2o[c] + h[c] -> nh4[c] + utp[c]	Rv0321	Pyrimidine Metabolism
DHORD2	dihydoorotic acid dehydrogenase quinone8	dhor-S[c] + q8[c] -> orot[c] + q8h2[c]	Rv2139	Pyrimidine Metabolism
DHORD3	dihydroorotic acid dehydrogenase menaquinone 6	dhor-S[c] + mqn6[c] <=> mql6[c] + orot[c]	Rv2139	Pyrimidine Metabolism
ECHH-0	Ech hydrogenase no electron transport	2 fdox[c] + h2[c] <=> 2 fdred[c] + 2 h[c]		Miscellaneous

Continued on next page

Table 4 – continued from previous page

Reaction	Description	Formula	Genes	Pathways
ETHAt	ethanolamine transport via diffusion extracellular	etha[e] <=> etha[c]		Transport
EX-arg-L(e)	L Arginine exchange	arg-L[e] <=>		Exchange
EX-etha(e)	Ethanolamine exchange	etha[e] <=>		Exchange
EX-gly(e)	Glycine exchange	gly[e] <=>		Exchange
EX-k(e)	K exchange	k[e] <=>		Exchange
EX-mal-L(e)	L Malate exchange	mal-L[e] <=>		Exchange
EX-na1(e)	Sodium exchange	na1[e] <=>		Exchange
EX-ser-L(e)	L Serine exchange	ser-L[e] <=>		Exchange
FACOAL140	fatty acid CoA ligase tetradecanoate	atp[c] + coa[c] + ttdca[c] <=> amp[c] + ppi[c] + tdcoa[c]	Rv1529 Rv3826	Fatty Acid Metabolism
FACOAL161	fatty acid CoA ligase hexadecenoate	atp[c] + coa[c] + hdcea[c] <=> amp[c] + hdcoa[c] + ppi[c]	Rv1529 Rv3826	Fatty Acid Metabolism
FAO1	Fatty acid oxidation tetradecanoate	atp[c] + 7 coa[c] + 6 fad[c] + 6 h2o[c] + 6 nad[c] + ttdca[c] -> 7 accoa[c] + amp[c] + 6 fadh2[c] + 6 h[c] + 6 nadh[c] + ppi[c]	Rv0468 Rv0859 Rv0860 Rv1144 Rv1323	Fatty Acid Metabolism
FORMCOAL	formyl coa CoA ligase	atp[c] + coa[c] + for[c] <=> amp[c] + forcoa[c] + ppi[c]		Redox Metabolism
FRD	fumarate reductase	fadh2[c] + fum[c] -> fad[c] + succ[c]	Rv1552 Rv1553 Rv1554 Rv1555	Citric Acid Cycle
FRD2	fumarate reductase	fum[c] + mql8[c] -> mqn8[c] + succ[c]	Rv0247c Rv0248c Rv3316 Rv3317 Rv3318 Rv3319	Citric Acid Cycle
FRD5	fumarate reductase	fum[c] + mql6[c] <=> mqn6[c] + succ[c]	Rv1552 Rv1553 Rv1554 Rv1555	Citric Acid Cycle
G3PAT160	glycerol 3 phosphate acyltransferase C160	glyc3p[c] + palmACP[c] -> 1hdecg3p[c] + ACP[c]	Rv1551	Fatty Acid Metabolism
G6PDH2	glucose 6 phosphate dehydrogenase	g6p[c] + nadp[c] -> 6pgl[c] + 3 h[c] + nadph[c]	Rv1121 Rv1447c	Pentose Phosphate Pathway
GLYt2r	glycine reversible transport via proton symport	gly[e] + h[e] <=> gly[c] + h[c]	Rv1704c	Transport

Continued on next page

Table 4 – continued from previous page

Reaction	Description	Formula	Genes	Pathways
GPDDA2	Glycerophosphodiester phosphodiesterase Glycerophospho-ethanolamine	g3pe[c] + h2o[c] -> etha[c] + glyc3p[c] + h[c]	Rv0317c Rv2277c Rv3842c	Fatty Acid Metabolism
GTHOr	glutathione oxidoreductase	gthox[c] + h[c] + nadph[c] <=> 2 gthrd[c] + nadp[c]	Rv2855	Glutamate Metabolism
GTPDPK	GTP diphosphokinase	atp[c] + gtp[c] -> amp[c] + gdptp[c] + h[c]	Rv2583c	Purine Metabolism
HSDy	homoserine dehydrogenase NADPH	hom-L[c] + nadp[c] <=> aspsa[c] + h[c] + nadph[c]	Rv1294	Glycine Serine and Threonine Metabolism
Kt3r	potassium proton antiporter	h[e] + k[c] <=> h[c] + k[e]	Rv2691 Rv2692	Transport
LPLIPAL2E160	Lysophospholipase L2 2 acylglycerophospho-ethanolamine n C160	2agpe160[c] + h2o[c] -> g3pe[c] + h[c] + hdca[c]	Rv0183	Fatty Acid Metabolism
L-LACD2	L Lactate dehydrogenase ubiquinone	lac-L[c] + q8[c] -> pyr[c] + q8h2[c]	Rv1872c	Pyruvate Metabolism
MALt2r	L malate reversible transport via proton symport	h[e] + mal-L[e] <=> h[c] + mal-L[c]	Rv2443	Transport
ME1	malic enzyme NAD	mal-L[c] + nad[c] -> co2[c] + nadh[c] + pyr[c]	Rv2332	Pyruvate Metabolism
MYCTR	mycothiol reductase nadp dependent	2 msh[c] + nadp[c] <=> h[c] + mssg[c] + nadph[c]	Rv2855	Redox Metabolism
MYCTR2	mycothiol reductase glutathione exchange	gthox[c] + 2 msh[c] <=> 2 gthrd[c] + mssg[c]	Rv2855	Redox Metabolism
NADH2r	NADH dehydrogenase	h[c] + nadh[c] + q[c] <=> nad[c] + qh2[c]	Rv0082 Rv3145 Rv3146 Rv3147 Rv3148 Rv3149 Rv3150 Rv3151 Rv3152 Rv3153 Rv3154 Rv3155 Rv3156 Rv3157 Rv3158	Redox Metabolism
NADS2	NAD synthase glutamine hydrolysing	atp[c] + dnad[c] + gln-L[c] + h2o[c] -> amp[c] + glu-L[c] + h[c] + nad[c] + ppi[c]	Rv2438c	Glutamate Metabolism

Continued on next page

Table 4 – continued from previous page

Reaction	Description	Formula	Genes	Pathways
NAKtr	potassium sodium antiporter	k[c] + na1[e] <=> k[e] + na1[c]	Rv3236c	Transport
NAt3-1	sodium proton antiporter HNA is 11	h[e] + na1[c] <=> h[c] + na1[e]	Rv2287	Transport
NDPK9	nucleoside diphosphate kinase ATPIDP	atp[c] + idp[c] <=> adp[c] + itp[c]	Rv2445c	Pyrimidine Metabolism
NNAT	nicotinate nucleotide adenylyltransferase	atp[c] + h[c] + nicrnt[c] -> dnad[c] + ppi[c]	Rv2421c	Pantothenate and CoA Metabolism
NNDPR	nicotinate nucleotide diphosphorylase carboxylating	2 h[c] + prpp[c] + quln[c] -> co2[c] + nicrnt[c] + ppi[c]	Rv1596	Pantothenate and CoA Metabolism
ORNt	ornithine transport via diffusion extracellular to periplasm	orn[e] <=> orn[c]	Rv2320c	Transport
OXGDC	2 oxoglutarate decarboxylase	akg[c] + h[c] -> co2[c] + sucsal[c]	Rv1248c	Citric Acid Cycle
PGL	6 phosphogluconolactonase	6pgl[c] + h2o[c] -> 6pgc[c] + h[c]	Rv1445c	Pentose Phosphate Pathway
PHTO	phthiocol oxygenase	2 o2[c] + phthclh2[c] -> 2 h[c] + 2 o2s[c] + phthcl[c]		Ubiquinone Metabolism
PLIPA1E160	Phospholipase A1 phosphatidylethanolamine n C160	h2o[c] + pe160[c] -> 2agpe160[c] + h[c] + hdca[c]		Fatty Acid Metabolism
PPDK	Pyruvate phosphate dikinase	atp[c] + pi[c] + pyr[c] -> amp[c] + h[c] + pep[c] + ppi[c]	Rv1127c	Pyruvate Metabolism
PPK2	polyphosphate kinase	atp[c] + ppi[c] -> adp[c] + pppi[c]	Rv2984	Purine Metabolism
PTAr	phosphotransacetylase	accoa[c] + pi[c] <=> actp[c] + coa[c]	Rv0408 Rv3914	Pyruvate Metabolism
QRr	Quinone reductase	h[c] + nadph[c] + q[c] <=> nadp[c] + qh2[c]	Rv3777	Redox Metabolism
QULNS	quinolinate synthase	dhap[c] + iasp[c] -> 2 h2o[c] + pi[c] + quln[c]	Rv1594	Pantothenate and CoA Metabolism
SERt2r	L serine reversible transport via proton symport	h[e] + ser-L[e] <=> h[c] + ser-L[c]	Rv1704c	Transport
SGDS	Succinylglutamate desuccinylase	h2o[c] + sucglu[c] -> glu-L[c] + succ[c]		Arginine and Proline Metabolism

Continued on next page

Table 4 – continued from previous page

Reaction	Description	Formula	Genes	Pathways
SGSAD	Succinylglutamic semialdehyde dehydrogenase	h2o[c] + nad[c] + sucgsa[c] -> 2 h[c] + nadh[c] + sucglu[c]		Arginine and Proline Metabolism
SOTA	Succinylornithine transaminase	akg[c] + sucorn[c] -> glu-L[c] + sucgsa[c]		Arginine and Proline Metabolism
SPODM	superoxide dismutase	2 h[c] + 2 o2s[c] -> h2o2[c] + o2[c]	Rv0432 Rv1908c Rv3846	Cofactor Metabolism
SSALy	succinate semialdehyde dehydrogenase NADP	h2o[c] + nadp[c] + sucsal[c] -> 2 h[c] + nadph[c] + succ[c]	Rv0234c Rv1731	Glutamate Metabolism
XYLK	xylulokinase	atp[c] + xylu-D[c] -> adp[c] + h[c] + xu5p-D[c]	Rv0729	Sugar Metabolism
PPGPP	PPGPP-3-pyrophosphohydrolase	gdpdp[c] + h2o[c] -> gdp[c] + ppi[c]		Purine Metabolism

Continued on next page

Table 4 – continued from previous page

Reaction	Description	Formula	Genes	Pathways
FAD-8	acyl-CoA dehydrogenase fadE1-36 AND enoyl-CoA hydratase/isomerase echA1-21 AND 3-hydroxyacyl-CoA dehydrogenase fadB2-5 AND acetyl-CoA C-acetyltransferase fadA2-13	tdcoa[c] + fad[c] + nad[c] + coa[c] -> dodcoa[c] + fadh2[c] + nadh[c] + accoa[c]	Rv0131c Rv0154c Rv0215c Rv0231 Rv0244c Rv0271c Rv0400c Rv0752c Rv0873 Rv0972c Rv0975c Rv1346 Rv1467c Rv1679 Rv1933c Rv1934c Rv2724c Rv2789c Rv3061c Rv3139 Rv3140 Rv3274c Rv3504 Rv3505 Rv3543c Rv3544c Rv3560c Rv3562 Rv3563 Rv3564 Rv3573c Rv3761c Rv3797 Rv0672 Rv2500c Rv0222 Rv0456c Rv0632c Rv0673 Rv0675 Rv0971c Rv1070c Rv1071c Rv1141c Rv1142c Rv1472 Rv1935c Rv2486 Rv2679 Rv2831 Rv3039c Rv3516 Rv3550 Rv3774 Rv0905 Rv3374 Rv3373 Rv0468 Rv1715 Rv1912c Rv3141 Rv0243 Rv1074c Rv3546 Rv3556c Rv1323 Rv0859	Beta oxidation of fatty acids

Continued on next page

Table 4 – continued from previous page

Reaction	Description	Formula	Genes	Pathways
FAD-9	acyl-CoA dehydrogenase fadE1-36 AND enoyl-CoA hydratase/isomerase echA1-21 AND 3-hydroxyacyl-CoA dehydrogenase fadB2-5 AND acetyl-CoA C-acetyltransferase fadA2-14	dodcoa[c] + fad[c] + nad[c] + coa[c] -> decoa[c] + fadh2[c] + nadh[c] + accoa[c]	Rv0131c Rv0154c Rv0215c Rv0231 Rv0244c Rv0271c Rv0400c Rv0752c Rv0873 Rv0972c Rv0975c Rv1346 Rv1467c Rv1679 Rv1933c Rv1934c Rv2724c Rv2789c Rv3061c Rv3139 Rv3140 Rv3274c Rv3504 Rv3505 Rv3543c Rv3544c Rv3560c Rv3562 Rv3563 Rv3564 Rv3573c Rv3761c Rv3797 Rv0672 Rv2500c Rv0222 Rv0456c Rv0632c Rv0673 Rv0675 Rv0971c Rv1070c Rv1071c Rv1141c Rv1142c Rv1472 Rv1935c Rv2486 Rv2679 Rv2831 Rv3039c Rv3516 Rv3550 Rv3774 Rv0905 Rv3374 Rv3373 Rv0468 Rv1715 Rv1912c Rv3141 Rv0243 Rv1074c Rv3546 Rv3556c Rv1323 Rv0859	Beta oxidation of fatty acids

Continued on next page

Table 4 – continued from previous page

Reaction	Description	Formula	Genes	Pathways
FAD-11	acyl-CoA dehydrogenase fadE1-36 AND enoyl-CoA hydratase/isomerase echA1-21 AND 3-hydroxyacyl-CoA dehydrogenase fadB2-5 AND acetyl-CoA C-acetyltransferase fadA2-16	occoa[c] + fad[c] + nad[c] + coa[c] -> hexcoa[c] + fadh2[c] + nadh[c] + accoa[c]	Rv0131c Rv0154c Rv0215c Rv0231 Rv0244c Rv0271c Rv0400c Rv0752c Rv0873 Rv0972c Rv0975c Rv1346 Rv1467c Rv1679 Rv1933c Rv1934c Rv2724c Rv2789c Rv3061c Rv3139 Rv3140 Rv3274c Rv3504 Rv3505 Rv3543c Rv3544c Rv3560c Rv3562 Rv3563 Rv3564 Rv3573c Rv3761c Rv3797 Rv0672 Rv2500c Rv0222 Rv0456c Rv0632c Rv0673 Rv0675 Rv0971c Rv1070c Rv1071c Rv1141c Rv1142c Rv1472 Rv1935c Rv2486 Rv2679 Rv2831 Rv3039c Rv3516 Rv3550 Rv3774 Rv0905 Rv3374 Rv3373 Rv0468 Rv1715 Rv1912c Rv3141 Rv0243 Rv1074c Rv3546 Rv3556c Rv1323 Rv0859	Beta oxidation of fatty acids

Continued on next page

Table 4 – continued from previous page

Reaction	Description	Formula	Genes	Pathways
FAD-15	acyl-CoA dehydrogenase fadE1-36 AND enoyl-CoA hydratase/isomerase echA1-21 AND 3-hydroxyacyl-CoA dehydrogenase fadB2-5 AND acetyl-CoA C-acetyltransferase fadA2-20	hdcoa[c] + fad[c] + nad[c] + coa[c] -> c5tetdcoa[c] + fadh2[c] + nadh[c] + accoa[c]	Rv0131c Rv0154c Rv0215c Rv0231 Rv0244c Rv0271c Rv0400c Rv0752c Rv0873 Rv0972c Rv0975c Rv1346 Rv1467c Rv1679 Rv1933c Rv1934c Rv2724c Rv2789c Rv3061c Rv3139 Rv3140 Rv3274c Rv3504 Rv3505 Rv3543c Rv3544c Rv3560c Rv3562 Rv3563 Rv3564 Rv3573c Rv3761c Rv3797 Rv0672 Rv2500c Rv0222 Rv0456c Rv0632c Rv0673 Rv0675 Rv0971c Rv1070c Rv1071c Rv1141c Rv1142c Rv1472 Rv1935c Rv2486 Rv2679 Rv2831 Rv3039c Rv3516 Rv3550 Rv3774 Rv0905 Rv3374 Rv3373 Rv0468 Rv1715 Rv1912c Rv3141 Rv0243 Rv1074c Rv3546 Rv3556c Rv1323 Rv0859	Beta oxidation of unsaturated fatty acids

Continued on next page

Table 4 – continued from previous page

Reaction	Description	Formula	Genes	Pathways
FAD-16	acyl-CoA dehydrogenase fadE1-36 AND enoyl-CoA hydratase/isomerase echA1-21 AND 3-hydroxyacyl-CoA dehydrogenase fadB2-5 AND acetyl-CoA C-acetyltransferase fadA2-21	c5tetdcoa[c] + fad[c] + nad[c] + coa[c] -> c3dodcoa[c] + fadh2[c] + nadh[c] + accoa[c]	Rv0131c Rv0154c Rv0215c Rv0231 Rv0244c Rv0271c Rv0400c Rv0752c Rv0873 Rv0972c Rv0975c Rv1346 Rv1467c Rv1679 Rv1933c Rv1934c Rv2724c Rv2789c Rv3061c Rv3139 Rv3140 Rv3274c Rv3504 Rv3505 Rv3543c Rv3544c Rv3560c Rv3562 Rv3563 Rv3564 Rv3573c Rv3761c Rv3797 Rv0672 Rv2500c Rv0222 Rv0456c Rv0632c Rv0673 Rv0675 Rv0971c Rv1070c Rv1071c Rv1141c Rv1142c Rv1472 Rv1935c Rv2486 Rv2679 Rv2831 Rv3039c Rv3516 Rv3550 Rv3774 Rv0905 Rv3374 Rv3373 Rv0468 Rv1715 Rv1912c Rv3141 Rv0243 Rv1074c Rv3546 Rv3556c Rv1323 Rv0859	Beta oxidation of unsaturated fatty acids

Continued on next page

Table 4 – continued from previous page

Reaction	Description	Formula	Genes	Pathways
FAD-17	acyl-CoA dehydrogenase fadE1-36 AND enoyl-CoA hydratase/isomerase echA1-21 AND 3-hydroxyacyl-CoA dehydrogenase fadB2-5 AND acetyl-CoA C-acetyltransferase fadA2-22	c3dodcoa[c] -> t2dodcoa[c]	Rv0131c Rv0154c Rv0215c Rv0231 Rv0244c Rv0271c Rv0400c Rv0752c Rv0873 Rv0972c Rv0975c Rv1346 Rv1467c Rv1679 Rv1933c Rv1934c Rv2724c Rv2789c Rv3061c Rv3139 Rv3140 Rv3274c Rv3504 Rv3505 Rv3543c Rv3544c Rv3560c Rv3562 Rv3563 Rv3564 Rv3573c Rv3761c Rv3797 Rv0672 Rv2500c Rv0222 Rv0456c Rv0632c Rv0673 Rv0675 Rv0971c Rv1070c Rv1071c Rv1141c Rv1142c Rv1472 Rv1935c Rv2486 Rv2679 Rv2831 Rv3039c Rv3516 Rv3550 Rv3774 Rv0905 Rv3374 Rv3373 Rv0468 Rv1715 Rv1912c Rv3141 Rv0243 Rv1074c Rv3546 Rv3556c Rv1323 Rv0859	Beta oxidation of unsaturated fatty acids

Continued on next page

Table 4 – continued from previous page

Reaction	Description	Formula	Genes	Pathways
FAD-19	acyl-CoA dehydrogenase fadE1-36 AND enoyl-CoA hydratase/isomerase echA1-21 AND 3-hydroxyacyl-CoA dehydrogenase fadB2-5 AND acetyl-CoA C-acetyltransferase fadA2-24	d2hdodcoa[c] + nad[c] -> dodcoa[c] + nadh[c]	Rv0131c Rv0154c Rv0215c Rv0231 Rv0244c Rv0271c Rv0400c Rv0752c Rv0873 Rv0972c Rv0975c Rv1346 Rv1467c Rv1679 Rv1933c Rv1934c Rv2724c Rv2789c Rv3061c Rv3139 Rv3140 Rv3274c Rv3504 Rv3505 Rv3543c Rv3544c Rv3560c Rv3562 Rv3563 Rv3564 Rv3573c Rv3761c Rv3797 Rv0672 Rv2500c Rv0222 Rv0456c Rv0632c Rv0673 Rv0675 Rv0971c Rv1070c Rv1071c Rv1141c Rv1142c Rv1472 Rv1935c Rv2486 Rv2679 Rv2831 Rv3039c Rv3516 Rv3550 Rv3774 Rv0905 Rv3374 Rv3373 Rv0468 Rv1715 Rv1912c Rv3141 Rv0243 Rv1074c Rv3546 Rv3556c Rv1323 Rv0859	Beta oxidation of unsaturated fatty acids
ALDR	Aldolase reductase	arab-D[c] + nadph[c] + h[c] -> arabl-D[c] + nadp[c]		Arabinogalactan Synthesis
AL4D	D-arabinitol 4-dehydrogenase	arabl-D[c] + nad[c] -> xylu-D[c] + nadh[c] + h[c]	Rv1928c	Arabinogalactan Synthesis

8 Fig. S4 - Spindle Map Rv3794

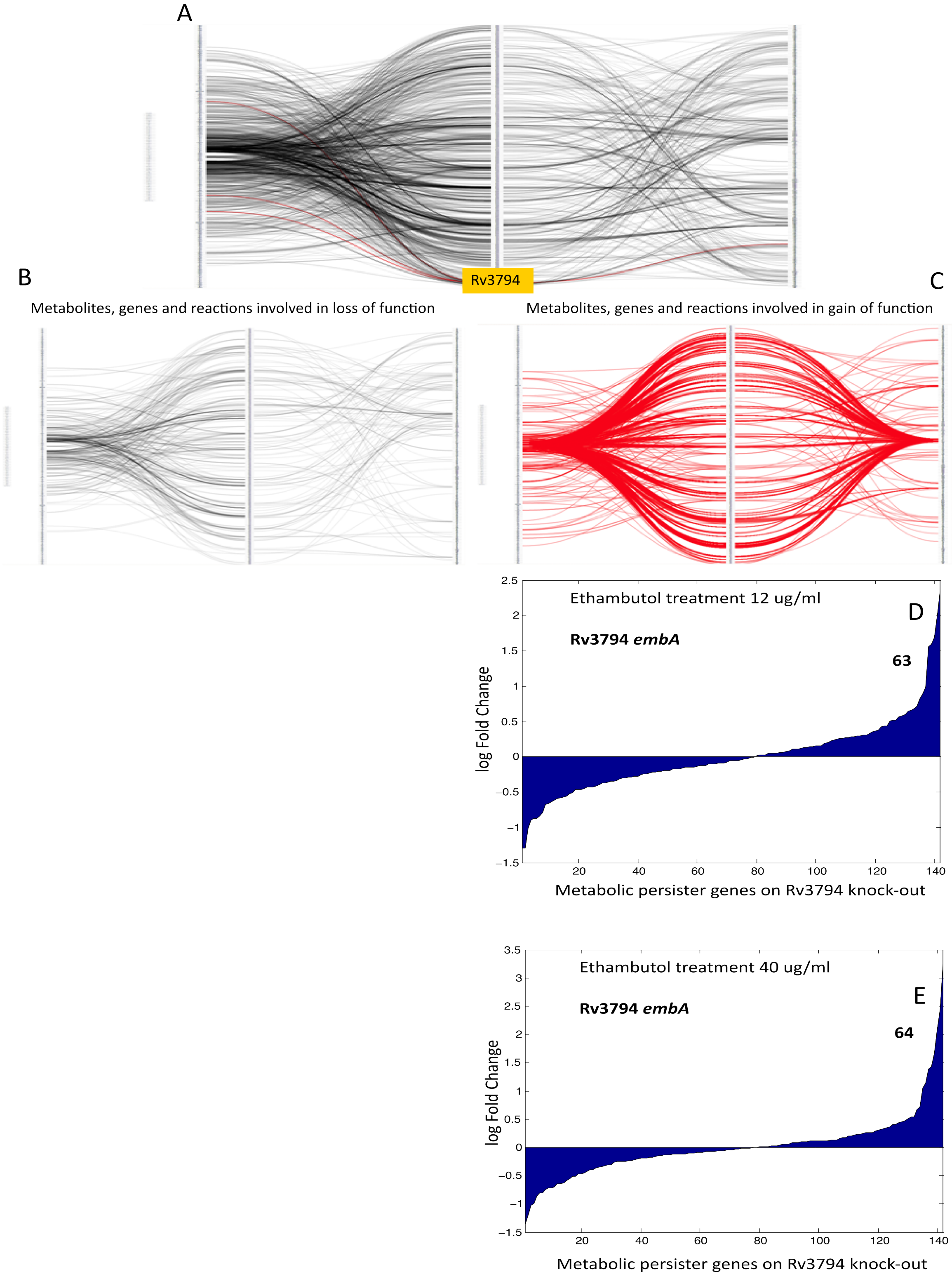

9 Table - S19 Metabolic Persister Reactions, Genes and Pathway on Rv3794 Deletion

Table 5: Metabolic persister reactions, gene and pathways on Rv3794 deletion

Reaction	Description	Formula	Genes	Pathways
ACKr	acetate kinase	ac[c] + atp[c] -> actp[c] + adp[c]	Rv0409	Pyruvate Metabolism
ADK1	adenylate kinase	amp[c] + atp[c] <=> 2 adp[c]	Rv0733	Purine Metabolism
ADK2	adenylate kinase Inorganic triphosphate	amp[c] + pppi[c] <=> adp[c] + ppi[c]	Rv0733	Purine Metabolism
ADK4	adentylate kinase ITP	amp[c] + itp[c] <=> adp[c] + idp[c]	Rv0733	Nucleotide Sugar Metabolism
ARGt5r	L arginine transport via diffusion	arg-L[e] <=> arg-L[c]	Rv2320c	Transport
ASPO5	L aspartate oxidase	asp-L[c] + fum[c] -> h[c] + iasp[c] + succ[c]	Rv1595	Alanine and Aspartate Metabolism
AST	Arginine succinyltransferase	arg-L[c] + succoa[c] -> coa[c] + h[c] + sucarg[c]		Arginine and Proline Metabolism
CAT	catalase	2 h2o2[c] -> 2 h2o[c] + o2[c]	Rv1908c	Redox Metabolism
CO2t	CO2 transporter via diffusion	co2[e] <=> co2[c]		Transport
CODH2r	carbon monoxide dehydrogenase acetyl CoA synthase 2	co[c] + 2 fdox[c] + h2o[c] <=> co2[c] + 2 fdred[c] + 2 h[c]	Rv0373c Rv0374c Rv0375c	Redox Metabolism
CODH3	carbon monoxide dehydrogenase acetyl CoA synthase 2	adfdOX[c] + 2 co[c] + 2 h2o[c] -> adfdRD[c] + 2 co2[c] + 2 h[c]	Rv0373c Rv0374c Rv0375c	Redox Metabolism
DCTPD2	dCTP deaminase	ctp[c] + h2o[c] + h[c] -> nh4[c] + utp[c]	Rv0321	Pyrimidine Metabolism
DHORD2	dihydoorotic acid dehydrogenase quinone8	dhor-S[c] + q8[c] -> orot[c] + q8h2[c]	Rv2139	Pyrimidine Metabolism
DHORD3	dihydroorotic acid dehydrogenase menaquinone 6	dhor-S[c] + mqn6[c] <=> mql6[c] + orot[c]	Rv2139	Pyrimidine Metabolism
ECHH-0	Ech hydrogenase no electron transport	2 fdox[c] + h2[c] <=> 2 fdred[c] + 2 h[c]		Miscellaneous

Continued on next page

Table 5 – continued from previous page

Reaction	Description	Formula	Genes	Pathways
ETHAt	ethanolamine transport via diffusion extracellular	etha[e] <=> etha[c]		Transport
EX-arg-L(e)	L Arginine exchange	arg-L[e] <=>		Exchange
EX-etha(e)	Ethanolamine exchange	etha[e] <=>		Exchange
EX-gly(e)	Glycine exchange	gly[e] <=>		Exchange
EX-k(e)	K exchange	k[e] <=>		Exchange
EX-mal-L(e)	L Malate exchange	mal-L[e] <=>		Exchange
EX-na1(e)	Sodium exchange	na1[e] <=>		Exchange
EX-ser-L(e)	L Serine exchange	ser-L[e] <=>		Exchange
FACOAL140	fatty acid CoA ligase tetradecanoate	atp[c] + coa[c] + ttdca[c] <=> amp[c] + ppi[c] + tdcoa[c]	Rv1529 Rv3826	Fatty Acid Metabolism
FACOAL161	fatty acid CoA ligase hexadecenoate	atp[c] + coa[c] + hdcea[c] <=> amp[c] + hdcoa[c] + ppi[c]	Rv1529 Rv3826	Fatty Acid Metabolism
FAO1	Fatty acid oxidation tetradecanoate	atp[c] + 7 coa[c] + 6 fad[c] + 6 h2o[c] + 6 nad[c] + ttdca[c] -> 7 accoa[c] + amp[c] + 6 fadh2[c] + 6 h[c] + 6 nadh[c] + ppi[c]	Rv0468 Rv0859 Rv0860 Rv1144 Rv1323	Fatty Acid Metabolism
FORMCOAL	formyl coa CoA ligase	atp[c] + coa[c] + for[c] <=> amp[c] + forcoa[c] + ppi[c]		Redox Metabolism
FRD	fumarate reductase	fadh2[c] + fum[c] -> fad[c] + succ[c]	Rv1552 Rv1553 Rv1554 Rv1555	Citric Acid Cycle
FRD2	fumarate reductase	fum[c] + mql8[c] -> mqn8[c] + succ[c]	Rv0247c Rv0248c Rv3316 Rv3317 Rv3318 Rv3319	Citric Acid Cycle
FRD5	fumarate reductase	fum[c] + mql6[c] <=> mqn6[c] + succ[c]	Rv1552 Rv1553 Rv1554 Rv1555	Citric Acid Cycle
G3PAT160	glycerol 3 phosphate acyltransferase C160	glyc3p[c] + palmACP[c] -> 1hdecg3p[c] + ACP[c]	Rv1551	Fatty Acid Metabolism
G6PDH2	glucose 6 phosphate dehydrogenase	g6p[c] + nadp[c] -> 6pgl[c] + 3 h[c] + nadph[c]	Rv1121 Rv1447c	Pentose Phosphate Pathway
GLYt2r	glycine reversible transport via proton symport	gly[e] + h[e] <=> gly[c] + h[c]	Rv1704c	Transport

Continued on next page

Table 5 – continued from previous page

Reaction	Description	Formula	Genes	Pathways
GPDDA2	Glycerophosphodiester phosphodiesterase Glycerophospho-ethanolamine	g3pe[c] + h2o[c] -> etha[c] + glyc3p[c] + h[c]	Rv0317c Rv2277c Rv3842c	Fatty Acid Metabolism
GTHOr	glutathione oxidoreductase	gthox[c] + h[c] + nadph[c] <=> 2 gthrd[c] + nadp[c]	Rv2855	Glutamate Metabolism
GTPDPK	GTP diphosphokinase	atp[c] + gtp[c] -> amp[c] + gdptp[c] + h[c]	Rv2583c	Purine Metabolism
HSDy	homoserine dehydrogenase NADPH	hom-L[c] + nadp[c] <=> aspsa[c] + h[c] + nadph[c]	Rv1294	Glycine Serine and Threonine Metabolism
Kt3r	potassium proton antiporter	h[e] + k[c] <=> h[c] + k[e]	Rv2691 Rv2692	Transport
LPLIPAL2E160	Lysophospholipase L2 2 acylglycerophospho-ethanolamine n C160	2agpe160[c] + h2o[c] -> g3pe[c] + h[c] + hdca[c]	Rv0183	Fatty Acid Metabolism
L-LACD2	L Lactate dehydrogenase ubiquinone	lac-L[c] + q8[c] -> pyr[c] + q8h2[c]	Rv1872c	Pyruvate Metabolism
MALt2r	L malate reversible transport via proton symport	h[e] + mal-L[e] <=> h[c] + mal-L[c]	Rv2443	Transport
ME1	malic enzyme NAD	mal-L[c] + nad[c] -> co2[c] + nadh[c] + pyr[c]	Rv2332	Pyruvate Metabolism
MYCTR	mycothiol reductase nadp dependent	2 msh[c] + nadp[c] <=> h[c] + mssg[c] + nadph[c]	Rv2855	Redox Metabolism
MYCTR2	mycothiol reductase glutathione exchange	gthox[c] + 2 msh[c] <=> 2 gthrd[c] + mssg[c]	Rv2855	Redox Metabolism
NADH2r	NADH dehydrogenase	h[c] + nadh[c] + q[c] <=> nad[c] + qh2[c]	Rv0082 Rv3145 Rv3146 Rv3147 Rv3148 Rv3149 Rv3150 Rv3151 Rv3152 Rv3153 Rv3154 Rv3155 Rv3156 Rv3157 Rv3158	Redox Metabolism
NADS2	NAD synthase glutamine hydrolysing	atp[c] + dnad[c] + gln-L[c] + h2o[c] -> amp[c] + glu-L[c] + h[c] + nad[c] + ppi[c]	Rv2438c	Glutamate Metabolism

Continued on next page

Table 5 – continued from previous page

Reaction	Description	Formula	Genes	Pathways
NAKtr	potassium sodium antiporter	k[c] + na1[e] <=> k[e] + na1[c]	Rv3236c	Transport
NAt3-1	sodium proton antiporter HNA is 11	h[e] + na1[c] <=> h[c] + na1[e]	Rv2287	Transport
NDPK9	nucleoside diphosphate kinase ATPIDP	atp[c] + idp[c] <=> adp[c] + itp[c]	Rv2445c	Pyrimidine Metabolism
NNAT	nicotinate nucleotide adenylyltransferase	atp[c] + h[c] + nicrnt[c] -> dnad[c] + ppi[c]	Rv2421c	Pantothenate and CoA Metabolism
NNDPR	nicotinate nucleotide diphosphorylase carboxylating	2 h[c] + prpp[c] + quln[c] -> co2[c] + nicrnt[c] + ppi[c]	Rv1596	Pantothenate and CoA Metabolism
ORNt	ornithine transport via diffusion extracellular to periplasm	orn[e] <=> orn[c]	Rv2320c	Transport
OXGDC	2 oxoglutarate decarboxylase	akg[c] + h[c] -> co2[c] + sucsal[c]	Rv1248c	Citric Acid Cycle
PGL	6 phosphogluconolactonase	6pgl[c] + h2o[c] -> 6pgc[c] + h[c]	Rv1445c	Pentose Phosphate Pathway
PHTO	phthiocol oxygenase	2 o2[c] + phthclh2[c] -> 2 h[c] + 2 o2s[c] + phthcl[c]		Ubiquinone Metabolism
PLIPA1E160	Phospholipase A1 phosphatidylethanolamine n C160	h2o[c] + pe160[c] -> 2agpe160[c] + h[c] + hdca[c]		Fatty Acid Metabolism
PPDK	Pyruvate phosphate dikinase	atp[c] + pi[c] + pyr[c] -> amp[c] + h[c] + pep[c] + ppi[c]	Rv1127c	Pyruvate Metabolism
PPK2	polyphosphate kinase	atp[c] + ppi[c] -> adp[c] + pppi[c]	Rv2984	Purine Metabolism
PTAr	phosphotransacetylase	accoa[c] + pi[c] <=> actp[c] + coa[c]	Rv0408 Rv3914	Pyruvate Metabolism
QRr	Quinone reductase	h[c] + nadph[c] + q[c] <=> nadp[c] + qh2[c]	Rv3777	Redox Metabolism
QULNS	quinolinate synthase	dhap[c] + iasp[c] -> 2 h2o[c] + pi[c] + quln[c]	Rv1594	Pantothenate and CoA Metabolism
SERt2r	L serine reversible transport via proton symport	h[e] + ser-L[e] <=> h[c] + ser-L[c]	Rv1704c	Transport
SGDS	Succinylglutamate desuccinylase	h2o[c] + sucglu[c] -> glu-L[c] + succ[c]		Arginine and Proline Metabolism

Continued on next page

Table 5 – continued from previous page

Reaction	Description	Formula	Genes	Pathways
SGSAD	Succinylglutamic semialdehyde dehydrogenase	h2o[c] + nad[c] + sucgsa[c] -> 2 h[c] + nadh[c] + sucglu[c]		Arginine and Proline Metabolism
SOTA	Succinylornithine transaminase	akg[c] + sucorn[c] -> glu-L[c] + sucgsa[c]		Arginine and Proline Metabolism
SPODM	superoxide dismutase	2 h[c] + 2 o2s[c] -> h2o2[c] + o2[c]	Rv0432 Rv1908c Rv3846	Cofactor Metabolism
SSALy	succinate semialdehyde dehydrogenase NADP	h2o[c] + nadp[c] + sucsal[c] -> 2 h[c] + nadph[c] + succ[c]	Rv0234c Rv1731	Glutamate Metabolism
XYLK	xylulokinase	atp[c] + xylu-D[c] -> adp[c] + h[c] + xu5p-D[c]	Rv0729	Sugar Metabolism
PPGPP	PPGPP-3-pyrophosphohydrolase	gdpdp[c] + h2o[c] -> gdp[c] + ppi[c]		Purine Metabolism

Continued on next page

Table 5 – continued from previous page

Reaction	Description	Formula	Genes	Pathways
FAD-8	acyl-CoA dehydrogenase fadE1-36 AND enoyl-CoA hydratase/isomerase echA1-21 AND 3-hydroxyacyl-CoA dehydrogenase fadB2-5 AND acetyl-CoA C-acetyltransferase fadA2-13	tdcoa[c] + fad[c] + nad[c] + coa[c] -> dodcoa[c] + fadh2[c] + nadh[c] + accoa[c]	Rv0131c Rv0154c Rv0215c Rv0231 Rv0244c Rv0271c Rv0400c Rv0752c Rv0873 Rv0972c Rv0975c Rv1346 Rv1467c Rv1679 Rv1933c Rv1934c Rv2724c Rv2789c Rv3061c Rv3139 Rv3140 Rv3274c Rv3504 Rv3505 Rv3543c Rv3544c Rv3560c Rv3562 Rv3563 Rv3564 Rv3573c Rv3761c Rv3797 Rv0672 Rv2500c Rv0222 Rv0456c Rv0632c Rv0673 Rv0675 Rv0971c Rv1070c Rv1071c Rv1141c Rv1142c Rv1472 Rv1935c Rv2486 Rv2679 Rv2831 Rv3039c Rv3516 Rv3550 Rv3774 Rv0905 Rv3374 Rv3373 Rv0468 Rv1715 Rv1912c Rv3141 Rv0243 Rv1074c Rv3546 Rv3556c Rv1323 Rv0859	Beta oxidation of fatty acids

Continued on next page

Table 5 – continued from previous page

Reaction	Description	Formula	Genes	Pathways
FAD-9	acyl-CoA dehydrogenase fadE1-36 AND enoyl-CoA hydratase/isomerase echA1-21 AND 3-hydroxyacyl-CoA dehydrogenase fadB2-5 AND acetyl-CoA C-acetyltransferase fadA2-14	dodcoa[c] + fad[c] + nad[c] + coa[c] -> decoa[c] + fadh2[c] + nadh[c] + accoa[c]	Rv0131c Rv0154c Rv0215c Rv0231 Rv0244c Rv0271c Rv0400c Rv0752c Rv0873 Rv0972c Rv0975c Rv1346 Rv1467c Rv1679 Rv1933c Rv1934c Rv2724c Rv2789c Rv3061c Rv3139 Rv3140 Rv3274c Rv3504 Rv3505 Rv3543c Rv3544c Rv3560c Rv3562 Rv3563 Rv3564 Rv3573c Rv3761c Rv3797 Rv0672 Rv2500c Rv0222 Rv0456c Rv0632c Rv0673 Rv0675 Rv0971c Rv1070c Rv1071c Rv1141c Rv1142c Rv1472 Rv1935c Rv2486 Rv2679 Rv2831 Rv3039c Rv3516 Rv3550 Rv3774 Rv0905 Rv3374 Rv3373 Rv0468 Rv1715 Rv1912c Rv3141 Rv0243 Rv1074c Rv3546 Rv3556c Rv1323 Rv0859	Beta oxidation of fatty acids

Continued on next page

Table 5 – continued from previous page

Reaction	Description	Formula	Genes	Pathways
FAD-11	acyl-CoA dehydrogenase fadE1-36 AND enoyl-CoA hydratase/isomerase echA1-21 AND 3-hydroxyacyl-CoA dehydrogenase fadB2-5 AND acetyl-CoA C-acetyltransferase fadA2-16	occoa[c] + fad[c] + nad[c] + coa[c] -> hexcoa[c] + fadh2[c] + nadh[c] + accoa[c]	Rv0131c Rv0154c Rv0215c Rv0231 Rv0244c Rv0271c Rv0400c Rv0752c Rv0873 Rv0972c Rv0975c Rv1346 Rv1467c Rv1679 Rv1933c Rv1934c Rv2724c Rv2789c Rv3061c Rv3139 Rv3140 Rv3274c Rv3504 Rv3505 Rv3543c Rv3544c Rv3560c Rv3562 Rv3563 Rv3564 Rv3573c Rv3761c Rv3797 Rv0672 Rv2500c Rv0222 Rv0456c Rv0632c Rv0673 Rv0675 Rv0971c Rv1070c Rv1071c Rv1141c Rv1142c Rv1472 Rv1935c Rv2486 Rv2679 Rv2831 Rv3039c Rv3516 Rv3550 Rv3774 Rv0905 Rv3374 Rv3373 Rv0468 Rv1715 Rv1912c Rv3141 Rv0243 Rv1074c Rv3546 Rv3556c Rv1323 Rv0859	Beta oxidation of fatty acids

Continued on next page

Table 5 – continued from previous page

Reaction	Description	Formula	Genes	Pathways
FAD-15	acyl-CoA dehydrogenase fadE1-36 AND enoyl-CoA hydratase/isomerase echA1-21 AND 3-hydroxyacyl-CoA dehydrogenase fadB2-5 AND acetyl-CoA C-acetyltransferase fadA2-20	hdcoa[c] + fad[c] + nad[c] + coa[c] -> c5tetdcoa[c] + fadh2[c] + nadh[c] + accoa[c]	Rv0131c Rv0154c Rv0215c Rv0231 Rv0244c Rv0271c Rv0400c Rv0752c Rv0873 Rv0972c Rv0975c Rv1346 Rv1467c Rv1679 Rv1933c Rv1934c Rv2724c Rv2789c Rv3061c Rv3139 Rv3140 Rv3274c Rv3504 Rv3505 Rv3543c Rv3544c Rv3560c Rv3562 Rv3563 Rv3564 Rv3573c Rv3761c Rv3797 Rv0672 Rv2500c Rv0222 Rv0456c Rv0632c Rv0673 Rv0675 Rv0971c Rv1070c Rv1071c Rv1141c Rv1142c Rv1472 Rv1935c Rv2486 Rv2679 Rv2831 Rv3039c Rv3516 Rv3550 Rv3774 Rv0905 Rv3374 Rv3373 Rv0468 Rv1715 Rv1912c Rv3141 Rv0243 Rv1074c Rv3546 Rv3556c Rv1323 Rv0859	Beta oxidation of unsaturated fatty acids
Continued on next page				

Table 5 – continued from previous page

Reaction	Description	Formula	Genes	Pathways
FAD-16	acyl-CoA dehydrogenase fadE1-36 AND enoyl-CoA hydratase/isomerase echA1-21 AND 3-hydroxyacyl-CoA dehydrogenase fadB2-5 AND acetyl-CoA C-acetyltransferase fadA2-21	c5tetdcoa[c] + fad[c] + nad[c] + coa[c] -> c3dodcoa[c] + fadh2[c] + nadh[c] + accoa[c]	Rv0131c Rv0154c Rv0215c Rv0231 Rv0244c Rv0271c Rv0400c Rv0752c Rv0873 Rv0972c Rv0975c Rv1346 Rv1467c Rv1679 Rv1933c Rv1934c Rv2724c Rv2789c Rv3061c Rv3139 Rv3140 Rv3274c Rv3504 Rv3505 Rv3543c Rv3544c Rv3560c Rv3562 Rv3563 Rv3564 Rv3573c Rv3761c Rv3797 Rv0672 Rv2500c Rv0222 Rv0456c Rv0632c Rv0673 Rv0675 Rv0971c Rv1070c Rv1071c Rv1141c Rv1142c Rv1472 Rv1935c Rv2486 Rv2679 Rv2831 Rv3039c Rv3516 Rv3550 Rv3774 Rv0905 Rv3374 Rv3373 Rv0468 Rv1715 Rv1912c Rv3141 Rv0243 Rv1074c Rv3546 Rv3556c Rv1323 Rv0859	Beta oxidation of unsaturated fatty acids

Continued on next page

Table 5 – continued from previous page

Reaction	Description	Formula	Genes	Pathways
FAD-17	acyl-CoA dehydrogenase fadE1-36 AND enoyl-CoA hydratase/isomerase echA1-21 AND 3-hydroxyacyl-CoA dehydrogenase fadB2-5 AND acetyl-CoA C-acetyltransferase fadA2-22	c3dodcoa[c] -> t2dodcoa[c]	Rv0131c Rv0154c Rv0215c Rv0231 Rv0244c Rv0271c Rv0400c Rv0752c Rv0873 Rv0972c Rv0975c Rv1346 Rv1467c Rv1679 Rv1933c Rv1934c Rv2724c Rv2789c Rv3061c Rv3139 Rv3140 Rv3274c Rv3504 Rv3505 Rv3543c Rv3544c Rv3560c Rv3562 Rv3563 Rv3564 Rv3573c Rv3761c Rv3797 Rv0672 Rv2500c Rv0222 Rv0456c Rv0632c Rv0673 Rv0675 Rv0971c Rv1070c Rv1071c Rv1141c Rv1142c Rv1472 Rv1935c Rv2486 Rv2679 Rv2831 Rv3039c Rv3516 Rv3550 Rv3774 Rv0905 Rv3374 Rv3373 Rv0468 Rv1715 Rv1912c Rv3141 Rv0243 Rv1074c Rv3546 Rv3556c Rv1323 Rv0859	Beta oxidation of unsaturated fatty acids

Continued on next page

Table 5 – continued from previous page

Reaction	Description	Formula	Genes	Pathways
FAD-19	acyl-CoA dehydrogenase fadE1-36 AND enoyl-CoA hydratase/isomerase echA1-21 AND 3-hydroxyacyl-CoA dehydrogenase fadB2-5 AND acetyl-CoA C-acetyltransferase fadA2-24	d2hdodcoa[c] + nad[c] -> dodcoa[c] + nadh[c]	Rv0131c Rv0154c Rv0215c Rv0231 Rv0244c Rv0271c Rv0400c Rv0752c Rv0873 Rv0972c Rv0975c Rv1346 Rv1467c Rv1679 Rv1933c Rv1934c Rv2724c Rv2789c Rv3061c Rv3139 Rv3140 Rv3274c Rv3504 Rv3505 Rv3543c Rv3544c Rv3560c Rv3562 Rv3563 Rv3564 Rv3573c Rv3761c Rv3797 Rv0672 Rv2500c Rv0222 Rv0456c Rv0632c Rv0673 Rv0675 Rv0971c Rv1070c Rv1071c Rv1141c Rv1142c Rv1472 Rv1935c Rv2486 Rv2679 Rv2831 Rv3039c Rv3516 Rv3550 Rv3774 Rv0905 Rv3374 Rv3373 Rv0468 Rv1715 Rv1912c Rv3141 Rv0243 Rv1074c Rv3546 Rv3556c Rv1323 Rv0859	Beta oxidation of unsaturated fatty acids
ALDR	Aldolase reductase	arab-D[c] + nadph[c] + h[c] -> arabl-D[c] + nadp[c]		Arabinogalactan Synthesis
AL4D	D-arabinitol 4-dehydrogenase	arabl-D[c] + nad[c] -> xylu-D[c] + nadh[c] + h[c]	Rv1928c	Arabinogalactan Synthesis

10 Fig. S5 - Spindle Map Rv3264c

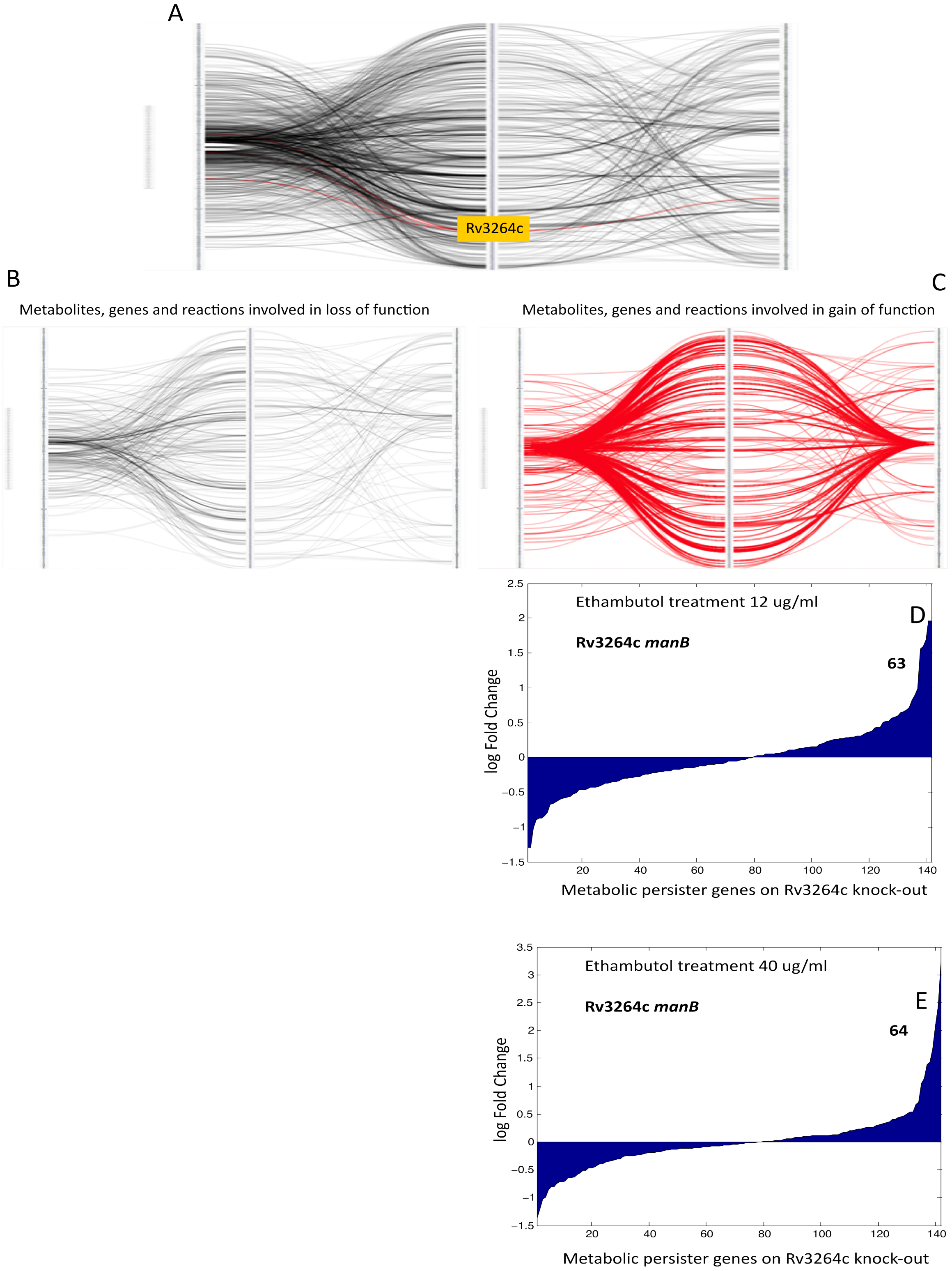

11 Table - S20 Metabolic Persister Reactions, Genes and Pathway on Rv3264c Deletion

Table 6: Metabolic persister reactions, gene and pathways on Rv3264c deletion

Reaction	Description	Formula	Genes	Pathways
ACKr	acetate kinase	ac[c] + atp[c] -> actp[c] + adp[c]	Rv0409	Pyruvate Metabolism
ACODA	acetylornithine deacetylase	acorn[c] + h2o[c] -> ac[c] + orn[c]		Other Amino Acid Metabolism
ADK1	adenylate kinase	amp[c] + atp[c] <=> 2 adp[c]	Rv0733	Purine Metabolism
ADK2	adenylate kinase Inorganic triphosphate	amp[c] + pppi[c] <=> adp[c] + ppi[c]	Rv0733	Purine Metabolism
ADK4	adentylate kinase ITP	amp[c] + itp[c] <=> adp[c] + idp[c]	Rv0733	Nucleotide Sugar Metabolism
ARGt5r	L arginine transport via diffusion	arg-L[e] <=> arg-L[c]	Rv2320c	Transport
ASPO5	L aspartate oxidase	asp-L[c] + fum[c] -> h[c] + iasp[c] + succ[c]	Rv1595	Alanine and Aspartate Metabolism
AST	Arginine succinyltransferase	arg-L[c] + succoa[c] -> coa[c] + h[c] + sucarg[c]		Arginine and Proline Metabolism
CAT	catalase	2 h2o2[c] -> 2 h2o[c] + o2[c]	Rv1908c	Redox Metabolism
CO2t	CO2 transporter via diffusion	co2[e] <=> co2[c]		Transport
CODH2r	carbon monoxide dehydrogenase acetyl CoA synthase 2	co[c] + 2 fdox[c] + h2o[c] <=> co2[c] + 2 fdred[c] + 2 h[c]	Rv0373c Rv0374c Rv0375c	Redox Metabolism
CODH3	carbon monoxide dehydrogenase acetyl CoA synthase 2	adfdOX[c] + 2 co[c] + 2 h2o[c] -> adfdRD[c] + 2 co2[c] + 2 h[c]	Rv0373c Rv0374c Rv0375c	Redox Metabolism
DCTPD2	dCTP deaminase	ctp[c] + h2o[c] + h[c] -> nh4[c] + utp[c]	Rv0321	Pyrimidine Metabolism
DHORD2	dihydoorotic acid dehydrogenase quinone8	dhor-S[c] + q8[c] -> orot[c] + q8h2[c]	Rv2139	Pyrimidine Metabolism
DHORD3	dihydroorotic acid dehydrogenase menaquinone 6	dhor-S[c] + mqn6[c] <=> mql6[c] + orot[c]	Rv2139	Pyrimidine Metabolism

Continued on next page

Table 6 – continued from previous page

Reaction	Description	Formula	Genes	Pathways
ECHH-0	Ech hydrogenase no electron transport	2 fdox[c] + h2[c] <=> 2 fdred[c] + 2 h[c]		Miscellaneous
ETHAt	ethanolamine transport via diffusion extracellular	etha[e] <=> etha[c]		Transport
EX-arg-L(e)	L Arginine exchange	arg-L[e] <=>		Exchange
EX-etha(e)	Ethanolamine exchange	etha[e] <=>		Exchange
EX-gly(e)	Glycine exchange	gly[e] <=>		Exchange
EX-k(e)	K exchange	k[e] <=>		Exchange
EX-mal-L(e)	L Malate exchange	mal-L[e] <=>		Exchange
EX-na1(e)	Sodium exchange	na1[e] <=>		Exchange
EX-ser-L(e)	L Serine exchange	ser-L[e] <=>		Exchange
FACOAL140	fatty acid CoA ligase tetradecanoate	atp[c] + coa[c] + ttdca[c] <=> amp[c] + ppi[c] + tdcoa[c]	Rv1529 Rv3826	Fatty Acid Metabolism
FACOAL161	fatty acid CoA ligase hexadecenoate	atp[c] + coa[c] + hdcea[c] <=> amp[c] + hdcoa[c] + ppi[c]	Rv1529 Rv3826	Fatty Acid Metabolism
FAO1	Fatty acid oxidation tetradecanoate	atp[c] + 7 coa[c] + 6 fad[c] + 6 h2o[c] + 6 nad[c] + ttdca[c] -> 7 accoa[c] + amp[c] + 6 fadh2[c] + 6 h[c] + 6 nadh[c] + ppi[c]	Rv0468 Rv0859 Rv0860 Rv1144 Rv1323	Fatty Acid Metabolism
FORMCOAL	formyl coa CoA ligase	atp[c] + coa[c] + for[c] <=> amp[c] + forcoa[c] + ppi[c]		Redox Metabolism
FRD	fumarate reductase	fadh2[c] + fum[c] -> fad[c] + succ[c]	Rv1552 Rv1553 Rv1554 Rv1555	Citric Acid Cycle
FRD2	fumarate reductase	fum[c] + mql8[c] -> mqn8[c] + succ[c]	Rv0247c Rv0248c Rv3316 Rv3317 Rv3318 Rv3319	Citric Acid Cycle
FRD5	fumarate reductase	fum[c] + mql6[c] <=> mqn6[c] + succ[c]	Rv1552 Rv1553 Rv1554 Rv1555	Citric Acid Cycle
G3PAT160	glycerol 3 phosphate acyltransferase C160	glyc3p[c] + palmACP[c] -> 1hdecg3p[c] + ACP[c]	Rv1551	Fatty Acid Metabolism
G6PDH2	glucose 6 phosphate dehydrogenase	g6p[c] + nadp[c] -> 6pgl[c] + 3 h[c] + nadph[c]	Rv1121 Rv1447c	Pentose Phosphate Pathway
GLYt2r	glycine reversible transport via proton symport	gly[e] + h[e] <=> gly[c] + h[c]	Rv1704c	Transport

Continued on next page

Table 6 – continued from previous page

Reaction	Description	Formula	Genes	Pathways
GPDDA2	Glycerophosphodiester phosphodiesterase Glycerophospho-ethanolamine	g3pe[c] + h2o[c] -> etha[c] + glyc3p[c] + h[c]	Rv0317c Rv2277c Rv3842c	Fatty Acid Metabolism
GTHOr	glutathione oxidoreductase	gthox[c] + h[c] + nadph[c] <=> 2 gthrd[c] + nadp[c]	Rv2855	Glutamate Metabolism
GTPDPK	GTP diphosphokinase	atp[c] + gtp[c] -> amp[c] + gdptp[c] + h[c]	Rv2583c	Purine Metabolism
HSDy	homoserine dehydrogenase NADPH	hom-L[c] + nadp[c] <=> aspsa[c] + h[c] + nadph[c]	Rv1294	Glycine Serine and Threonine Metabolism
Kt3r	potassium proton antiporter	h[e] + k[c] <=> h[c] + k[e]	Rv2691 Rv2692	Transport
LPLIPAL2E160	Lysophospholipase L2 2 acylglycerophospho-ethanolamine n C160	2agpe160[c] + h2o[c] -> g3pe[c] + h[c] + hdca[c]	Rv0183	Fatty Acid Metabolism
L-LACD2	L Lactate dehydrogenase ubiquinone	lac-L[c] + q8[c] -> pyr[c] + q8h2[c]	Rv1872c	Pyruvate Metabolism
MALt2r	L malate reversible transport via proton symport	h[e] + mal-L[e] <=> h[c] + mal-L[c]	Rv2443	Transport
ME1	malic enzyme NAD	mal-L[c] + nad[c] -> co2[c] + nadh[c] + pyr[c]	Rv2332	Pyruvate Metabolism
MYCTR	mycothiol reductase nadp dependent	2 msh[c] + nadp[c] <=> h[c] + mssg[c] + nadph[c]	Rv2855	Redox Metabolism
MYCTR2	mycothiol reductase glutathione exchange	gthox[c] + 2 msh[c] <=> 2 gthrd[c] + mssg[c]	Rv2855	Redox Metabolism
NADH2r	NADH dehydrogenase	h[c] + nadh[c] + q[c] <=> nad[c] + qh2[c]	Rv0082 Rv3145 Rv3146 Rv3147 Rv3148 Rv3149 Rv3150 Rv3151 Rv3152 Rv3153 Rv3154 Rv3155 Rv3156 Rv3157 Rv3158	Redox Metabolism
NADS2	NAD synthase glutamine hydrolysing	atp[c] + dnad[c] + gln-L[c] + h2o[c] -> amp[c] + glu-L[c] + h[c] + nad[c] + ppi[c]	Rv2438c	Glutamate Metabolism

Continued on next page

Table 6 – continued from previous page

Reaction	Description	Formula	Genes	Pathways
NAKtr	potassium sodium antiporter	k[c] + na1[e] <=> k[e] + na1[c]	Rv3236c	Transport
NAt3-1	sodium proton antiporter HNA is 11	h[e] + na1[c] <=> h[c] + na1[e]	Rv2287	Transport
NDPK9	nucleoside diphosphate kinase ATPIDP	atp[c] + idp[c] <=> adp[c] + itp[c]	Rv2445c	Pyrimidine Metabolism
NNAT	nicotinate nucleotide adenylyltransferase	atp[c] + h[c] + nicrnt[c] -> dnad[c] + ppi[c]	Rv2421c	Pantothenate and CoA Metabolism
NNDPR	nicotinate nucleotide diphosphorylase carboxylating	2 h[c] + prpp[c] + quln[c] -> co2[c] + nicrnt[c] + ppi[c]	Rv1596	Pantothenate and CoA Metabolism
ORNt	ornithine transport via diffusion extracellular to periplasm	orn[e] <=> orn[c]	Rv2320c	Transport
OXGDC	2 oxoglutarate decarboxylase	akg[c] + h[c] -> co2[c] + sucsal[c]	Rv1248c	Citric Acid Cycle
PGL	6 phosphogluconolactonase	6pgl[c] + h2o[c] -> 6pgc[c] + h[c]	Rv1445c	Pentose Phosphate Pathway
PHTO	phthiocol oxygenase	2 o2[c] + phthclh2[c] -> 2 h[c] + 2 o2s[c] + phthcl[c]		Ubiquinone Metabolism
PLIPA1E160	Phospholipase A1 phosphatidylethanolamine n C160	h2o[c] + pe160[c] -> 2agpe160[c] + h[c] + hdca[c]		Fatty Acid Metabolism
PPDK	Pyruvate phosphate dikinase	atp[c] + pi[c] + pyr[c] -> amp[c] + h[c] + pep[c] + ppi[c]	Rv1127c	Pyruvate Metabolism
PPK2	polyphosphate kinase	atp[c] + ppi[c] -> adp[c] + pppi[c]	Rv2984	Purine Metabolism
PTAr	phosphotransacetylase	accoa[c] + pi[c] <=> actp[c] + coa[c]	Rv0408 Rv3914	Pyruvate Metabolism
QRr	Quinone reductase	h[c] + nadph[c] + q[c] <=> nadp[c] + qh2[c]	Rv3777	Redox Metabolism
QULNS	quinolinate synthase	dhap[c] + iasp[c] -> 2 h2o[c] + pi[c] + quln[c]	Rv1594	Pantothenate and CoA Metabolism
SERt2r	L serine reversible transport via proton symport	h[e] + ser-L[e] <=> h[c] + ser-L[c]	Rv1704c	Transport
SGDS	Succinylglutamate desuccinylase	h2o[c] + sucglu[c] -> glu-L[c] + succ[c]		Arginine and Proline Metabolism

Continued on next page

Table 6 – continued from previous page

Reaction	Description	Formula	Genes	Pathways
SGSAD	Succinylglutamic semialdehyde dehydrogenase	h2o[c] + nad[c] + sucgsa[c] -> 2 h[c] + nadh[c] + sucglu[c]		Arginine and Proline Metabolism
SOTA	Succinylornithine transaminase	akg[c] + sucorn[c] -> glu-L[c] + sucgsa[c]		Arginine and Proline Metabolism
SPODM	superoxide dismutase	2 h[c] + 2 o2s[c] -> h2o2[c] + o2[c]	Rv0432 Rv1908c Rv3846	Cofactor Metabolism
SSALy	succinate semialdehyde dehydrogenase NADP	h2o[c] + nadp[c] + sucsal[c] -> 2 h[c] + nadph[c] + succ[c]	Rv0234c Rv1731	Glutamate Metabolism
THRS	threonine synthase	h2o[c] + phom[c] -> pi[c] + thr-L[c]	Rv1295	Glycine Serine and Threonine Metabolism
XYLK	xylulokinase	atp[c] + xylu-D[c] -> adp[c] + h[c] + xu5p-D[c]	Rv0729	Sugar Metabolism
PPGPP	PPGPP-3-pyrophosphohydrolase	gdpdp[c] + h2o[c] -> gdp[c] + ppi[c]		Purine Metabolism

Continued on next page

Table 6 – continued from previous page

Reaction	Description	Formula	Genes	Pathways
FAD-8	acyl-CoA dehydrogenase fadE1-36 AND enoyl-CoA hydratase/isomerase echA1-21 AND 3-hydroxyacyl-CoA dehydrogenase fadB2-5 AND acetyl-CoA C-acetyltransferase fadA2-13	tdcoa[c] + fad[c] + nad[c] + coa[c] -> dodcoa[c] + fadh2[c] + nadh[c] + accoa[c]	Rv0131c Rv0154c Rv0215c Rv0231 Rv0244c Rv0271c Rv0400c Rv0752c Rv0873 Rv0972c Rv0975c Rv1346 Rv1467c Rv1679 Rv1933c Rv1934c Rv2724c Rv2789c Rv3061c Rv3139 Rv3140 Rv3274c Rv3504 Rv3505 Rv3543c Rv3544c Rv3560c Rv3562 Rv3563 Rv3564 Rv3573c Rv3761c Rv3797 Rv0672 Rv2500c Rv0222 Rv0456c Rv0632c Rv0673 Rv0675 Rv0971c Rv1070c Rv1071c Rv1141c Rv1142c Rv1472 Rv1935c Rv2486 Rv2679 Rv2831 Rv3039c Rv3516 Rv3550 Rv3774 Rv0905 Rv3374 Rv3373 Rv0468 Rv1715 Rv1912c Rv3141 Rv0243 Rv1074c Rv3546 Rv3556c Rv1323 Rv0859	Beta oxidation of fatty acids

Continued on next page

Table 6 – continued from previous page

Reaction	Description	Formula	Genes	Pathways
FAD-9	acyl-CoA dehydrogenase fadE1-36 AND enoyl-CoA hydratase/isomerase echA1-21 AND 3-hydroxyacyl-CoA dehydrogenase fadB2-5 AND acetyl-CoA C-acetyltransferase fadA2-14	dodcoa[c] + fad[c] + nad[c] + coa[c] -> decoa[c] + fadh2[c] + nadh[c] + accoa[c]	Rv0131c Rv0154c Rv0215c Rv0231 Rv0244c Rv0271c Rv0400c Rv0752c Rv0873 Rv0972c Rv0975c Rv1346 Rv1467c Rv1679 Rv1933c Rv1934c Rv2724c Rv2789c Rv3061c Rv3139 Rv3140 Rv3274c Rv3504 Rv3505 Rv3543c Rv3544c Rv3560c Rv3562 Rv3563 Rv3564 Rv3573c Rv3761c Rv3797 Rv0672 Rv2500c Rv0222 Rv0456c Rv0632c Rv0673 Rv0675 Rv0971c Rv1070c Rv1071c Rv1141c Rv1142c Rv1472 Rv1935c Rv2486 Rv2679 Rv2831 Rv3039c Rv3516 Rv3550 Rv3774 Rv0905 Rv3374 Rv3373 Rv0468 Rv1715 Rv1912c Rv3141 Rv0243 Rv1074c Rv3546 Rv3556c Rv1323 Rv0859	Beta oxidation of fatty acids

Continued on next page

Table 6 – continued from previous page

Reaction	Description	Formula	Genes	Pathways
FAD-15	acyl-CoA dehydrogenase fadE1-36 AND enoyl-CoA hydratase/isomerase echA1-21 AND 3-hydroxyacyl-CoA dehydrogenase fadB2-5 AND acetyl-CoA C-acetyltransferase fadA2-20	hdcoa[c] + fad[c] + nad[c] + coa[c] -> c5tetdcoa[c] + fadh2[c] + nadh[c] + accoa[c]	Rv0131c Rv0154c Rv0215c Rv0231 Rv0244c Rv0271c Rv0400c Rv0752c Rv0873 Rv0972c Rv0975c Rv1346 Rv1467c Rv1679 Rv1933c Rv1934c Rv2724c Rv2789c Rv3061c Rv3139 Rv3140 Rv3274c Rv3504 Rv3505 Rv3543c Rv3544c Rv3560c Rv3562 Rv3563 Rv3564 Rv3573c Rv3761c Rv3797 Rv0672 Rv2500c Rv0222 Rv0456c Rv0632c Rv0673 Rv0675 Rv0971c Rv1070c Rv1071c Rv1141c Rv1142c Rv1472 Rv1935c Rv2486 Rv2679 Rv2831 Rv3039c Rv3516 Rv3550 Rv3774 Rv0905 Rv3374 Rv3373 Rv0468 Rv1715 Rv1912c Rv3141 Rv0243 Rv1074c Rv3546 Rv3556c Rv1323 Rv0859	Beta oxidation of unsaturated fatty acids

Continued on next page

Table 6 – continued from previous page

Reaction	Description	Formula	Genes	Pathways
FAD-16	acyl-CoA dehydrogenase fadE1-36 AND enoyl-CoA hydratase/isomerase echA1-21 AND 3-hydroxyacyl-CoA dehydrogenase fadB2-5 AND acetyl-CoA C-acetyltransferase fadA2-21	c5tetdcoa[c] + fad[c] + nad[c] + coa[c] -> c3dodcoa[c] + fadh2[c] + nadh[c] + accoa[c]	Rv0131c Rv0154c Rv0215c Rv0231 Rv0244c Rv0271c Rv0400c Rv0752c Rv0873 Rv0972c Rv0975c Rv1346 Rv1467c Rv1679 Rv1933c Rv1934c Rv2724c Rv2789c Rv3061c Rv3139 Rv3140 Rv3274c Rv3504 Rv3505 Rv3543c Rv3544c Rv3560c Rv3562 Rv3563 Rv3564 Rv3573c Rv3761c Rv3797 Rv0672 Rv2500c Rv0222 Rv0456c Rv0632c Rv0673 Rv0675 Rv0971c Rv1070c Rv1071c Rv1141c Rv1142c Rv1472 Rv1935c Rv2486 Rv2679 Rv2831 Rv3039c Rv3516 Rv3550 Rv3774 Rv0905 Rv3374 Rv3373 Rv0468 Rv1715 Rv1912c Rv3141 Rv0243 Rv1074c Rv3546 Rv3556c Rv1323 Rv0859	Beta oxidation of unsaturated fatty acids

Continued on next page

Table 6 – continued from previous page

Reaction	Description	Formula	Genes	Pathways
FAD-17	acyl-CoA dehydrogenase fadE1-36 AND enoyl-CoA hydratase/isomerase echA1-21 AND 3-hydroxyacyl-CoA dehydrogenase fadB2-5 AND acetyl-CoA C-acetyltransferase fadA2-22	c3dodcoa[c] -> t2dodcoa[c]	Rv0131c Rv0154c Rv0215c Rv0231 Rv0244c Rv0271c Rv0400c Rv0752c Rv0873 Rv0972c Rv0975c Rv1346 Rv1467c Rv1679 Rv1933c Rv1934c Rv2724c Rv2789c Rv3061c Rv3139 Rv3140 Rv3274c Rv3504 Rv3505 Rv3543c Rv3544c Rv3560c Rv3562 Rv3563 Rv3564 Rv3573c Rv3761c Rv3797 Rv0672 Rv2500c Rv0222 Rv0456c Rv0632c Rv0673 Rv0675 Rv0971c Rv1070c Rv1071c Rv1141c Rv1142c Rv1472 Rv1935c Rv2486 Rv2679 Rv2831 Rv3039c Rv3516 Rv3550 Rv3774 Rv0905 Rv3374 Rv3373 Rv0468 Rv1715 Rv1912c Rv3141 Rv0243 Rv1074c Rv3546 Rv3556c Rv1323 Rv0859	Beta oxidation of unsaturated fatty acids

Continued on next page

Table 6 – continued from previous page

Reaction	Description	Formula	Genes	Pathways
FAD-19	acyl-CoA dehydrogenase fadE1-36 AND enoyl-CoA hydratase/isomerase echA1-21 AND 3-hydroxyacyl-CoA dehydrogenase fadB2-5 AND acetyl-CoA C-acetyltransferase fadA2-24	d2hdodcoa[c] + nad[c] -> dodcoa[c] + nadh[c]	Rv0131c Rv0154c Rv0215c Rv0231 Rv0244c Rv0271c Rv0400c Rv0752c Rv0873 Rv0972c Rv0975c Rv1346 Rv1467c Rv1679 Rv1933c Rv1934c Rv2724c Rv2789c Rv3061c Rv3139 Rv3140 Rv3274c Rv3504 Rv3505 Rv3543c Rv3544c Rv3560c Rv3562 Rv3563 Rv3564 Rv3573c Rv3761c Rv3797 Rv0672 Rv2500c Rv0222 Rv0456c Rv0632c Rv0673 Rv0675 Rv0971c Rv1070c Rv1071c Rv1141c Rv1142c Rv1472 Rv1935c Rv2486 Rv2679 Rv2831 Rv3039c Rv3516 Rv3550 Rv3774 Rv0905 Rv3374 Rv3373 Rv0468 Rv1715 Rv1912c Rv3141 Rv0243 Rv1074c Rv3546 Rv3556c Rv1323 Rv0859	Beta oxidation of unsaturated fatty acids
ALDR	Aldolase reductase	arab-D[c] + nadph[c] + h[c] -> arabl-D[c] + nadp[c]		Arabinogalactan Synthesis
AL4D	D-arabinitol 4-dehydrogenase	arabl-D[c] + nad[c] -> xylu-D[c] + nadh[c] + h[c]	Rv1928c	Arabinogalactan Synthesis

12 Fig. S6 - Spindle Map Rv3266c

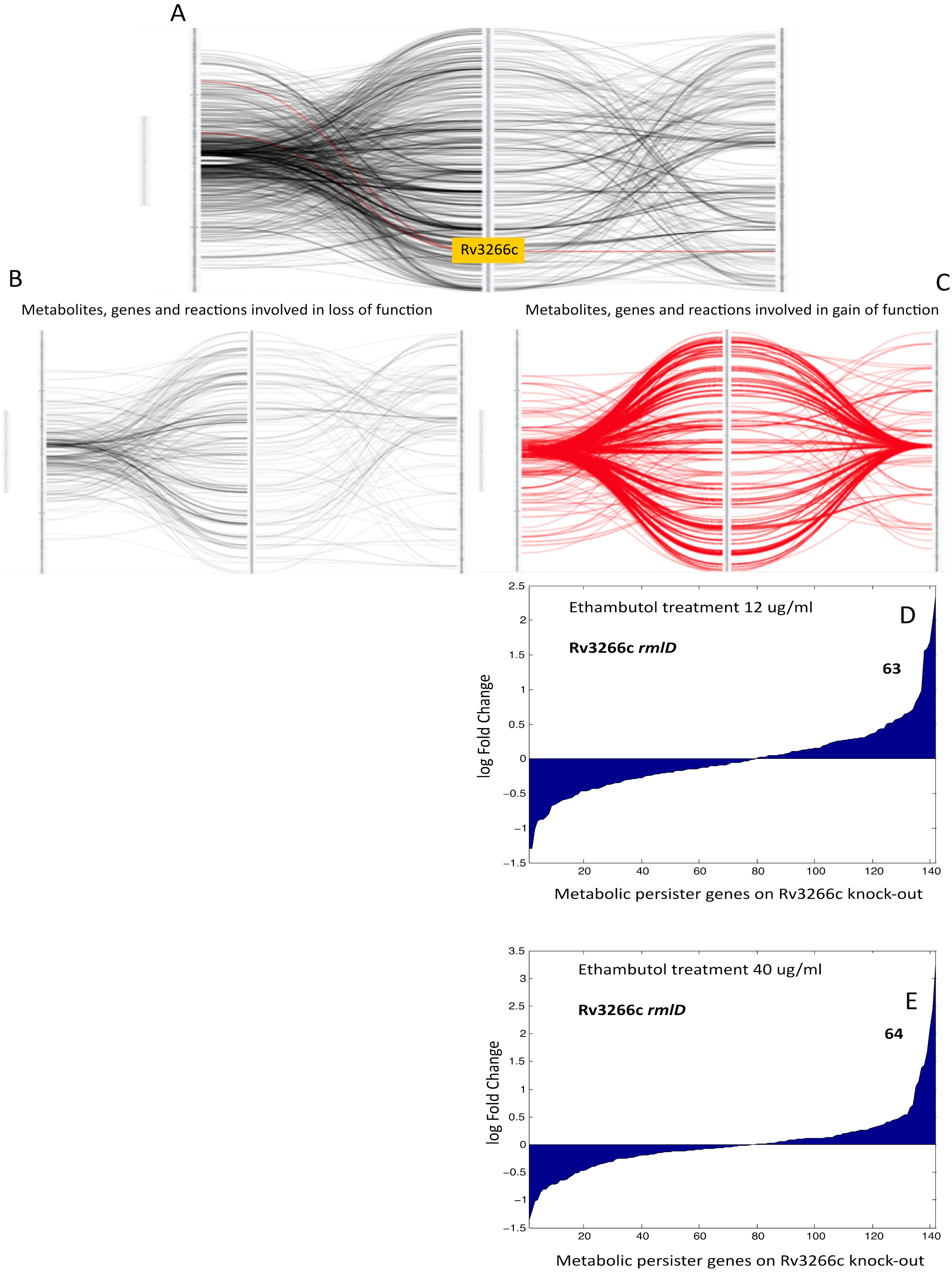

13 Table - S21 Metabolic Persister Reactions, Genes and Pathway on Rv3266c Deletion

Table 7: Metabolic persister reactions, gene and pathways on Rv3266c deletion

Reaction	Description	Formula	Genes	Pathways
ACKr	acetate kinase	ac[c] + atp[c] -> actp[c] + adp[c]	Rv0409	Pyruvate Metabolism
ADK1	adenylate kinase	amp[c] + atp[c] <=> 2 adp[c]	Rv0733	Purine Metabolism
ADK2	adenylate kinase Inorganic triphosphate	amp[c] + pppi[c] <=> adp[c] + ppi[c]	Rv0733	Purine Metabolism
ADK4	adentylate kinase ITP	amp[c] + itp[c] <=> adp[c] + idp[c]	Rv0733	Nucleotide Sugar Metabolism
ARGt5r	L arginine transport via diffusion	arg-L[e] <=> arg-L[c]	Rv2320c	Transport
ASPO5	L aspartate oxidase	asp-L[c] + fum[c] -> h[c] + iasp[c] + succ[c]	Rv1595	Alanine and Aspartate Metabolism
AST	Arginine succinyltransferase	arg-L[c] + succoa[c] -> coa[c] + h[c] + sucarg[c]		Arginine and Proline Metabolism
CAT	catalase	2 h2o2[c] -> 2 h2o[c] + o2[c]	Rv1908c	Redox Metabolism
CO2t	CO2 transporter via diffusion	co2[e] <=> co2[c]		Transport
CODH2r	carbon monoxide dehydrogenase acetyl CoA synthase 2	co[c] + 2 fdox[c] + h2o[c] <=> co2[c] + 2 fdred[c] + 2 h[c]	Rv0373c Rv0374c Rv0375c	Redox Metabolism
CODH3	carbon monoxide dehydrogenase acetyl CoA synthase 2	adfdOX[c] + 2 co[c] + 2 h2o[c] -> adfdRD[c] + 2 co2[c] + 2 h[c]	Rv0373c Rv0374c Rv0375c	Redox Metabolism
DCTPD2	dCTP deaminase	ctp[c] + h2o[c] + h[c] -> nh4[c] + utp[c]	Rv0321	Pyrimidine Metabolism
DHORD2	dihydoorotic acid dehydrogenase quinone8	dhor-S[c] + q8[c] -> orot[c] + q8h2[c]	Rv2139	Pyrimidine Metabolism
DHORD3	dihydroorotic acid dehydrogenase menaquinone 6	dhor-S[c] + mqn6[c] <=> mql6[c] + orot[c]	Rv2139	Pyrimidine Metabolism
ECHH-0	Ech hydrogenase no electron transport	2 fdox[c] + h2[c] <=> 2 fdred[c] + 2 h[c]		Miscellaneous

Continued on next page

Table 7 – continued from previous page

Reaction	Description	Formula	Genes	Pathways
ETHAt	ethanolamine transport via diffusion extracellular	etha[e] <=> etha[c]		Transport
EX-arg-L(e)	L Arginine exchange	arg-L[e] <=>		Exchange
EX-etha(e)	Ethanolamine exchange	etha[e] <=>		Exchange
EX-gly(e)	Glycine exchange	gly[e] <=>		Exchange
EX-k(e)	K exchange	k[e] <=>		Exchange
EX-mal-L(e)	L Malate exchange	mal-L[e] <=>		Exchange
EX-na1(e)	Sodium exchange	na1[e] <=>		Exchange
EX-ser-L(e)	L Serine exchange	ser-L[e] <=>		Exchange
FACOAL140	fatty acid CoA ligase tetradecanoate	atp[c] + coa[c] + ttdca[c] <=> amp[c] + ppi[c] + tdcoa[c]	Rv1529 Rv3826	Fatty Acid Metabolism
FACOAL161	fatty acid CoA ligase hexadecenoate	atp[c] + coa[c] + hdcea[c] <=> amp[c] + hdcoa[c] + ppi[c]	Rv1529 Rv3826	Fatty Acid Metabolism
FAO1	Fatty acid oxidation tetradecanoate	atp[c] + 7 coa[c] + 6 fad[c] + 6 h2o[c] + 6 nad[c] + ttdca[c] -> 7 accoa[c] + amp[c] + 6 fadh2[c] + 6 h[c] + 6 nadh[c] + ppi[c]	Rv0468 Rv0859 Rv0860 Rv1144 Rv1323	Fatty Acid Metabolism
FORMCOAL	formyl coa CoA ligase	atp[c] + coa[c] + for[c] <=> amp[c] + forcoa[c] + ppi[c]		Redox Metabolism
FRD	fumarate reductase	fadh2[c] + fum[c] -> fad[c] + succ[c]	Rv1552 Rv1553 Rv1554 Rv1555	Citric Acid Cycle
FRD2	fumarate reductase	fum[c] + mql8[c] -> mqn8[c] + succ[c]	Rv0247c Rv0248c Rv3316 Rv3317 Rv3318 Rv3319	Citric Acid Cycle
FRD5	fumarate reductase	fum[c] + mql6[c] <=> mqn6[c] + succ[c]	Rv1552 Rv1553 Rv1554 Rv1555	Citric Acid Cycle
G3PAT160	glycerol 3 phosphate acyltransferase C160	glyc3p[c] + palmACP[c] -> 1hdecg3p[c] + ACP[c]	Rv1551	Fatty Acid Metabolism
G6PDH2	glucose 6 phosphate dehydrogenase	g6p[c] + nadp[c] -> 6pgl[c] + 3 h[c] + nadph[c]	Rv1121 Rv1447c	Pentose Phosphate Pathway
GLYt2r	glycine reversible transport via proton symport	gly[e] + h[e] <=> gly[c] + h[c]	Rv1704c	Transport

Continued on next page

Table 7 – continued from previous page

Reaction	Description	Formula	Genes	Pathways
GPDDA2	Glycerophosphodiester phosphodiesterase Glycerophospho-ethanolamine	g3pe[c] + h2o[c] -> etha[c] + glyc3p[c] + h[c]	Rv0317c Rv2277c Rv3842c	Fatty Acid Metabolism
GTHOr	glutathione oxidoreductase	gthox[c] + h[c] + nadph[c] <=> 2 gthrd[c] + nadp[c]	Rv2855	Glutamate Metabolism
GTPDPK	GTP diphosphokinase	atp[c] + gtp[c] -> amp[c] + gdptp[c] + h[c]	Rv2583c	Purine Metabolism
HSDy	homoserine dehydrogenase NADPH	hom-L[c] + nadp[c] <=> aspsa[c] + h[c] + nadph[c]	Rv1294	Glycine Serine and Threonine Metabolism
Kt3r	potassium proton antiporter	h[e] + k[c] <=> h[c] + k[e]	Rv2691 Rv2692	Transport
LPLIPAL2E160	Lysophospholipase L2 2 acylglycerophospho-ethanolamine n C160	2agpe160[c] + h2o[c] -> g3pe[c] + h[c] + hdca[c]	Rv0183	Fatty Acid Metabolism
L-LACD2	L Lactate dehydrogenase ubiquinone	lac-L[c] + q8[c] -> pyr[c] + q8h2[c]	Rv1872c	Pyruvate Metabolism
MALt2r	L malate reversible transport via proton symport	h[e] + mal-L[e] <=> h[c] + mal-L[c]	Rv2443	Transport
ME1	malic enzyme NAD	mal-L[c] + nad[c] -> co2[c] + nadh[c] + pyr[c]	Rv2332	Pyruvate Metabolism
MYCTR	mycothiol reductase nadp dependent	2 msh[c] + nadp[c] <=> h[c] + mssg[c] + nadph[c]	Rv2855	Redox Metabolism
MYCTR2	mycothiol reductase glutathione exchange	gthox[c] + 2 msh[c] <=> 2 gthrd[c] + mssg[c]	Rv2855	Redox Metabolism
NADH2r	NADH dehydrogenase	h[c] + nadh[c] + q[c] <=> nad[c] + qh2[c]	Rv0082 Rv3145 Rv3146 Rv3147 Rv3148 Rv3149 Rv3150 Rv3151 Rv3152 Rv3153 Rv3154 Rv3155 Rv3156 Rv3157 Rv3158	Redox Metabolism
NADS2	NAD synthase glutamine hydrolysing	atp[c] + dnad[c] + gln-L[c] + h2o[c] -> amp[c] + glu-L[c] + h[c] + nad[c] + ppi[c]	Rv2438c	Glutamate Metabolism

Continued on next page

Table 7 – continued from previous page

Reaction	Description	Formula	Genes	Pathways
NAKtr	potassium sodium antiporter	k[c] + na1[e] <=> k[e] + na1[c]	Rv3236c	Transport
NAt3-1	sodium proton antiporter HNA is 11	h[e] + na1[c] <=> h[c] + na1[e]	Rv2287	Transport
NDPK9	nucleoside diphosphate kinase ATPIDP	atp[c] + idp[c] <=> adp[c] + itp[c]	Rv2445c	Pyrimidine Metabolism
NNAT	nicotinate nucleotide adenylyltransferase	atp[c] + h[c] + nicrnt[c] -> dnad[c] + ppi[c]	Rv2421c	Pantothenate and CoA Metabolism
NNDPR	nicotinate nucleotide diphosphorylase carboxylating	2 h[c] + prpp[c] + quln[c] -> co2[c] + nicrnt[c] + ppi[c]	Rv1596	Pantothenate and CoA Metabolism
ORNt	ornithine transport via diffusion extracellular to periplasm	orn[e] <=> orn[c]	Rv2320c	Transport
OXGDC	2 oxoglutarate decarboxylase	akg[c] + h[c] -> co2[c] + sucsal[c]	Rv1248c	Citric Acid Cycle
PGL	6 phosphogluconolactonase	6pgl[c] + h2o[c] -> 6pgc[c] + h[c]	Rv1445c	Pentose Phosphate Pathway
PHTO	phthiocol oxygenase	2 o2[c] + phthclh2[c] -> 2 h[c] + 2 o2s[c] + phthcl[c]		Ubiquinone Metabolism
PLIPA1E160	Phospholipase A1 phosphatidylethanolamine n C160	h2o[c] + pe160[c] -> 2agpe160[c] + h[c] + hdca[c]		Fatty Acid Metabolism
PPDK	Pyruvate phosphate dikinase	atp[c] + pi[c] + pyr[c] -> amp[c] + h[c] + pep[c] + ppi[c]	Rv1127c	Pyruvate Metabolism
PPK2	polyphosphate kinase	atp[c] + ppi[c] -> adp[c] + pppi[c]	Rv2984	Purine Metabolism
PTAr	phosphotransacetylase	accoa[c] + pi[c] <=> actp[c] + coa[c]	Rv0408 Rv3914	Pyruvate Metabolism
QRr	Quinone reductase	h[c] + nadph[c] + q[c] <=> nadp[c] + qh2[c]	Rv3777	Redox Metabolism
QULNS	quinolinate synthase	dhap[c] + iasp[c] -> 2 h2o[c] + pi[c] + quln[c]	Rv1594	Pantothenate and CoA Metabolism
SERt2r	L serine reversible transport via proton symport	h[e] + ser-L[e] <=> h[c] + ser-L[c]	Rv1704c	Transport
SGDS	Succinylglutamate desuccinylase	h2o[c] + sucglu[c] -> glu-L[c] + succ[c]		Arginine and Proline Metabolism

Continued on next page

Table 7 – continued from previous page

Reaction	Description	Formula	Genes	Pathways
SGSAD	Succinylglutamic semialdehyde dehydrogenase	h2o[c] + nad[c] + sucgsa[c] -> 2 h[c] + nadh[c] + sucglu[c]		Arginine and Proline Metabolism
SOTA	Succinylornithine transaminase	akg[c] + sucorn[c] -> glu-L[c] + sucgsa[c]		Arginine and Proline Metabolism
SPODM	superoxide dismutase	2 h[c] + 2 o2s[c] -> h2o2[c] + o2[c]	Rv0432 Rv1908c Rv3846	Cofactor Metabolism
SSALy	succinate semialdehyde dehydrogenase NADP	h2o[c] + nadp[c] + sucsal[c] -> 2 h[c] + nadph[c] + succ[c]	Rv0234c Rv1731	Glutamate Metabolism
XYLK	xylulokinase	atp[c] + xylu-D[c] -> adp[c] + h[c] + xu5p-D[c]	Rv0729	Sugar Metabolism
PPGPP	PPGPP-3-pyrophosphohydrolase	gdpdp[c] + h2o[c] -> gdp[c] + ppi[c]		Purine Metabolism

Continued on next page

Table 7 – continued from previous page

Reaction	Description	Formula	Genes	Pathways
FAD-8	acyl-CoA dehydrogenase fadE1-36 AND enoyl-CoA hydratase/isomerase echA1-21 AND 3-hydroxyacyl-CoA dehydrogenase fadB2-5 AND acetyl-CoA C-acetyltransferase fadA2-13	tdcoa[c] + fad[c] + nad[c] + coa[c] -> dodcoa[c] + fadh2[c] + nadh[c] + accoa[c]	Rv0131c Rv0154c Rv0215c Rv0231 Rv0244c Rv0271c Rv0400c Rv0752c Rv0873 Rv0972c Rv0975c Rv1346 Rv1467c Rv1679 Rv1933c Rv1934c Rv2724c Rv2789c Rv3061c Rv3139 Rv3140 Rv3274c Rv3504 Rv3505 Rv3543c Rv3544c Rv3560c Rv3562 Rv3563 Rv3564 Rv3573c Rv3761c Rv3797 Rv0672 Rv2500c Rv0222 Rv0456c Rv0632c Rv0673 Rv0675 Rv0971c Rv1070c Rv1071c Rv1141c Rv1142c Rv1472 Rv1935c Rv2486 Rv2679 Rv2831 Rv3039c Rv3516 Rv3550 Rv3774 Rv0905 Rv3374 Rv3373 Rv0468 Rv1715 Rv1912c Rv3141 Rv0243 Rv1074c Rv3546 Rv3556c Rv1323 Rv0859	Beta oxidation of fatty acids

Continued on next page

Table 7 – continued from previous page

Reaction	Description	Formula	Genes	Pathways
FAD-9	acyl-CoA dehydrogenase fadE1-36 AND enoyl-CoA hydratase/isomerase echA1-21 AND 3-hydroxyacyl-CoA dehydrogenase fadB2-5 AND acetyl-CoA C-acetyltransferase fadA2-14	dodcoa[c] + fad[c] + nad[c] + coa[c] -> decoa[c] + fadh2[c] + nadh[c] + accoa[c]	Rv0131c Rv0154c Rv0215c Rv0231 Rv0244c Rv0271c Rv0400c Rv0752c Rv0873 Rv0972c Rv0975c Rv1346 Rv1467c Rv1679 Rv1933c Rv1934c Rv2724c Rv2789c Rv3061c Rv3139 Rv3140 Rv3274c Rv3504 Rv3505 Rv3543c Rv3544c Rv3560c Rv3562 Rv3563 Rv3564 Rv3573c Rv3761c Rv3797 Rv0672 Rv2500c Rv0222 Rv0456c Rv0632c Rv0673 Rv0675 Rv0971c Rv1070c Rv1071c Rv1141c Rv1142c Rv1472 Rv1935c Rv2486 Rv2679 Rv2831 Rv3039c Rv3516 Rv3550 Rv3774 Rv0905 Rv3374 Rv3373 Rv0468 Rv1715 Rv1912c Rv3141 Rv0243 Rv1074c Rv3546 Rv3556c Rv1323 Rv0859	Beta oxidation of fatty acids

Continued on next page

Table 7 – continued from previous page

Reaction	Description	Formula	Genes	Pathways
FAD-11	acyl-CoA dehydrogenase fadE1-36 AND enoyl-CoA hydratase/isomerase echA1-21 AND 3-hydroxyacyl-CoA dehydrogenase fadB2-5 AND acetyl-CoA C-acetyltransferase fadA2-16	occoa[c] + fad[c] + nad[c] + coa[c] -> hexcoa[c] + fadh2[c] + nadh[c] + accoa[c]	Rv0131c Rv0154c Rv0215c Rv0231 Rv0244c Rv0271c Rv0400c Rv0752c Rv0873 Rv0972c Rv0975c Rv1346 Rv1467c Rv1679 Rv1933c Rv1934c Rv2724c Rv2789c Rv3061c Rv3139 Rv3140 Rv3274c Rv3504 Rv3505 Rv3543c Rv3544c Rv3560c Rv3562 Rv3563 Rv3564 Rv3573c Rv3761c Rv3797 Rv0672 Rv2500c Rv0222 Rv0456c Rv0632c Rv0673 Rv0675 Rv0971c Rv1070c Rv1071c Rv1141c Rv1142c Rv1472 Rv1935c Rv2486 Rv2679 Rv2831 Rv3039c Rv3516 Rv3550 Rv3774 Rv0905 Rv3374 Rv3373 Rv0468 Rv1715 Rv1912c Rv3141 Rv0243 Rv1074c Rv3546 Rv3556c Rv1323 Rv0859	Beta oxidation of fatty acids

Continued on next page

Table 7 – continued from previous page

Reaction	Description	Formula	Genes	Pathways
FAD-15	acyl-CoA dehydrogenase fadE1-36 AND enoyl-CoA hydratase/isomerase echA1-21 AND 3-hydroxyacyl-CoA dehydrogenase fadB2-5 AND acetyl-CoA C-acetyltransferase fadA2-20	hdcoa[c] + fad[c] + nad[c] + coa[c] -> c5tetdcoa[c] + fadh2[c] + nadh[c] + accoa[c]	Rv0131c Rv0154c Rv0215c Rv0231 Rv0244c Rv0271c Rv0400c Rv0752c Rv0873 Rv0972c Rv0975c Rv1346 Rv1467c Rv1679 Rv1933c Rv1934c Rv2724c Rv2789c Rv3061c Rv3139 Rv3140 Rv3274c Rv3504 Rv3505 Rv3543c Rv3544c Rv3560c Rv3562 Rv3563 Rv3564 Rv3573c Rv3761c Rv3797 Rv0672 Rv2500c Rv0222 Rv0456c Rv0632c Rv0673 Rv0675 Rv0971c Rv1070c Rv1071c Rv1141c Rv1142c Rv1472 Rv1935c Rv2486 Rv2679 Rv2831 Rv3039c Rv3516 Rv3550 Rv3774 Rv0905 Rv3374 Rv3373 Rv0468 Rv1715 Rv1912c Rv3141 Rv0243 Rv1074c Rv3546 Rv3556c Rv1323 Rv0859	Beta oxidation of unsaturated fatty acids

Continued on next page

Table 7 – continued from previous page

Reaction	Description	Formula	Genes	Pathways
FAD-16	acyl-CoA dehydrogenase fadE1-36 AND enoyl-CoA hydratase/isomerase echA1-21 AND 3-hydroxyacyl-CoA dehydrogenase fadB2-5 AND acetyl-CoA C-acetyltransferase fadA2-21	c5tetdcoa[c] + fad[c] + nad[c] + coa[c] -> c3dodcoa[c] + fadh2[c] + nadh[c] + accoa[c]	Rv0131c Rv0154c Rv0215c Rv0231 Rv0244c Rv0271c Rv0400c Rv0752c Rv0873 Rv0972c Rv0975c Rv1346 Rv1467c Rv1679 Rv1933c Rv1934c Rv2724c Rv2789c Rv3061c Rv3139 Rv3140 Rv3274c Rv3504 Rv3505 Rv3543c Rv3544c Rv3560c Rv3562 Rv3563 Rv3564 Rv3573c Rv3761c Rv3797 Rv0672 Rv2500c Rv0222 Rv0456c Rv0632c Rv0673 Rv0675 Rv0971c Rv1070c Rv1071c Rv1141c Rv1142c Rv1472 Rv1935c Rv2486 Rv2679 Rv2831 Rv3039c Rv3516 Rv3550 Rv3774 Rv0905 Rv3374 Rv3373 Rv0468 Rv1715 Rv1912c Rv3141 Rv0243 Rv1074c Rv3546 Rv3556c Rv1323 Rv0859	Beta oxidation of unsaturated fatty acids

Continued on next page

Table 7 – continued from previous page

Reaction	Description	Formula	Genes	Pathways
FAD-17	acyl-CoA dehydrogenase fadE1-36 AND enoyl-CoA hydratase/isomerase echA1-21 AND 3-hydroxyacyl-CoA dehydrogenase fadB2-5 AND acetyl-CoA C-acetyltransferase fadA2-22	c3dodcoa[c] -> t2dodcoa[c]	Rv0131c Rv0154c Rv0215c Rv0231 Rv0244c Rv0271c Rv0400c Rv0752c Rv0873 Rv0972c Rv0975c Rv1346 Rv1467c Rv1679 Rv1933c Rv1934c Rv2724c Rv2789c Rv3061c Rv3139 Rv3140 Rv3274c Rv3504 Rv3505 Rv3543c Rv3544c Rv3560c Rv3562 Rv3563 Rv3564 Rv3573c Rv3761c Rv3797 Rv0672 Rv2500c Rv0222 Rv0456c Rv0632c Rv0673 Rv0675 Rv0971c Rv1070c Rv1071c Rv1141c Rv1142c Rv1472 Rv1935c Rv2486 Rv2679 Rv2831 Rv3039c Rv3516 Rv3550 Rv3774 Rv0905 Rv3374 Rv3373 Rv0468 Rv1715 Rv1912c Rv3141 Rv0243 Rv1074c Rv3546 Rv3556c Rv1323 Rv0859	Beta oxidation of unsaturated fatty acids

Continued on next page

Table 7 – continued from previous page

Reaction	Description	Formula	Genes	Pathways
FAD-19	acyl-CoA dehydrogenase fadE1-36 AND enoyl-CoA hydratase/isomerase echA1-21 AND 3-hydroxyacyl-CoA dehydrogenase fadB2-5 AND acetyl-CoA C-acetyltransferase fadA2-24	d2hdodcoa[c] + nad[c] -> dodcoa[c] + nadh[c]	Rv0131c Rv0154c Rv0215c Rv0231 Rv0244c Rv0271c Rv0400c Rv0752c Rv0873 Rv0972c Rv0975c Rv1346 Rv1467c Rv1679 Rv1933c Rv1934c Rv2724c Rv2789c Rv3061c Rv3139 Rv3140 Rv3274c Rv3504 Rv3505 Rv3543c Rv3544c Rv3560c Rv3562 Rv3563 Rv3564 Rv3573c Rv3761c Rv3797 Rv0672 Rv2500c Rv0222 Rv0456c Rv0632c Rv0673 Rv0675 Rv0971c Rv1070c Rv1071c Rv1141c Rv1142c Rv1472 Rv1935c Rv2486 Rv2679 Rv2831 Rv3039c Rv3516 Rv3550 Rv3774 Rv0905 Rv3374 Rv3373 Rv0468 Rv1715 Rv1912c Rv3141 Rv0243 Rv1074c Rv3546 Rv3556c Rv1323 Rv0859	Beta oxidation of unsaturated fatty acids
ALDR	Aldolase reductase	arab-D[c] + nadph[c] + h[c] -> arabl-D[c] + nadp[c]		Arabinogalactan Synthesis
AL4D	D-arabinitol 4-dehydrogenase	arabl-D[c] + nad[c] -> xylu-D[c] + nadh[c] + h[c]	Rv1928c	Arabinogalactan Synthesis

14 Table - S22 Metabolic Persister Reactions, Genes and Pathway on Rv3790 Deletion

Table 8: Metabolic persister reactions, gene and pathways on Rv3790 deletion

Reaction	Description	Formula	Genes	Pathways
ADK1	adenylate kinase	amp[c] + atp[c] <=> 2 adp[c]	Rv0733	Purine Metabolism
AMMQT8-2	S adenosylmethione2 demethylmenaquinone methyltransferase	2dmmq8[c] + amet[c] -> ahcys[c] + h[c] + mqn8[c]		Ubiquinone Metabolism
ARI	arabinose ribose isomerase	arpp[c] <=> prpp[c]		Membrane Metabolism
CO2t	CO2 transporter via diffusion	co2[e] <=> co2[c]		Transport
DCPDPP2	decaprenylphosphoryl arabinose phosphate dephosphatase	decdpa-tb[c] + h2o[c] -> decda-tb[c] + pi[c]		Membrane Metabolism
DCPT2	phosphoribose transferase undecaprenyl phosphate	arpp[c] + decd-tb[c] -> decdpa-tb[c] + ppi[c]		Membrane Metabolism
DHNAOT	1 4 dihydroxy 2 naphthoate octaprenyltransferase	dhna[c] + octdp[c] -> 2dmmq8[c] + co2[c] + h[c] + ppi[c]	Rv0534c	Ubiquinone Metabolism
EX-co2(e)	CO2 exchange	co2[e] <=>		Exchange
FORMCOAL	formyl coa CoA ligase	atp[c] + coa[c] + for[c] <=> amp[c] + forcoa[c] + ppi[c]		Redox Metabolism
FRD2	fumarate reductase	fum[c] + mql8[c] -> mqn8[c] + succ[c]	Rv0247c Rv0248c Rv3316 Rv3317 Rv3318 Rv3319	Citric Acid Cycle
GLXO1	glyoxylate oxidase	glx[c] + h2o[c] + nad[c] -> 2 h[c] + nadh[c] + oxa[c]		Glyoxylate Metabolism
HSDy	homoserine dehydrogenase NADPH	hom-L[c] + nadp[c] <=> aspsa[c] + h[c] + nadph[c]	Rv1294	Glycine Serine and Threonine Metabolism
HSK	homoserine kinase	atp[c] + hom-L[c] -> adp[c] + h[c] + phom[c]	Rv1296	Glycine Serine and Threonine Metabolism
ICHORSi	Isochorismate Synthase	chor[c] -> ichor[c]	Rv3215	Ubiquinone Metabolism

Continued on next page

Table 8 – continued from previous page

Reaction	Description	Formula	Genes	Pathways
MANAT2	mannosyl acyl transferase	PIM2[c] + pmtcoa[c] -> Ac1PIM2[c] + coa[c]	Rv2611c	PIM Biosynthesis
NPHS	naphthoate synthase	sbzcoa[c] -> coa[c] + dhna[c]	Rv0548c	Ubiquinone Metabolism
OXACOAL	oxalate coa ligase	atp[c] + coa[c] + oxa[c] <=> amp[c] + oxalcoa[c] + ppi[c]		Glyoxylate Metabolism
OXCDC	Oxalyl CoA decarboxylase	h[c] + oxalcoa[c] -> co2[c] + forcoa[c]	Rv0118c	Glyoxylate Metabolism
SHCHCS	2 succinyl 6 hydroxy 2 4 cyclohexadiene 1 carboxylate synthase	ichor[c] + sucsal[c] -> 2shchc[c] + pyr[c]	Rv0555	Cofactor Metabolism
SSALy	succinate semialdehyde dehydrogenase NADP	h2o[c] + nadp[c] + sucsal[c] -> 2 h[c] + nadph[c] + succ[c]	Rv0234c Rv1731	Glutamate Metabolism
SUCBZL	o succinylbenzoate CoA ligase	atp[c] + coa[c] + sucbz[c] -> amp[c] + ppi[c] + sbzcoa[c]	Rv0542c	Ubiquinone Metabolism
SUCBZS	O succinylbenzoate CoA synthase	2shchc[c] -> h2o[c] + sucbz[c]	Rv0553	Ubiquinone Metabolism
THRS	threonine synthase	h2o[c] + phom[c] -> pi[c] + thr-L[c]	Rv1295	Glycine Serine and Threonine Metabolism
